# Rapid and Inexpensive Whole-Genome Genotyping-by-Sequencing for Crossover Localization and Fine-Scale Genetic Mapping

**DOI:** 10.1534/g3.114.016501

**Published:** 2015-01-13

**Authors:** Beth A. Rowan, Vipul Patel, Detlef Weigel, Korbinian Schneeberger

**Affiliations:** * Department of Molecular Biology, Max Planck Institute for Developmental Biology, 72076 Tübingen, Germany; † Department of Developmental Biology, Max Planck Institute for Plant Breeding Research, 50829 Cologne, Germany

**Keywords:** next-generation sequencing, recombination, hidden Markov model, genetic mapping, quantitative trait

## Abstract

The reshuffling of existing genetic variation during meiosis is important both during evolution and in breeding. The reassortment of genetic variants relies on the formation of crossovers (COs) between homologous chromosomes. The pattern of genome-wide CO distributions can be rapidly and precisely established by the short-read sequencing of individuals from F_2_ populations, which in turn are useful for quantitative trait locus (QTL) mapping. Although sequencing costs have decreased precipitously in recent years, the costs of library preparation for hundreds of individuals have remained high. To enable rapid and inexpensive CO detection and QTL mapping using low-coverage whole-genome sequencing of large mapping populations, we have developed a new method for library preparation along with Trained Individual GenomE Reconstruction, a probabilistic method for genotype and CO predictions for recombinant individuals. In an example case with hundreds of F_2_ individuals from two *Arabidopsis thaliana* accessions, we resolved most CO breakpoints to within 2 kb and reduced a major flowering time QTL to a 9-kb interval. In addition, an extended region of unusually low recombination revealed a 1.8-Mb inversion polymorphism on the long arm of chromosome 4. We observed no significant differences in the frequency and distribution of COs between F_2_ individuals with and without a functional copy of the DNA helicase gene *RECQ4A*. In summary, we present a new, cost-efficient method for large-scale, high-precision genotyping-by-sequencing.

Meiotic recombination serves an essential function for sexually reproducing eukaryotes by promoting the formation of physical linkages (chiasmata) between pairs of homologous chromosomes that facilitate segregation and ensure that each gamete receives a proper complement of chromosomes (Hall 1972; Hawley 1988). It also has a potential consequence for evolution because genetic material is exchanged reciprocally between the two parental chromosomes, allowing new combinations of alleles to be passed on to the next generation (Barton and Charlesworth 1998). These sequence exchanges require crossovers (COs) that occur as one of several possible outcomes of the repair of programmed double-strand breaks (DSBs) that are initiated at the beginning of meiosis [for recent reviews on this subject, see Phadnis *et al.* (2011) and Baudat *et al.* (2013)]. Although the number of DSBs vary over three orders of magnitude among taxa (Weiner and Kleckner 1994; Terasawa *et al.* 1995; Moens *et al.* 1997; Anderson *et al.* 2001; Buhler *et al.* 2007; Chelysheva *et al.* 2007; Sanchez-Moran *et al.* 2007; Vignard *et al.* 2007; Mets and Meyer 2009; Nottke *et al.* 2011), the number of COs is typically limited to only one or two per chromosome pair per meiosis (Anderson *et al.* 1999, 2003; Pardo-Manuel De Villena and Sapienza 2001; Moens *et al.* 2002; Sanchez-Moran *et al.* 2002; Rosu *et al.* 2011; Salomé *et al.* 2011a; Segura *et al.* 2013; Barakate *et al.* 2014). It remains unclear how the proportion of DSBs that are resolved as COs is determined.

The distribution of COs across chromosomes is nonrandom, and both “hotspots” and “coldspots” have been documented [reviewed in Henderson (2012); Paigen and Petkov (2010); and Pryce and McFarlane (2009)]. In plants and fungi, COs tend to occur in regions of open chromatin (Mancera *et al.* 2008; Choi *et al.* 2013; Wijnker *et al.* 2013). Because it has traditionally been laborious and expensive to compare the exact genome-wide distributions of COs, transgenic recombination reporters have been used as an alternative, with the disadvantage that the number of genomic regions that can be examined is limited (Melamed-Bessudo *et al.* 2005; Francis *et al.* 2007). With advances in high-throughput sequencing, one can easily obtain large amounts of whole-genome resequencing data (Mardis 2008; Sboner *et al.* 2011), but the costs of library production and sequencing are often a barrier to studying hundreds of individuals (Table 1). This has led to the development of several methods for genotyping-by-sequencing (GBS), where reduced representation of the genome is used to rapidly genotype a large number of individuals. Because these methods rely on markers near specific restriction endonuclease sites (Baird *et al.* 2008; Andolfatto *et al.* 2011; Elshire *et al.* 2011), they yield poorer resolution at CO positions than whole-genome sequencing.

**Table 1:** Costs of library preparation for 96-sample multiplexing with small quantities of DNA

	Illumina Tru-Seq Nano	Our Protocol
Price per sample	$35.81	$5.20
Price for 384 samples	$13,751	$1978
Special equipment needed	Covaris S2,S220, E210, or M220	
	Bioanalyzer 2100 Desktop System	Bioanalyzer 2100 Desktop System
	System for real-time PCR	(System for real-time PCR)
	DNA fluorometer	DNA fluorometer
	Magnetic plate holder	Magnetic plate holder

Prices are based on the list price for the US market (in US dollars) and exclude the costs of the special equipment listed, plastic consumables such as 96-well PCR plates, optional steps and general laboratory reagents. Special equipment bracketed in parentheses indicates optional equipment needed for library quantification for normalization. See Table S3 for cost details. PCR, polymerase chain reaction.

Here, we present a GBS method in which the full genomes of hundreds of individuals can be resequenced inexpensively. Our efforts resulted in a wet-lab and analytical pipeline that can precisely detect CO positions even when coverage is sparse and be used for about a seventh of the cost of commercial whole-genome sequencing kits (Table 1). We used this pipeline to investigate whether RECQ4A, whose homologs SGS1 and BLM help resolve recombination intermediates in yeast and humans [Hartung *et al.* 2007; Holloway *et al.* 2010; De Muyt *et al.* 2012; reviewed in Knoll and Puchta (2011)], affects the distribution and frequency of COs in *Arabidopsis thaliana*. Yeast *sgs1* mutants exhibit elevated COs and abnormal meiotic progression (Rockmill *et al.* 2003; Jessop *et al.* 2006), and introduction of the *A. thaliana RECQ4A* protein partially restores the meiotic defects of yeast *sgs1* mutants (Bagherieh-Najjar *et al.* 2005). The frequency of somatic COs is two- to sevenfold greater in *recq4a* mutants of *A. thaliana* (Bagherieh-Najjar *et al.* 2005; Hartung *et al.* 2007). Although RECQ4A localizes to future sites of COs during meiosis, it does not necessarily seem to be required for CO formation, but rather to resolve telomeric bridges that arise in meiotic cells (Higgins *et al.* 2011). Such evidence both for and against a role for *recq4a* in CO formation prompted us to further investigate its role during meiosis.

Because the CO landscape is important for the genetic mapping of quantitative trait loci (QTL) (Xu *et al.* 2005), we also examined the fine-scale locations of COs and performed QTL mapping of flowering time in wild-type and *recq4a* mutant F_2_ populations. This examination revealed an extended region almost devoid of apparent CO events, which led us to uncover a 1.8-Mb inversion distinguishing the genomes of the parental lines.

## Materials and Methods

### Plant growth

Seeds were stratified for 4–7 d in 0.1% (w/v) agarose in water at 4°. All plants were grown in soil in 14-pot trays at 23° on 16-hr light/8-hr dark cycles at 65% relative humidity. We limited the number of plants to 12 per pot by sowing seeds in 12 positions and thinning the plants after 1 wk of growth by selecting only the top-most plant in a single sowing position (regardless of size or appearance). Plants were covered with a clear plastic dome for 1 wk after sowing. Crosses were performed using wild-type Ws-2 or *recq4a-1* (Ws-2 background) as the female parent and wild-type Col-0 or *recq4a-4* (Col-0 background) as the male parent (Supporting Information, Figure S1). The resulting F_1_ plants were allowed to self-fertilize, and the resulting F_2_ plants were grown in four staggered replicate experiments. A fifth replicate for *MAF4* genotyping was grown separately.

### Flowering time measurements

Beginning at 14 d after sowing, all plants among the four replicates were observed once per day until all plants flowered. The plants for *MAF4* genotyping were only observed every other day. When the inflorescence shoot reached the height of 1 cm, the number of rosette leaves and the number of days after sowing were recorded. Statistical analyses of flowering time data were performed using R version 3.0.2 (http://www.r-project.org).

### DNA extraction and quantification

For the Ws-2 parent, DNA was isolated from nuclei following the method described in Cao *et al.* (2011). For the F_2_ plants, 192 individuals from replicate number four were selected from each population and the tissue was frozen at −80° in 96-well block of 1.3-mL tubes with along with a 4-mm-diameter metal bead. The tissue was disrupted using a QIAGEN Mixer Mill (QIAGEN, Hilden, Germany) at 20 vibrations/sec for 2 min. DNA extraction was initiated by adding 500 µL of CTAB buffer [2% (w/v) hexadecyltrimethylammonium bromide, 1% (w/v) sodium bisulfite, 0.1 M Tris-HCl, 1.4 M NaCl, 20 mM ethylenediaminetetraacetic acid, pH 8] and incubating at 65° for 1 hr, followed by a flash spin before adding 500 µL of 24:1 chloroform:isoamyl alcohol. Samples were gently rocked for 10 min before centrifuging at 6000 × *g* in a Sigma 4K15C plate centrifuge (Sigma-Aldrich, St. Louis, MO) for 30 min. The upper phase was transferred to a 96-well plate containing 0.7 volumes isopropanol and incubated for 20 min at 4°, then centrifuged at 6000 × *g* in a Sigma 4K15C plate centrifuge for 20 min before a final wash with 70% ethanol and centrifugation at 6000 × *g* in a Sigma 4K15C plate centrifuge for 30 min. The ethanol supernatant was decanted and the DNA was allowed to dry completely before resuspension in MilliQ (Merck Millipore, Billerica, MA) water. Since we found that spectrophotometric measurements of DNA concentration were inaccurate for DNA extracted using the CTAB method, we used the Qubit 2.0 fluorometer with the high-sensitivity (HS) DNA quantification reagents (Invitrogen, Carlsbad, CA) to measure the DNA before normalizing all samples to approximately 100 ng before library preparation.

### Library preparation

For each sample, 100 ng of DNA were digested with 0.5 units of dsDNA Shearase (Zymo Research, Orange, CA) for 30 min at 37° in a total reaction volume of 30 µL. The reactions were stopped by adding ethylenediaminetetraacetic acid to a final concentration of 50 mM and then the DNA fragments were cleaned up using AMPure XP (Beckman-Coulter, Brea, CA) solid-phase reversible immobilization (SPRI) magnetic beads according to the manufacturer’s recommendations, except that the wash steps with 70% EtOH were performed with 200 µL and the samples were eluted in 18 µL of EB buffer (QIAGEN) during the final step. Eleven samples from each 96-well-plate were chosen for analysis of fragment size distribution using the Bioanalyzer 2100 Desktop System (Agilent Technologies, Santa Clara, CA) with the HS Kit (an example is given in Figure S2). The samples were then A-tailed at 37° for 30 min in a total volume of 25 µL using the Klenow exo-enzyme with 1X NEB Buffer 2, and 0.2 mM dATP (New England Biolabs, Ipswich, MA). The reactions were cleaned up using AMPure XP (Beckman-Coulter) magnetic SPRI beads according to the manufacturer’s protocol, except 70% EtOH washes were performed with 200 µL and the samples were eluted in 11 µL of 0.6X EB buffer (QIAGEN) without transferring the eluate to a fresh tube and leaving the SPRI beads in the reactions.

The A-tailed fragments served as input into reactions with 0.4 µM custom adapter mix (barcoded version of Illumina P1 adapter and standard Illumina P2 adapter, see Figure S3, Table S1), 1X ligation buffer, and 0.5 µL of Quick Ligase (New England Biolabs) and incubated for 15 min at 20°, followed by heat inactivation for 5 min at 65°. The custom adapters used in this step were created by mixing complementary single-stranded oligonucleotides at a concentration of 10 µM in 1X NEB Buffer 2 (New England Biolabs) and incubating in a water bath at 100° that was left to cool to room temperature over several hours. After the ligation step was completed, eight samples were pooled into a tube and concentrated using the AMPure XP (Beckman-Coulter) magnetic SPRI beads remaining in the tube according to the manufacturer’s protocol, except that 1.8 volumes of buffer containing 2.5 M NaCl and 8 mM polyethylene glycol was used in place of more SPRI beads and the samples were eluted in 30 µL EB buffer (QIAGEN) during the final step.

To ensure even representation of all samples, an optional quantitative polymerase chain reaction (PCR)-based quantification step can be performed using commercial reagent sets developed for your quantitative PCR system before pooling the samples together and performing size selection. For size selection, 15 µL of each eight-sample mix were combined into a single tube and 0.6 volumes of AMPure XP (Beckman-Coulter) magnetic SPRI beads were added to the tube. The samples were placed in a magnetic rack and allowed to clear. The supernatant was transferred to a fresh tube, and 0.2 volumes of AMPure XP (Beckman-Coulter) magnetic SPRI beads were added. The tubes were placed in a magnetic rack and allowed to clear before the supernatant was discarded, and the beads were washed twice with 70% ethanol and allowed to dry for 15 min at 37°. The libraries were then eluted according to the manufacturer’s procedure in a final volume of 30 µL of EB buffer (QIAGEN) and 3−15 µL was used in 50-µL PCRs with 0.2 mM standard Illumina primers and either the Phusion mastermix or the Phusion enzyme alone with 10X High Fidelity Buffer (all from New England Biolabs) and 0.2 mM dNTPs (Thermo Scientific Life, Waltham, MA). Reactions were incubated for 3 min at 72° and 30 sec at 98°, followed by 12 cycles of 10 sec at 98°, 30 sec at 65°, and 30 sec at 72° before a final incubation step at 72° for 5 min using an ABI GeneAmp PCR system 9700 (Applied Biosystems, Foster City, CA). Reactions were cleaned up using AMPure XP (Beckman-Coulter) SPRI magnetic beads according to the manufacturer’s recommendations, except that the samples were eluted in 30 µL of EB buffer (QIAGEN) in the final step. Final libraries were validated by quantification using the Qubit 2.0 fluorometer with the HS DNA quantification reagents (Invitrogen) and using the Bioanalyzer 2100 Desktop System with the DNA1000 Kit (Agilent Technologies). The library for the Ws-2 parent was prepared similarly, except 400 ng of DNA was used and all volumes and all library reagents were scaled up by a factor of two.

### Sequencing and initial data processing

Each 96-plex library for the F_2_ individuals was sequenced on an Illumina GAIIx analyzer (Illumina, San Diego, CA) in one flow cell lane using 2 × 150-bp length paired-end reads. Base calling was performed using the standard Illumina software to generate the raw reads, which were then filtered, demultiplexed, aligned to the TAIR10 reference genome for detection of sequence polymorphisms using the SHORE and GenomeMapper software (Ossowski *et al.* 2008; Schneeberger *et al.* 2009). Final average coverage for each 96-plex lane was 99.3x. The Ws-2 parent was resequenced similarly to a coverage depth of 25x.

To reduce genotyping errors that might arise from poor quality markers, we applied several stringent marker-filtering steps. Using the high-coverage resequencing data, we found 840,611 single-nucleotide polymorphisms (SNPs) between Ws-2 and Col-0 (TAIR10). After removing the SNPs located in the genomes of the mitochondrion and chloroplast and those close to insertion-deletion polymorphisms, we found that 745,273 SNPs remained. An additional 238,111 SNPs were removed after considering only those that were of high quality and supported exclusively by uniquely aligned reads. We further decreased the marker number to 302,082 after applying filtering for homozygous regions, transposons and coverage (accepting only those SNPs in which the local coverage was within two standard deviations of the mean genome-wide coverage). Finally, we used the segregation patterns of our markers in the F_2_ populations to remove 40,287 SNPs that did not show a Mendelian pattern of inheritance to obtain a final set of 261,795 markers (Figure S4).

### Genotyping at individual marker positions

We obtained the read counts for each individual at each of the SNP markers that passed our strict filters and measured the ratio of counts supporting the Col-0 or Ws-2 allele. We transformed these continuous ratios into a discrete specified alphabet including six states, which are AA, AU, AB, BU, BB, and UU. AA stands for homozygous Col-0, BB for homozygous Ws-2, AB for heterozygous states, U for the uncertainty to be called homozygous, and UU for no information. To transform the read counts into the alphabet states, we used a threshold of five reads supporting only one parental allele for assigning the homozygous genotypes (AA or BB) because a heterozygous position covered by five reads has less than a 5% chance of presenting only one allele. For genotypes in which both of the parental alleles were observed or less than five reads were aligned, we calculated the probability of a certain read count ratio to result from homozygous or heterozygous genotypes by using a multinomial distribution. We assumed that observing reads in a homozygous background (x_1_, x_2_) would follow a binomial distribution, where the probability for observing the parental allele is 99% (considering 1% sequencing errors). For a heterozygous background (f(a,b)) the probabilities for drawing reads from both parental genotypes would be equal to *P* = 0.5. We assigned the genotypes according to the maximum value between the homozygous (max(x_1_,x_2_)) and heterozygous probabilities (f(a,b)). If the maximum value was obtained for homozygous, we assigned the genotype AU (if x_1_ > x_2_) or BU (if x_1_ < x_2_); otherwise, it was labeled AB. The probabilities for drawing one of the parental genotypes (x_1_ or x_2_) in the homozygous background and the probabilities to draw them together coming from a heterozygous distribution (f(a,b)) have been estimated as

$$\begin{array}{l} x_{1}=\frac{\left(\mathrm{a+b}\right)!}{a!*b!}*0.99^{a}*0.01^{b}\\ x_{2}=\frac{\left(\mathrm{a+b}\right)!}{a!*b!}*0.99^{b}*0.01^{a}\\ f\left(a,b\right)=\frac{\left(\mathrm{a+b}\right)!}{a!*b!}*p^{a}*p^{b},p=0.5 \end{array}$$

where *a* and *b* describe the read counts for the respective parental alleles.

### State model of the hidden Markov modeling (HMM) and parameter estimations

Our HMM consisted of three hidden nodes (AA, AB, and BB), which reflected the three possible genotypes (Figure S5). In our model, all hidden nodes were connected, each of them featuring six emission states representing the observed alphabet of genotypes (see *Genotyping at individual marker positions* section above.).

After transformation of the read counts into our alphabet, transition and emission probability estimations were performed for each sample separately. We first estimated local allele frequencies along all chromosomes by applying a simple sliding window approach that estimates the local allele frequency for 1000 adjacent markers and reduces the noise in uncovering the distribution of the read counts. Although this could be used to assign genotypes to all markers (Huang *et al.* 2009), this does not allow for an accurate resolution of CO breakpoints. Ideally, the distribution of allele frequencies within the sliding windows should reflect a Mendelian distribution (*i.e.*, allele frequencies of 0, 0.5, and 1). However, due to the effects of random sampling, sequencing errors misalignments, and residual parental allele bias that may remain after marker filtering, the distribution is skewed. We fit three different beta curves to the observed allele frequency estimations, representing three different underlying allele frequency distributions introduced by the three different genotypes using a beta-mixture model with an expectation-maximization algorithm (Figure S6). The beta-mixture model approach is adapted from Ji *et al.* (2005), where the technique was introduced for identifying correlation of gene expression between different experiments. After fitting the three beta curves, we labeled each of the underlying allele frequency under each curve according to the expected genotype. The area under the curves is either limited by 0.1 or by the intersection of the neighboring beta curve. We then applied supervised learning strategy to obtain our probabilities by combining the labeled allele frequencies and our previously genotyped labels.

### Simulations and reconstructions of the F_2_ populations

To validate our pipeline, we simulated a mapping population with 5000 samples sequenced at three different coverage levels with a simulated sequencing error rate of 1%. Genotypes were generated at 261,795 high-quality SNP markers with the default recombination landscape from the Pop-seq software (Salomé *et al.* 2011a; James *et al.* 2013).

### Prediction and validation of predicted CO and inversion breakpoints

Structural variants in Ws-2 relative to the Col-0 reference were predicted using Pindel (Ye *et al.* 2009). For validation, we PCR amplified 1−2 kb of sequence spanning the predicted CO breakpoints. For the Ws-2 chromosome 4 insertion, we amplified ~500 bp to 1 kb around the predicted breakpoints. Genomic DNA was PCR amplified using reactions containing 0.4 µM forward and reverse primers, 0.2 µM dNTPs, 1x Phusion High Fidelity Buffer, and 0.2 units Phusion Taq (New England Biolabs) along with template DNA and incubated in an MJ Research PTC-200 or PTC-225 thermal cycler (Hercules, CA) at 94° for 2 min, followed by 37 cycles of 20 sec at 94°, 20 sec at 50−60°, and 30 sec at 72° with a final extension step at 72° for 5 min. Primer sequences are provided in Table S2. We sequenced the amplified regions in the Col-0 and Ws-2 parents and the F_2_ individuals with the predicted CO in sequencing by assembling reactions containing 1 µM primer, 0.5 µL of BigDye Terminator ready reaction mix, and 1X sequencing buffer and incubating for 20s at 96°, followed by 29 cycles of 10 sec at 50° and 4 min at 60° before analyzing on an ABI 3730xL sequencer.

### QTL analyses of flowering time

QTL analyses using the genotype data from marker blocks designated from the F_2_ genome reconstructions were performed using the R/qtl package for R version 3.0.2 (http://www.r-project.org/) designating the Kosambi map function and the expectation-maximization algorithm for all analyses. To determine significance thresholds for LOD scores, we performed 1000 permutations and set our threshold at an alpha of 0.05.

### MAF4 genotyping

We designed a cleaved, amplified polymorphic sequence marker (Konieczny and Ausubel 1993) using the Sol Genomics web interface (http://solgenomics.net/tools/caps_designer/caps_input.pl) based on the position 25995356, where the Ws-2 accession has a G nucleotide compared with the Col-0 reference A nucleotide that introduces a recognition site for the restriction enzyme *Sty*I. We genotyped 90 F_2_ individuals from each population (wild type and *recq4a*) using PCRs containing 0.4 µM forward and reverse primers, 0.2 µM dNTPs, 1x Phusion High Fidelity Buffer, and 0.2 units Phusion Taq (New England Biolabs). Reactions were incubated in an MJ Research PTC-200 or PTC-225 thermal cycler (Bio-Rad, Hercules, CA) at 94° for 2 min, followed by 37 cycles of 20 sec at 94°, 20 sec at 56°, and 30 sec at 72° and a final extension step at 72° for 5 min. Primer sequences are provided in Table S2.

## Results

### Rapid production of low-cost indexed libraries for paired-end-whole-genome sequencing

**Figure 1:**
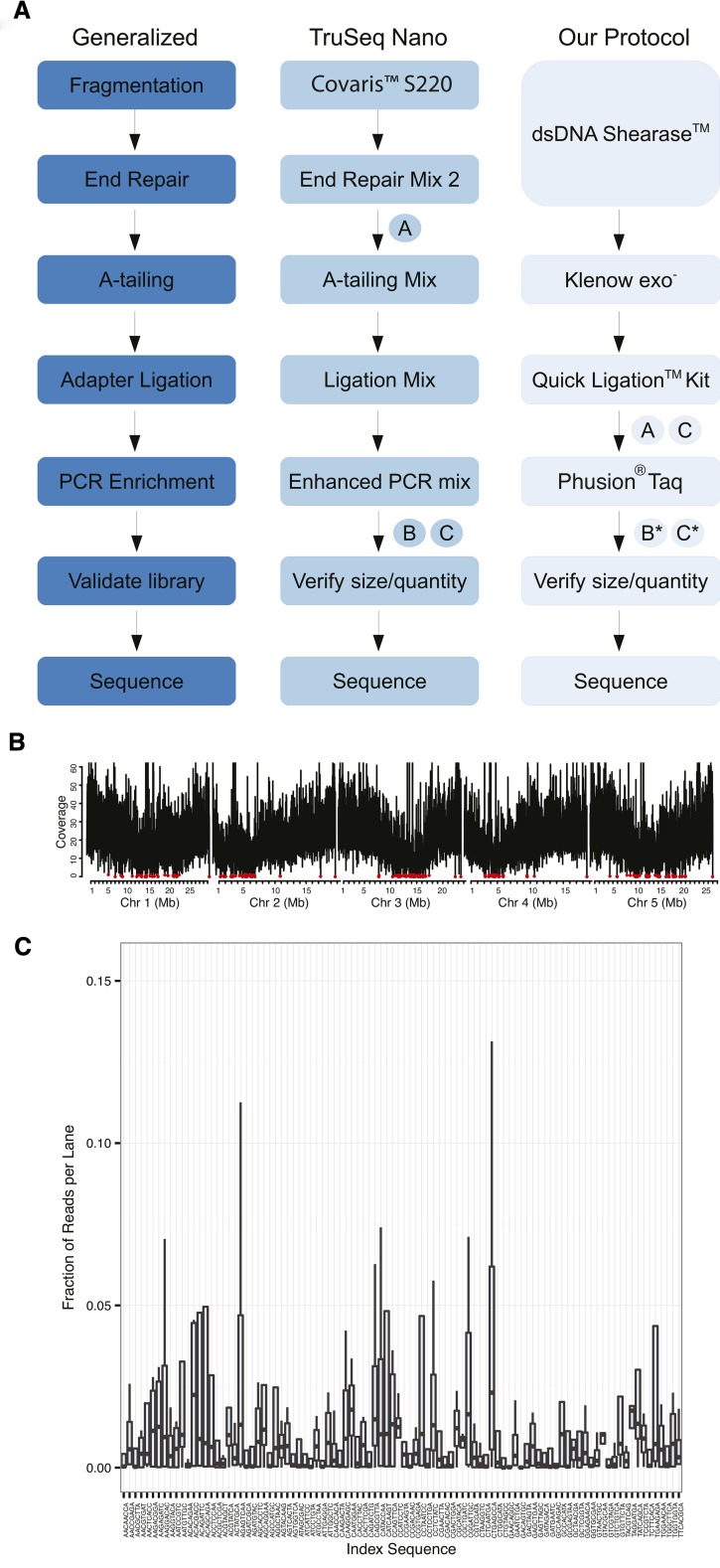
Library preparation workflow and sequencing coverage results. (A) Comparison of our protocol for the rapid production of paired-end libraries for whole-genome sequencing with Illumina TruSeq Nano protocol. “A” indicates size selection step, “B” indicates quantification/normalization step, and “C” indicates pooling step. * indicates an optional step. (B) Coverage distribution for reads generated from a single DNA library prepared for the *A. thaliana* accession Ws-2 using our high-throughput method and mapped against the Col-0 TAIR10 reference genome (including repetitive alignments). The average coverage in 5-kb bins is shown and the maximum coverage value has been capped to exclude the top 0.1% of average counts for each chromosome in order to compare all chromosomes at the same scale. Red circles indicate bins in which the average coverage was less than 1x. The genome-wide average depth of coverage was 25.8x. (C) The average representation of reads assigned to a specific index sequence over four separate multiplexed pools.

We developed a high-throughput method for preparing paired-end sequencing libraries for the Illumina platform that allows for multiplexing up to 96 individual DNA samples without the use of expensive sample preparation kits or specialized DNA fragmentation equipment, and with starting amounts of DNA as little as 100 ng per sample (Figure 1A). Compared with the closest commercial method, Illumina TruSeq Nano, our protocol produced libraries at about one-seventh of the cost (Table 1, Table S3).

We began library production with an enzymatic fragmentation step that generated a size range suitable for 300- to 500-bp insert libraries. Unlike restriction endonucleases, which reduce representation of the genome to tens of thousands of markers (or less), the recognition sites for the commercial enzyme formulation we used (dsDNA Shearase, Zymo Research) occur at 50,831,349 sites, or at almost every other base, across the *A. thaliana* genome (Figure S7). This allowed for the detection of hundreds of thousands of markers. To test whether fragmentation by dsDNA Shearase introduces a biased representation of the genome and to generate markers for the Ws-2 parent of our mapping populations, we scaled up the same protocol to produce libraries for Ws-2 whole genome resequencing to an average depth of about 25x. We found that the average coverage of reads that were aligned to the Col-0 reference genome was highest along the chromosome arms and lowest at the five centromeres (Figure 1B). There were only a few 5-kb bins in which the average coverage was less than one read, suggesting no substantial bias in the representation of the genome.

The fragmentation and clean-up (using magnetic beads for SPRI) for 96 samples processed simultaneously required less than one and a half hours total time. Although the fragment size distribution among samples was not completely uniform (Figure S2), the quantity of fragments in the targeted range of 300−500 bp was suitable for further processing. For A-tailing and ligation to custom-indexed adapters, we used standard enzymes (New England Biolabs). After the ligation step, we combined the 96 samples into a single pool without normalization, performed simultaneous size selection using SPRI beads, and enriched the single, multiplexed library by PCR. If a more even representation of all samples is desired, a normalization step can be included before pooling (note that this adds about $1−3 per sample, depending on the quantitative PCR platform and reagents needed for quantifying the libraries before pooling). The complete protocol (without the normalization step) requires only about a day and a half of laboratory work for 96 paired-end libraries starting from high molecular weight DNA.

### Low-coverage sequencing of hundreds of *A. thaliana* F_2_ individuals

To assess the meiotic role of *RECQ4A*, we generated two F_2_ populations: one derived from a cross between wild-type Col-0 and Ws-2 accessions and the other from the same parents, but with T-DNA insertions into *RECQ4A* that reduced gene function (Figure S8). We randomly selected 192 individuals from each population (1113 for wild type, and 1052 for *recq4a*, divided over four replicates), produced two 96-plex libraries (Figure S1), and sequenced each pool in a single lane of an Illumina GAIIx sequencer (Illumina). Our custom adapters contained an 8-bp index sequence with a T overhang (Figure S3). Without a normalization step, variation in the read counts for each index is most likely due to the molar representation of each library in the pool. Thus, we expect that any effect of the index sequence on ligation ability, PCR amplification, or cluster generation would be manifested as poor representation of that index sequence in all four pooled libraries. We compared the read counts for all 96 indexes across all four lanes (Figure 1C) and note that only three indices (GAACAGGC, GACAGTGC, ACACAGAA) were poorly represented in all lanes. The average number of reads per lane was almost 90 million, or an average of 1x genome-wide sequencing depth per sample. Almost half of the reads could be uniquely aligned to the reference genome (Figure S9). The bias, if any, introduced by the indices was thus negligible.

### GBS with sparse coverage using Trained Individual GenomE Reconstruction (TIGER)

**Figure 2:**
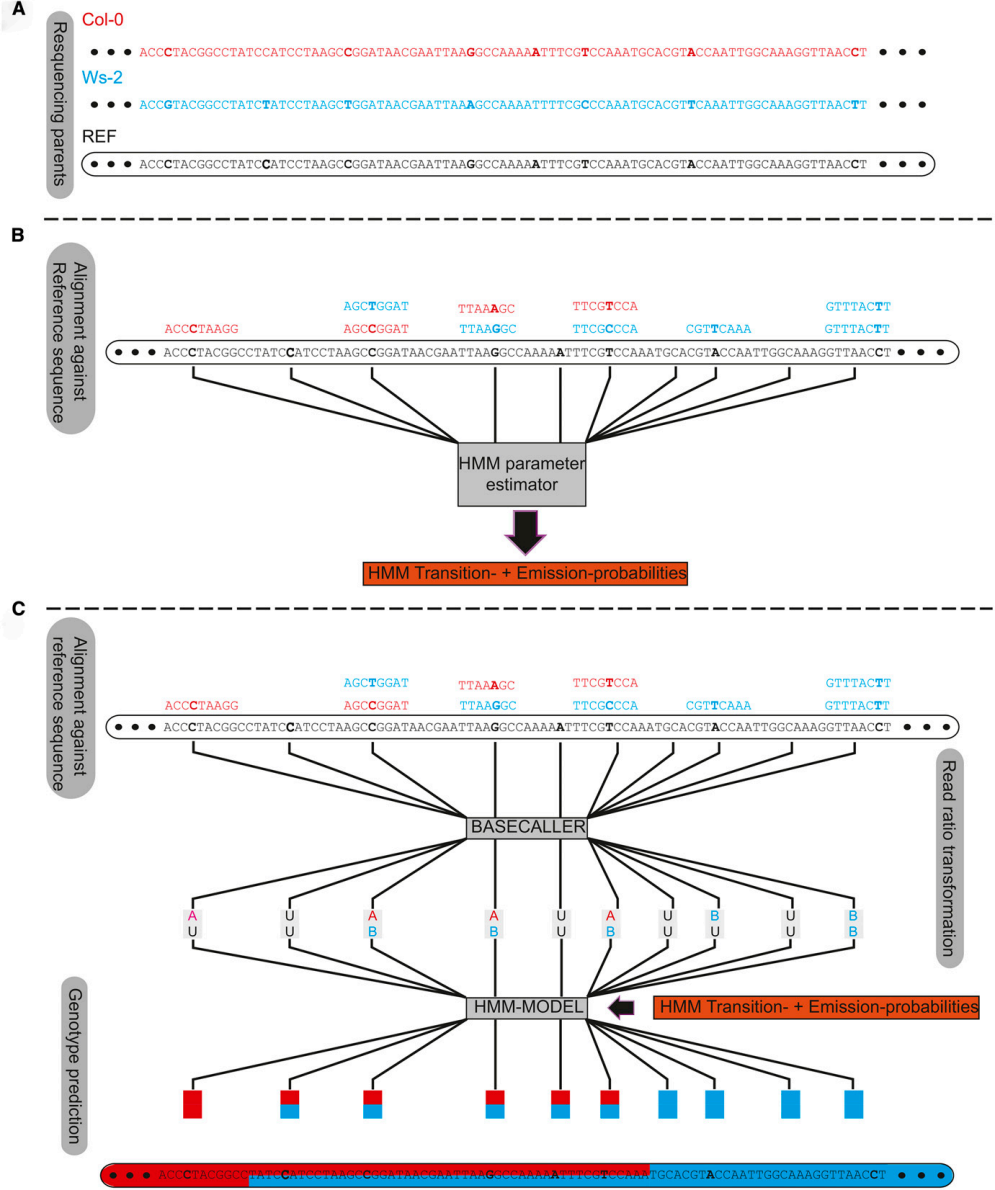
Genotyping by sequencing for sparse coverage sequencing from a biparental mapping population using Trained Individual GenomE Reconstruction (TIGER). The TIGER pipeline is summarized in (A−C). (A) Single-nucleotide polymorphisms between the two parents (red and blue) are determined relative to the reference sequence are localized and filtered. (B) The sequencing reads for each sample are aligned against the reference sequence and the read counts for both alleles at the filtered marker position are estimated. The read counts are used to estimate the probabilities for the transition and emission for a Hidden-Markov-Model by using a beta-mixture-model fit. (C) The read count ratios determined in (B) are transformed into an alphabet coding system using the Basecaller module of the TIGER pipeline. This alphabet consists of six states, AA, homozygous parent A (red); BB, homozygous parent B (blue); AB, heterozygous; AU or BU, weakly homozygous; and UU, no information at all. The output from the Basecaller with the outcome from the beta-mixture-model fit is used as input for our HMM which predicts the genotypes using the Viterby algorithm. Afterward we increase the crossover (CO) resolution by incorporating markers near the predicted CO position that were previously filtered out.

GBS with sparse coverage is difficult because of the limited information for assigning genotypes to a region. Tools developed for the field of human genomics (IMPUTE2, BEAGLE; Browning and Browning 2009; Howie *et al.* 2009) are designed for imputing haplotypes as found among individuals of natural populations and use internal parameters (recombination landscape or CO frequency) defined for human data. Andolfatto *et al.* (2011) and Xie *et al.* (2010) presented the first tools for GBS for artificial populations based on HMMs for sparse read coverage data. We improved their approaches by training the model using all reads at each marker position and avoiding the use of defined constants, implemented in TIGER, a GBS pipeline for reconstructing the mosaic genomes of recombinant individuals from sparse sequence coverage (Figure 2, A−C). We used strict marker filtering, followed by an HMM where transition and emission probabilities were estimated sample-wise using a beta-mixture model on the estimated parental allele frequencies within sliding windows. Allele frequencies were estimated by counting short read alignments at marker positions that supported either of the parental alleles. Because there were only three possible genotypes (parent A, parent B, and heterozygous), we expected three distinguishable curves along a chromosome, each having an independent peak. Two major factors introduced skews in this distribution: the effects of random sampling, and a bias for reference alleles. With the beta-mixture model, we could fit the observed distributions to the expected pattern (Figure S6); this information provided the input for the HMM (see the section *Materials and Methods*). This approach generates sample-specific probabilities that take the sample-specific-error-rate into account, allowing for more accurate genotype predictions from the HMM (see the section *Materials and Methods*).

### Evaluation of TIGER using simulation studies

**Figure 3:**
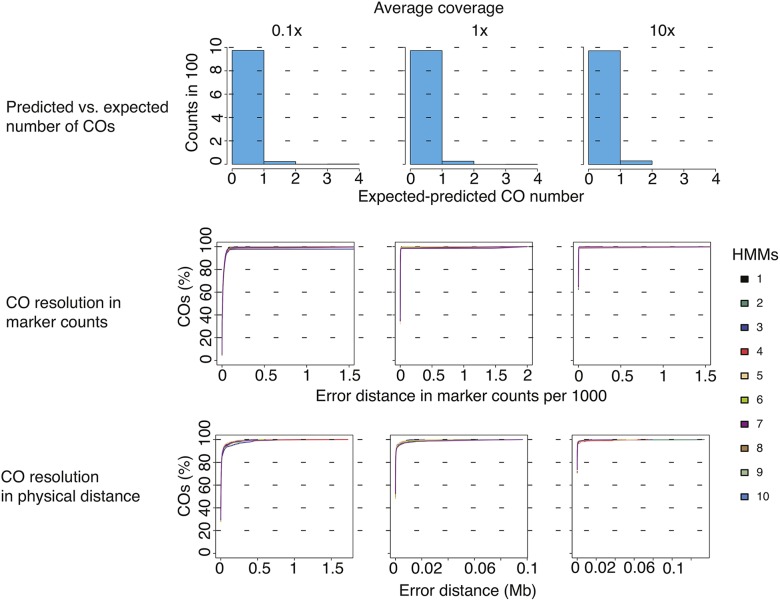
Evaluation of Trained Individual GenomE Reconstruction (TIGER) on simulated data. The TIGER pipeline was applied to simulated read data from 1000 simulated recombinant individuals for three different coverage rates. For each coverage rate, the samples were subset into 10 bins of 100 individuals and genotypes and crossovers (COs) were predicted using TIGER pipeline within the bins. The first row of plots shows the difference between the expected (simulated) and predicted CO numbers for each coverage rate. The second row of plots shows the resolution of COs in marker space for each of the 10 bins. The x-axes indicate the distance between the predicted and expected CO point based on the number of markers with a false genotype prediction and the y-axes show the percentage of total COs. The same representation is presented in the last row of plots, except the x-axes are measured in physical distances (Mb).

To validate our approach, we simulated three *A. thaliana* F_2_ mapping populations, each containing 1000 samples, with three different read coverage rates (0.1x, 1x, and 10x) using the published PopSeq tool (James *et al.* 2013; http://sourceforge.net/projects/popseq) based on recombination and sequence data from Salomé *et al.* (2011a). The 1000 samples were randomly distributed into 10 separate bins, and the number of predicted recombination events, the breakpoint resolution, and the types and genomic positions of errors were determined for each bin with TIGER (Figure 3).

We combined the results from 10 bins for each of the simulated coverage rates independently and compared the difference between the predicted and the expected number of COs. The difference between expectation and prediction was always positive, irrespective of coverage, indicating that our approach had the tendency to underestimate COs. As coverage increased, the percentage of expected COs that were predicted increased, from 97.5% at the lowest coverage level (0.1x) to 99.3% at the greatest (10x; Figure 3).

We estimated the resolution of COs by examining the physical distance and the number of markers between the predicted and the expected CO positions; as expected, resolution improved with increasing coverage (Figure 3). We could predict 90% of COs to within 2 kb of their actual position at all coverage levels. For the remaining COs, the distances depended on the distance between the CO and the closest marker that gave definitive information on a change in genotype along the chromosome. This uncertainty in assigning the marker closest to a CO was seven markers for 0.1x coverage, one for 1x, and none for 10x. The average resolution at 0.1x was 1986 bp (Table 2).

**Table 2:** CO breakpoint resolution using simulated data

	Marker Numbers		Physical Distance, kb		
Average Coverage	≤90%	≤98%	≤90%	≤98%	Median Resolution, bp
0.1x	38	79	27	222	1986
1x	4	10	4	94	938
10x	2	3	1	30	0

Evaluation of CO break point predictions using TIGER on data from a simulated mapping population of 1000 individuals at three different coverage levels. The distances (in marker number and physical length) for finding at least 90% or 98% of simulated COs are given. CO, crossover; TIGER, Trained Individual GenomE Reconstruction.

Examining the causes for errors in CO estimation, we found the most dominant error, about 90%, was the erroneous misclassification of heterozygous genotypes as homozygous. Most of these false homozygote prediction errors were in regions located within or next to the centromeres and telomeres. In these error-prone regions, the median false homozygote error rate was 2.4% for 0.1x coverage and approximately 1% for greater coverages (0.9%). The background error rate was 0.1% regardless of coverage (Figure S10). Differences in marker density, and errors in marker assignment because of repetitiveness of sequences, may account for the greater error rates near centromeres and telomeres. The marker density in the pericentromeric regions was high and abruptly dropped off at the border of the centromere (Figure S11). Importantly, Ws-2 and Col-0 homozygotes represented 42% and 47% of all errors, indicating that this type of error was not biased toward one of the two parental genotypes.

### Reconstructions of wild-type and *recq4a* F_2_ genomes

**Figure 4:**
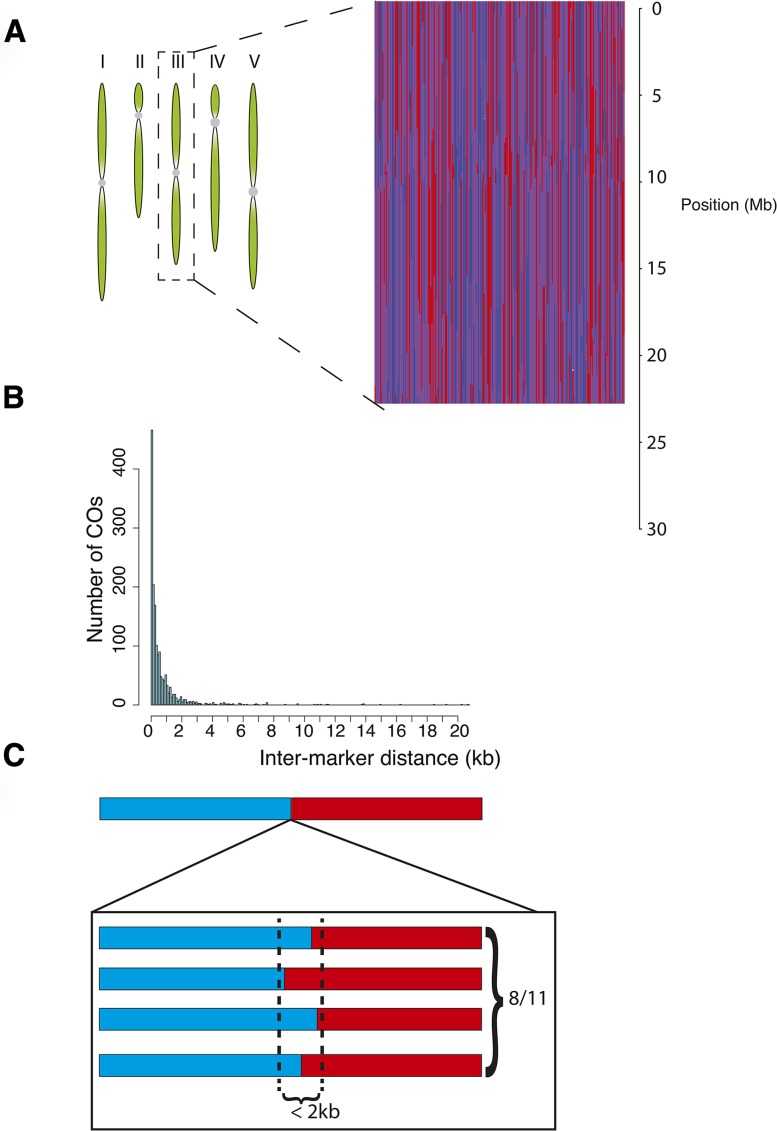
Genome reconstruction and crossover (CO) localization from experimental Ws-2 x Col-0 F_2_ populations. (A) A graphical example of reconstructions of chromosome 3 for 220 Ws-2 x Col-0 F_2_ individuals. Each vertical line represents a single individual. Red indicates homozygous for Col-0, blue homozygous for Ws-2 and heterozygous regions are in purple. (B) Histogram of the interval sizes for predicted COs. (C) Schematic representation for validation of CO intervals by PCR (Table 3).

We reconstructed the genomes of our F_2_ individuals using TIGER (an example is given in Figure 4A). From the high-coverage resequencing data for Ws-2, we obtained a set of high-quality markers for GBS after applying a strict set of filters (see the section *Materials and Methods*). We applied an average genome-wide coverage threshold of 0.025x to select individuals for the reconstructions because the accuracy of correct CO breakpoint predictions was strongly reduced below this threshold. We reconstructed the genomes of 110 individuals from the wild-type population and of 106 from the *recq4a* population; these had average and median coverages of 0.6x and of 0.4x. The overall frequency of Col-0, Ws-2, and heterozygous genotypes in both populations was consistent with a Mendelian pattern of inheritance (Table S4).

The simulation studies indicated several types of errors (Figure S10) produced by our approach, but in addition to the errors anticipated from the simulations, we encountered an error in the experimental F_2_ data in which small genotype blocks were embedded within larger blocks of a different genotype. These regions, termed “islands”, could have also represented real recombination events, as closely spaced double COs or gene conversions would have given rise to the same pattern. We used island length to distinguish errors from real recombination events. Of 67 islands, 7 were less than 400-kb long and were removed as errors (Figure S12). The remaining 60 islands were categorized as double COs because most gene conversion tracts are typically no longer than a few kilobases (Lu *et al.* 2011; Wijnker *et al.* 2013; Qi *et al.* 2014). After this additional error correction step, we had the final genome reconstructions to be used for further analyses. As with the simulated data, the CO predictions for the experimental data were not substantially affected by coverage (Figure S13). Using linear interpolation on masked markers near the breakpoint sites that had been previously filtered out, we resolved the majority of COs to an interval of less than 2 kb, as expected from the simulations (Figure 4B). Using PCR and Sanger sequencing, we confirmed that eight of 11 predicted COs had the expected genotype transition (Figure 4C and Table 3). Nearly all of these confirmed COs occurred within 1.5 kb of the predicted breakpoint, indicating that CO predictions using TIGER are within a few markers of the real CO position.

**Table 3:** Confirmation of CO positions in wt and *recq4a* populations

ID	Pop.	Plant ID	Chr.	Pos., bp	Up*^a^*	Down*^a^*	Fragment Size*^b^*	N*^c^*	First Marker*^d^*	Last Marker*^d^*	Confirmed?
1	wt	125	4	134,048	Col-0	Het	966	2	133,527	134,493	Yes
2	wt	145	4	16,276,940	Het	Col-0	1567	3	16,275,698	16,277,265	Yes
3	wt	147	1	29,632,761	Col-0	Het	1448	3	29,532,395	29,533,843	Yes
4	wt	125	4	10,419,728	Het	Col-0	675	4	10,419,347	10,420,022	Yes
5	wt	139	5	24,954,623	Het	Ws-2	784	5	24,954,069	24,954,853	No (Ws-2)
6	*recq4a*	231	3	4,957,859	Het	Ws-2	1118	6	4,957,322	4,958,440	Yes
7	*recq4a*	253	5	26,618,925	Het	Col-0	1351	2	26,568,249	26,569,600	No (Het)
8	*recq4a*	261	2	12,206,209	Ws-2	Het	779	3	12,206,209	12,206,988	No (Het)
9	*recq4a*	278	1	1,850,178	Het	Ws-2	1210	6	1,849,692	1,850,902	Yes
10	wt	308	3	172,226	Col-0	Het	1242	7	171,475	172,717	Yes
11	wt	387	3	10,014,511	Col-0	Het	1012	2	10,013,641	10,014,653	Yes

CO, crossover; wt, wild type; Pop., population; Chr., chromosome; Pos., position; PCR, polymerase chain reaction.

a Genotypes predicted up or downstream of the CO point.

b Size of the PCR fragment amplified.

c Number of markers covered by Sanger sequencing reads.

d Position of the first and last markers covered by the Sanger sequencing reads (in bp)

### *RECQ4A* does not affect the frequency or distribution of CO events in Col-0 x Ws-2 F_2_ populations

**Figure 5:**
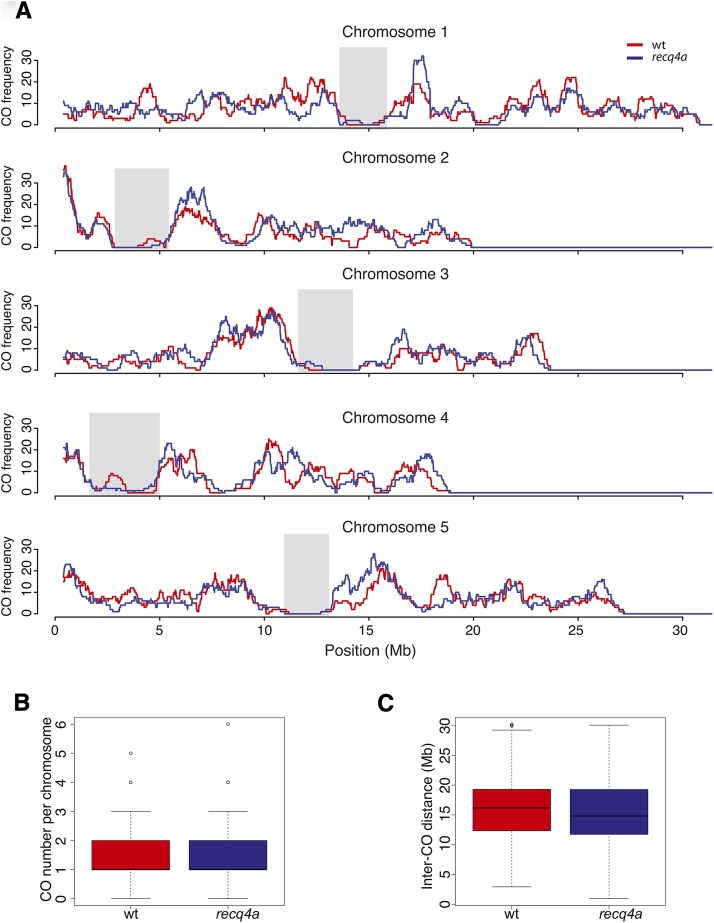
Comparison of crossover (CO) distribution and frequency in wt and *recq4a* F_2_ populations. (A) The CO rate over a sliding window of 800 kb for each of the five chromosomes. Regions shaded in gray correspond to centromeres. The CO numbers per chromosome (B) and lengths of genetic blocks created by the CO positions (C) are shown as box-and-whisker plots.

To observe an influence of RECQ4A on meiotic recombination, we first localized the positions of all CO breakpoints in the wild-type and *recq4a* populations. As has been previously observed (Copenhaver *et al.* 1999; Drouaud *et al.* 2007; Giraut *et al.* 2011; Salomé *et al.* 2011a), CO frequency increased from telomeres toward the centromeres, with centromeres themselves having very few COs, with little difference between wild-type and *recq4a* mutants (Figure 5A). *recq4a* mutants had on average slightly more COs per chromosome (1.52) than wild-type (1.46) (Figure 5B), but this difference was not statistically significant (Wilcoxon test *P*-value 0.32). The mean length of the genotype blocks generated by COs (Figure 5C) was slightly greater in wild-type (15.8 Mb) than in *recq4a* mutants (15.2 Mb), but this difference was also not significant (Wilcoxon test *P*-value 0.15). The mean distance between double COs that occurred on the same chromosome, or the inter-CO distance, was not significantly greater in wild type either (8.7 *vs.* 8.4 Mb) (Figure S12B; Wilcoxon test *P*-value 0.33). Using 800-kb sliding windows, we observed a strong correlation between COs in the wild-type and *recq4a* populations (Figure S14). Because the recombination landscape for Col-0 X Ws-2 F_2_ plants (with or without functional *RECQ4A*) was similar to several previously described F_2_ populations (Salomé *et al.* 2011a), we conclude that the loss of *RECQ4A* has either no or only a very minor effect on the frequency or distribution of COs in *A. thaliana*.

### Region with unusual suppression of COs reveals a 1.8-Mb inversion

In addition to the centromeres, we observed several large regions largely or entirely devoid of COs on the chromosome arms. The most notable spanned from around 7 to 9 Mb on the long arm of chromosome 4. Because inversions can suppress recombination (Sturtevant 1921), we predicted structural variants in the high-coverage Ws-2 short read data; Pindel detected potential inversion breakpoints at positions 7,139,542 and 8,914,936 bp. We confirmed these by PCR (Figure S15, A and B**)** and Sanger sequencing, which revealed that the downstream breakpoint was coupled with an additional insertion of 389 bp, of which 337 bp had 83% similarity to the CACTA-like transposable element Ptta/En/Spm. PCR-based screening revealed that the inversion was not present in Ws-0 (Figure S15C). We found the closest COs to the inversion at 6,989,963 and 8,960,496, which were around 150 kb and 45 kb away from the actual inversion breakpoints. Thus, we were able to map the location of the inversion extremely accurately using only the CO information from a limited number of plants.

### QTL mapping of flowering time using recombination blocks as markers

**Figure 6:**
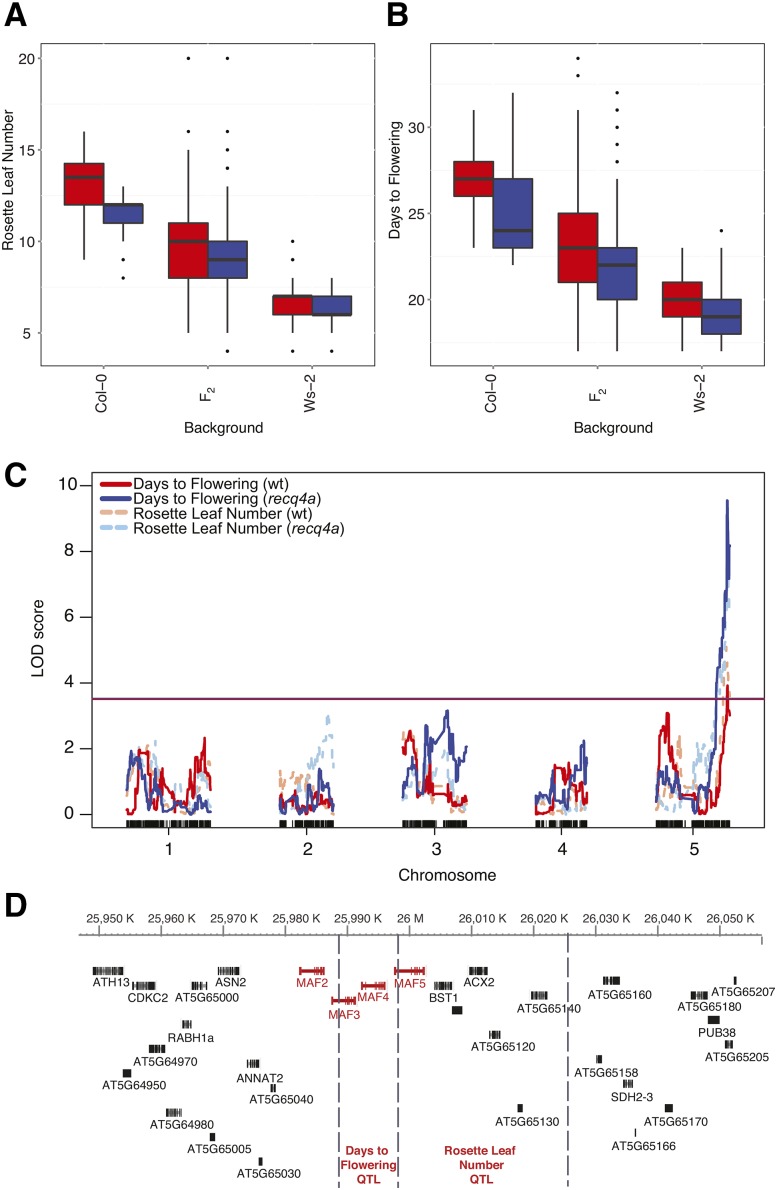
Quantitative trait locus (QTL) mapping of flowering time. Box-and-whisker plots showing rosette leaf number (A) and the days-to-flowering (B) for wild-type (red) and *recq4a* (blue) parents and among F_2_ individuals. (C) QTL analysis of flowering time phenotypes in the wt and *recq4a* mutant populations. The horizontal lines indicate the significance threshold (*P* = 0.05 for 1000 permutations) for the wt (red) and *recq4a* (blue) populations. Vertical ticks along the x-axis indicate the positions of the single-nucleotide polymorphism markers genotyped. (D) Schematic diagram of QTL intervals for flowering time within a region of chromosome 5 from 25,980,146 to 26,017,972 bp, based on data from the combined mapping populations from (B). Vertical dashed lines indicate the boundaries of marker blocks that were not separated by a recombination event in any F_2_ individual which are used to delineate the QTL intervals.

We used our methods to study the genetic architecture of flowering time phenotypes, days to flowering and rosette leaf number (see the section *Materials and Methods*), which are moderately correlated (R^2^ = 0.43) (Figure 6, A and B, Figure S16). On average, the wild-type Ws-2 parents flowered 7.5 d earlier and produced 6.6 fewer rosette leaves before flowering compared with wild-type Col-0 parents (Table S5). The *recq4a* mutation caused a small, but statistically significant acceleration of flowering, interacting with the Col-0 background (Table 4). This was also apparent in the distribution of flowering times among the F_2_ individuals.

**Table 4:** Flowering time as a function of genetic background and *RECQ4A* genotype

	DF	Sum of Squares	Mean Squares	F Value	*P*-Value
Days to flowering					
Background	1	3703	3703	1360.3	<2e-16***
* RECQ4A* genotype	1	140	140	51.4	5e-12***
Background x *RECQ4A* genotype	1	46	46	17.1	4.6e-05***
Rosette leaf number					
Background	1	2896	2896	2429	< 2e-16***
* RECQ4A* genotype	1	105.5	105.5	88.5	< 2e-16***
Background x *RECQ4A* genotype	1	31.8	31.8	26.7	4e-07***

Flowering time phenotypes were scored among the wt and *recq4a* parents for both the Col-0 and Ws-2 genetic backgrounds. See Table S5 for details. *** indicates statistical significance at the level of 0.01 or less. DF: degrees of freedom.

Because the *recq4a* mutation led to earlier flowering, we performed two separate QTL analyses of the wild-type and *rec4a* F_2_ populations (Figure 6C). We identified sequence blocks without recombination events in any F_2_ individual and chose markers on both ends of these blocks to represent the genotypes of the blocks (Figure S17). After removing 10 individuals with spurious genotype data from the wild-type population (Figure S18), both rosette leaf number and days-to-flowering mapped to a single major-effect QTL on the bottom of chromosome 5 in both populations (Figure 6C). Discarding markers with severe segregation distortion did not change this result (Figure S18). We also performed a QTL analysis with the combined wild-type and *recq4a* populations without removing any individuals and detected the same chromosome 5 QTL for both phenotypes (Figure S18). The combined analysis revealed a second, minor-effect QTL on chromosome 3.

To determine the potential causal genes underlying the major QTL on chromosome 5, we identified the boundaries of the recombination blocks containing the SNP marker with the highest association with flowering time for the combined population, since including more individuals resulted in more statistical power. For rosette leaf number, we identified a 26.9-kb interval (Figure 6D) where the associated SNP marker explained 16.7% of the phenotypic variation (Table S6). For days-to-flowering, the QTL interval mapped to an adjacent 9-kb block (Figure 6D), and the associated SNP marker explained 19.5% of the phenotypic variation (Table S6). Both intervals overlapped with the *MADS AFFECTING FLOWERING2-5 (MAF2-5)* gene cluster, which has been previously shown to be the causal factor underlying flowering time QTL in many different F_2_ populations (Alonso-Blanco *et al.* 1998; Weinig *et al.* 2002; Salomé *et al.* 2011b). We grew F_2_ plants again (N = 266 for wt and N = 202 for *recq4a*), scored both flowering time phenotypes, and genotyped 90 individuals from each population with a cleaved amplified polymorphic sequence marker (Konieczny and Ausubel 1993) designed for *MAF4*. There was a significant association between *MAF4* genotype and flowering time in both populations (Figure S19 and Table S7). We again observed that the *recq4a* mutation exerted a marginal effect on flowering time; however, there was no interactive effect between the *recq4a* mutation and the *MAF4* genotype (Table S7). In summary, we conclude that our whole-genome-GBS approach enables not only the fine-scale resolution of recombination breakpoints but also accurate and precise QTL mapping.

## Discussion

### An inexpensive and precise pipeline for studying recombinant individuals

The construction of precise genetic maps, which requires whole-genome resequencing of many recombinant individuals, is of critical importance to any researcher who is interested in studying the connection between genotype and phenotype. The current published and commercial methods make the production of high-density genetic maps cost-prohibitive for many investigators, who often compromise by adjusting either the marker sampling density or the number of individuals to be analyzed. With our method, whole-genome, paired-end genomic DNA libraries can be produced at about one-seventh of the cost of commercial alternatives (Table 1).

GBS of low-coverage samples is analytically challenging. Unlike the previously published methods using an HMM approach (Xie *et al.* 2010; Andolfatto *et al.* 2011), we used an HMM that was trained on each sample individually, estimating the error rate on the sample data instead of providing general parameters, and thus handling variation in error rates. Our method leverages the availability of a large number of high-quality markers distributed across the genome that segregate with a Mendelian pattern of inheritance and avoids bias of the HMM that occurs when only one parental allele per marker is considered (Xie *et al.* 2010; Andolfatto *et al.* 2011). We validated our approach using simulated data and characterized the types and locations of errors. We found that most errors were due to heterozygous genotypes not being properly predicted, particularly near telomeres and centromeres. We suspect that the errors at the telomeres likely arose from missing information because the ends of chromosomes had fewer markers. The centromeric regions had fewer high quality markers, which lowered the power of detecting a breakpoint nearby. The overall error rates were low (less than 3%) even at coverage of 0.1x, indicating that genotype and CO predictions were fairly robust.

Using TIGER, we could localize most CO positions to within 2 kb (Figure 4B), with most of the tested COs confirmed by PCR (Figure 4C and Table 3). This level of precision is more than 10-fold greater than what was previously achieved with reduced representation sequencing for *Drosophila melanogaster* (Andolfatto *et al.* 2011). The improved CO resolution is likely due to the combined effects of a more realistic representation of the genome, higher marker density and more accurate genotype predictions. In the future, even smaller resolution distances could be achieved by replacing the final linear filling process of incorporating filtered markers with a more flexible method (*e.g.*, an additional HMM model). We expect that similar resolutions would be achieved for species with a similar density of high-quality markers, and that precision would improve with higher coverage.

Our study has provided a detailed characterization of the CO landscape between Col-0 and Ws-2. We find that the overall landscape is similar to what had been observed previously for *A. thaliana* recombinant populations at low resolution (Copenhaver *et al.* 1999; Drouaud *et al.* 2007; Giraut *et al.* 2011; Salomé *et al.* 2011a), with the greatest CO frequency close to pericentromeric regions and a general absence of recombination within the centromeres (Figure 4A). In addition, we detected a 1.8-Mb inversion suppressing recombination from 7,139,542 to 8,914,936 bp on chromosome 4 in Ws-2 that is distinct from the much smaller inversion on the short arm of this chromosome that was reported by Fransz *et al.* (2000) and is not present in Ws-0 (Figure S14C). This adds to several reports that Ws-0 is genetically distinct from other Ws accessions (Aukerman *et al.* 1997; Anastasio *et al.* 2011; Pacurar *et al.* 2012).

Our precise CO breakpoint predictions enabled us to define blocks of the genome that were uninterrupted by COs in any F_2_ individual in both the wild-type and *recq4a* F_2_ populations. Using SNP markers that represented the genotypes of the blocks, we were able to precisely map a flowering time QTL to the *MAF2-5* gene cluster on chromosome 5 (Figure 6). The *MAF2-5* cluster has been identified as a QTL for flowering time variation for more than 20 pairs of accessions (Alonso-Blanco *et al.* 1998; Weinig *et al.* 2002; El-Lithy *et al.* 2004; El-Lithy *et al.* 2006; O’Neill *et al.* 2008; Simon *et al.* 2008; Salomé *et al.* 2011b). We note that 80% of recombination blocks were shorter than 106 kb, even though we had only a modest number of individuals (Figure S14).

### A role for REQ4A in Arabidopsis meiosis?

We expected that if *RECQ4A* normally acted to prevent meiotic COs, the frequency and possibly the distribution of CO events would be altered without *RECQ4A* function, similar to what has been reported for yeast (Rockmill *et al.* 2003; Jessop *et al.* 2006); this was not the case (Figure 5). The only significant effect of the *recq4a* mutation we observed was an acceleration of flowering time. Because this did not result from an interaction between the *recq4a* mutation and the flowering time QTL, *MAF2-5*, it is likely that the mild genotoxic stress in *recq4a* mutants promoted earlier flowering.

Why was there no significant effect of *recq4a* on the CO landscape? Higgins *et al.* (2011) also observed no increase in the frequency of chiasmata in *recq4a* mutants and suggested that this was either due to redundancy with another helicase or to *RECQ4A* only having anti-CO activity at interference-insensitive COs. We observed that the inter-CO distances between double COs on the same chromosome were generally shorter in the *recq4a* population, but this difference was not statistically significant. Because only a small fraction (15%) of COs is interference-insensitive (Higgins *et al.* 2004), a minor change in the number of interference-insensitive COs would likely not result in a statistically significant change in the total number or distribution of COs and would thus be undetectable. If *RECQ4A* acts only on this fraction of COs, its effect must be very small. If functional redundancy explains our observation that mutation of *RECQ4A* does not significantly impact the CO landscape, then it means that one of the other six RecQ helicases can substitute for it (Knoll and Puchta 2011). The RecQ helicase that is most similar is to RECQ4A is its paralog RECQ4B, which promotes COs and does not exhibit the same genetic interactions or DNA repair activities as RECQ4A in somatic cells (Hartung *et al.* 2006, 2007; Schropfer *et al.* 2014). Chiasma frequency in meiosis does not differ between wild type and *recq4b* mutants (Higgins *et al.* 2011), but the frequency of chiasma or COs in *recq4a recq4b* double mutants has not yet been assessed. Further work is needed to establish whether RECQ4B or one of the more distantly related RecQs can adopt anti-CO activity during meiosis when *RECQ4A* function is impaired.

Another intriguing possibility is that the resolution or dissolution of recombination intermediates during meiosis may not be mechanistically similar to what has been observed in other eukaryotes, as recently proposed by Knoll *et al.* (2014). These authors suggested that TOP3α and RMI1 (homologs of partner proteins for yeast SGS1 and human BLM) in *A. thaliana* might act on traditional double Holliday junction intermediates without RECQ4A or act with RECQ4A on different meiotic recombination intermediates. The lack of any significant effect of *recq4a* on the CO landscape in *A. thaliana* adds to the growing body of evidence for alternative methods for resolving recombination intermediates in plants.

Although there were no broad-scale changes in the distribution or frequency of COs due to the *recq4a* mutation, we believe that our approach can be used to assess the genome-wide effects of various mutants in the homologous recombination pathway. Our wet-lab and analytical pipeline will also enable cost-effective GBS of hundreds of individuals for QTL or association mapping, and thus removes a strong limitation to the scale of biological questions that can be asked and addressed.

## Supplementary Material

Supporting Information
