# Supplementary material for: Rapid and Inexpensive Whole-Genome Genotyping-by-Sequencing for Crossover Localization and Fine-Scale Genetic Mapping
Source: G3 (Bethesda). 2015 Jan 13;5(3):385–98. doi: 10.1534/g3.114.016501 (PMC4349092; doi:10.1534/g3.114.016501)
Supplement: Supporting Information [file supp_5_3_385__index.html]

Rapid and Inexpensive Whole-Genome Genotyping-by-Sequencing for Crossover Localization and Fine-Scale Genetic Mapping — Supporting Information 

# Rapid and Inexpensive Whole-Genome Genotyping-by-Sequencing for Crossover Localization and Fine-Scale Genetic Mapping

## Supporting Information for Rowan *et al.*, 2015

**Files in this Data Supplement:**

- Supporting Information - Tables S1-S7 and Figures S1-S19 (PDF, 2 MB)
- Table S1 - Adapter index sequences. (PDF, 61 KB)
- Table S2 - Primers used for PCR amplification and Sanger sequencing reactions. (PDF, 60 KB)
- Table S3 - Price comparison for individual components and reagents needed for paired-end library preparation. (PDF, 80 KB)
- Table S4 - Genotype frequencies in wt and *recq4a* F2 populations. (PDF, 103 KB)
- Table S5 - Summary of flowering time statistics for parental and F2 populations. (PDF, 103 KB)
- Table S6 - Additional statistics for QTL analyses of flowering time. (PDF, 105 KB)
- Table S7 - Flowering time as a function of *MAF4* and *RECQ4A* genotypes. (PDF, 105 KB)
- Figure S1 - Crossing scheme and workflow for mapping flowering time QTL. (PDF, 192 KB)
- Figure S2 - Fragment size distribution of Shearase™-digested *Arabidopsis thaliana* DNA. (PDF, 448 KB)
- Figure S3 - Design of custom adapters used for multiplexing. (PDF, 448 KB)
- Figure S4 - Ws-2 marker filtering workflow. (PDF, 115 KB)
- Figure S5 - Schematic representation of the HMM used in TIGER. (PDF, 176 KB)
- Figure S6 - Schematic workflow of the parameter estimator for the HMM. (PDF, 176 KB)
- Figure S7 - Distribution of dsDNA Shearase™ cutting sites across the genome. (PDF, 302 KB)
- Figure S8 - Expression of *RECQ4A* transcripts in wt parents, wt F2, and *recq4a* F2 individuals.
- Figure S9 - Coverage per sample and percentage of reads aligned. (PDF, 260 KB)
- Figure S10 - The frequency of different types of genotyping errors produced by TIGER using simulated data. (PDF, 128 KB)
- Figure S11 - SNP density between Col-0 and Ws-2. (PDF, 174 KB)
- Figure S12 - "Island" errors and double COs. (PDF, 217 KB)
- Figure S13 - The effect of coverage on CO prediction using TIGER. (PDF, 217 KB)
- Figure S14 - Correlation between CO distributions throughout the genome between wild-type and *recq4a* F2 populations. (PDF, 265 KB)
- Figure S15 - PCR amplification around predicted inversion breakpoints. (PDF, 143 KB)
- Figure S16 - Correlation between the number of days to flower and the number of rosette leaves at flowering. (PDF, 295 KB)
- Figure S17 - Recombination blocks used as markers for QTL analysis. (PDF, 165 KB)
- Figure S18 - Additional plots showing the results of QTL analyses for flowering time. (PDF, 483 KB)
- Figure S19 - Association between flowering time and *MAF4* genotype. (PDF, 239 KB)
