# Supplementary material for: Rapid and Inexpensive Whole-Genome Genotyping-by-Sequencing for Crossover Localization and Fine-Scale Genetic Mapping
Source: G3 (Bethesda). 2015 Jan 13;5(3):385–98. doi: 10.1534/g3.114.016501 (PMC4349092; doi:10.1534/g3.114.016501)
Supplement: Supporting Information [file supp_g3.114.016501_016501SI.pdf]

G3 · Genes | Genomes | Genetics

Rapid and inexpensive whole-genome genotyping-by-sequencing for crossover localization and fine-scale genetic mapping

Beth A. Rowan[§]*, Vipul Patel[‡]*, Detlef Weigel[§], and Korbinian Schneeberger[‡]

[§]Department of Molecular Biology, Max Planck Institute for Developmental Biology, Tübingen, Germany

[‡]Department of Developmental Biology, Max Planck Institute for Plant Breeding Research, Cologne, Germany

*these authors contributed equally to this work

The short read sequence data for this manuscript have been deposited in the National Center for Biotechnology Information Sequence Read Archive under accession number PRJNA268060.

Corresponding author
Detlef Weigel
Department of Molecular Biology
Max Planck Institute for Developmental Biology
Spemannstrasse 35
72076 Tübingen
Germany

Phone: +49 7071 601 1411
email: weigel@weigelworld.org

DOI: 10.1534/g3.114.016501

Table S1 Adapter index sequences.

Index Number	Sequence	Index Number	Sequence	Index Number	Sequence	Index Number	Sequence
1	ACGTAGCT	25	AGATCGCA	49	GATAGACA	73	AATGTTGC
2	TACGTCAG	26	AGCAGGAA	50	GCCACATA	74	ACACGACC
3	CGTCGATA	27	AGTCACTA	51	GCGAGTAA	75	ACAGATTC
4	GTAACTGC	28	ATCCTGTA	52	GCTAACGA	76	AGATGTAC
5	AACGTGAT	29	ATTGAGGA	53	GCTCGGTA	77	AGCACCTC
6	CGCTGATC	30	CAACCACA	54	GGAGAACA	78	AGCCATGC
7	CAGATCTG	31	CAAGACTA	55	GGTGCGAA	79	AGGCTAAC
8	ATGCCTAA	32	CAATGGAA	56	GTACGCAA	80	ATAGCGAC
9	CTGTAGCC	33	CACTTCGA	57	GTCGTAGA	81	ATCATTCC
10	AGTACAAG	34	CAGCGTTA	58	GTCTGTCA	82	ATTGGCTC
11	CATCAAGT	35	CATACCAA	59	GTGTTCTA	83	CAAGGAGC
12	AGTGGTCA	36	CCAGTTCA	60	TAGGATGA	84	CACCTTAC
13	AACAACCA	37	CCGAAGTA	61	TATCAGCA	85	CCATCCTC
14	AACCGAGA	38	CCGTGAGA	62	TCCGTCTA	86	CCGACAAC
15	AACGCTTA	39	CCTCCTGA	63	TCTTCACA	87	CCTAATCC
16	AAGACGGA	40	CGAACTTA	64	TGAAGAGA	88	CCTCTATC
17	AAGGTACA	41	CGACTGGA	65	TGGAACAA	89	CGACACAC
18	ACACAGAA	42	CGCATACA	66	TGGCTTCA	90	CGGATTGC
19	ACAGCAGA	43	CTCAATGA	67	TGGTGGTA	91	CTAAGGTC
20	ACCTCCAA	44	CTGAGCCA	68	TTCACGCA	92	GAACAGGC
21	ACGCTCGA	45	CTGGCATA	69	AACTCACC	93	GACAGTGC
22	ACGTATCA	46	GAATCTGA	70	AAGAGATC	94	GAGTTAGC
23	ACTATGCA	47	GACTAGTA	71	AAGGACAC	95	GATGAATC
24	AGAGTCAA	48	GAGCTGAA	72	AATCCGTC	96	GCCAAGAC

Sequences 1-4 were designed in house. Sequences 5 – 96 were taken from Mamanova et al. 2010.

Table S2 Primers used for PCR amplification and Sanger sequencing reactions

	Sequence (5' to 3')	CAPS enzyme	Allele cut
MAF Genotyping			
forward	ACTTCGTTCTTCACTACCGGT	StyI	Ws-2
reverse	GTTCTCCTCTCTCAGCAGCT	StyI	Ws-2
Ws-2 Inversion			
Primer 1	TGGCCTGCGTCTAAACAATG		
Primer 2	CCATCAATCTCAATTCACAAGGG		
Primer 3	ATGACCAGACCACTTTCCGG		
Primer 4	GACGTCGAGGCTGATAATTGA		
Primer 5	CGGGAAAGGACTGCTGAACT		
Primer 6	AACAGCAAAGCCACCATCAC		
C2 forward	TGGTCCGGTGGTGATTTACA		
C2 reverse	CGGCTTCTCGAGTTACTCTT		
Breakpoint resolution			
ID1 forward	GATGTTACCGTGGGTCGATT		
ID1 reverse	GTCTTCAACTTCCGGCGATA		
ID2 forward	CTTTGGCCGATAGACAGGAG		
ID2 reverse	TCAGCCACCACCACTACAAA		
ID3 forward	GCAGAAGATTCCGAGAGTGG		
ID3 reverse	TGGTCTTGGTCCGAAAATGT		
ID4 forward	TTCCCAATTTCGATCCAGAG		
ID4 reverse	CAGGCGGAGGAGTGTAAGAG		
ID5 forward	CCCGCCAAAATAAACAAAGA		
ID5 reverse	CACGAAGTTTCTCGGCTTTC		
ID6 forward	TGAACAGACAATTTGCTCCAA		
ID6 reverse	AGGCCAACGTTTAGGAGGAT		
ID7 forward	TTCCCTGCATCCTAGTCCTG		
ID7 reverse	AAAAGGAATGGCACACGTTC		
ID8 forward	CCGTCAATTTGAGCAGGAAT		
ID8 reverse	TTCTACGCACACCAGGGATT		
ID9 forward	GCGTAATCATCACTCGCTCA		
ID9 reverse	TGCAAACACGGAAGACAGTT		
ID10 forward	GCCATGCGTACCTGAAAAGT		
ID10 reverse	ATTGGCATCACGAGGAAAGT		
ID11 forward	CAATTAACAAGGCCGAGTAAAGA		
ID11 reverse	GGAATCTCCCTTGGTCCCTA		

Table S3 Price comparison for individual components and reagents needed for paired-end library preparation

Our Method

	Manufacturer	Part number	Price per package	Samples per package	Price per sample
**DNA fragmentation**					
dsDNA Shearase™*	Zymo Research	E2018-200	396	400	0.99
**A-tailing**					
Klenow exo-	New England Biolabs	M0212L	236	400	0.59
dATP	New England Biolabs	N0440S	48	5000	0.01
**Adapter Ligation**					
Custom adapter oligos	Sigma-Aldrich	NA	3840	576000	0.01
P2 adapter oligos	Sigma-Aldrich	NA	40	6000	0.01
Quick Ligation Kit™	New England Biolabs	M2200L	388	300	1.29
NEB Buffer 2	New England Biolabs	B7002S	18	384	0.05
**PCR Enrichment**					
Phusion Mastermix	New England Biolabs	M0531S	170	9600	0.02
Primer oligos	Sigma-Aldrich	NA	47	28800000	0.000002
**TOTAL for Reagents**					**2.96**
**Clean-ups**					
AmpPure XP Magnetic Beads	Beckman-Coulter	A63881	1126	508	2.22
**TOTAL for Library prep**					**5.18**
**Validation**					
Qubit DNA quantification reagents	Life Technologies	Q32851	79	9600	0.01
Bioanlayzer reagents	Agilent Technologies	5067-1505	349	28800	0.01
**TOTAL for Preparation/Validation**					**5.20**
**Pre-Library Prep Quantification**					
Qubit DNA quantification reagents	Life Technologies	Q32851	79	100	0.79
**TOTAL for all**					**5.99**

Illumina Tru-Seq Nano

	Manufacturer	Part number	Price per package	Samples per package	Price per sample
**DNA Fragmentation**					
	Covaris	520045	125	25	5
**Library Prep**					
TruSeq Nano	Illumina	FC-121-4003	2880	96	30
**TOTAL for Library prep**					**35**
**Validation**					
Qubit DNA quantification reagents	Life Technologies	Q32851	79	9600	0.01
Bioanlayzer reagents	Agilent Technologies	5067-1505	349	28800	0.01
**TOTAL for Preparation/Validation**					**35.02**
**Pre-Library prep Quantification**					
Qubit DNA quantification reagents	Life Technologies	Q32851	79	100	0.79
**TOTAL for all**					**35.81**

Prices are based on the list price for the US market (in US dollars). The reagents needed for library quantification for normalization purposes, plastic consumables such as 96-well PCR plates, and general laboratory reagents are not included, as they not included in the Illumina TruSeq kit.

Table S4. Genotype frequencies in wt and *recq4a* F_2 populations

	Col-0	Ws-2	Het
wt	28.0%	21.8%	50.2%
recq4a	23.8%	25.6%	50.7%

Table S5 Summary of flowering time statistics for parental and F_2 populations

Background	Genotype	N	Mean Days to Flowering (± sem)	Mean Rosette Leaf Number (± sem)
Col-0	wt	64	27.19 (± 0.18)	13.34 (± 0.19)
Col-0	*recq4a*	65	24.92 (± 0.32)	11.4 (± 0.14)
F_2	wt	1133	23.07 (± 0.07)	9.66 (± 0.05)
F_2	*recq4a*	1052	21.95 (± 0.07)	9.23 (± 0.06)
Ws-2	wt	66	19.65(± 0.16)	6.72 (± 0.14)
Ws-2	*recq4a*	136	18.96 (± 0.11)	6.1 (± 0.07)

Phenotypes were scored when the inflorescence shoot reached the height of 1 cm. Abbreviations used: SD, standard deviation; sem, standard error of the mean.

Table S6 Additional statistics for QTL analyses of flowering time

	Position (Mb)	LOD	Variance explained (%)
wt			
Rosette Leaf Number	25.55	5.09	20.7
Days to Flower	25.99	3.92	16.4
recq4a			
Rosette Leaf Number	26.02	8.73	24.6
Days to Flower	25.86	9.55	23.7
Combined			
Rosette Leaf Number	26.02	8.52	16.7
Days to Flower	25.99	10.25	19.5

Table S7 Flowering time as a function of *MAF4* and *RECQ4A* genotypes

	DF	Sum of Squares	Mean Squares	F value	P-value
**Days to Flowering**					
MAF4 Genotype	2	78.7	39.4	8.6	2.8e-4
RECQ4A genotype	1	16.3	16.3	3.6	0.06
RECQ4A x *MAF4* Genotype	2	0.8	0.4	0.09	0.92
**Rosette Leaf Number**					
RECQ4A genotype	2	91.7	45.8	26.5	9e-11
MAF4 Genotype	1	19.5	19.5	11.3	1e-3
RECQ4A x *MAF4* Genotype	2	6.8	3.4	1.95	0.15

Flowering time phenotypes were scored among F_2 individuals from the wt and *recq4a* populations that had been genotyped at the *MAF4* locus. Shown are the results of an analysis of variance (ANOVA). See Table S5 for summary statistics of the phenotypic data.

Literature cited

Mamanova, L., Coffey, A. J., Scott, C. E., Kozarewa, I., Turner, E. H., Kumar, A.., Howard, E., Shendure, J. Turner, D. 2010. Target-enrichment strategies for next- generation sequencing. Nature Methods *7*(2):111-118

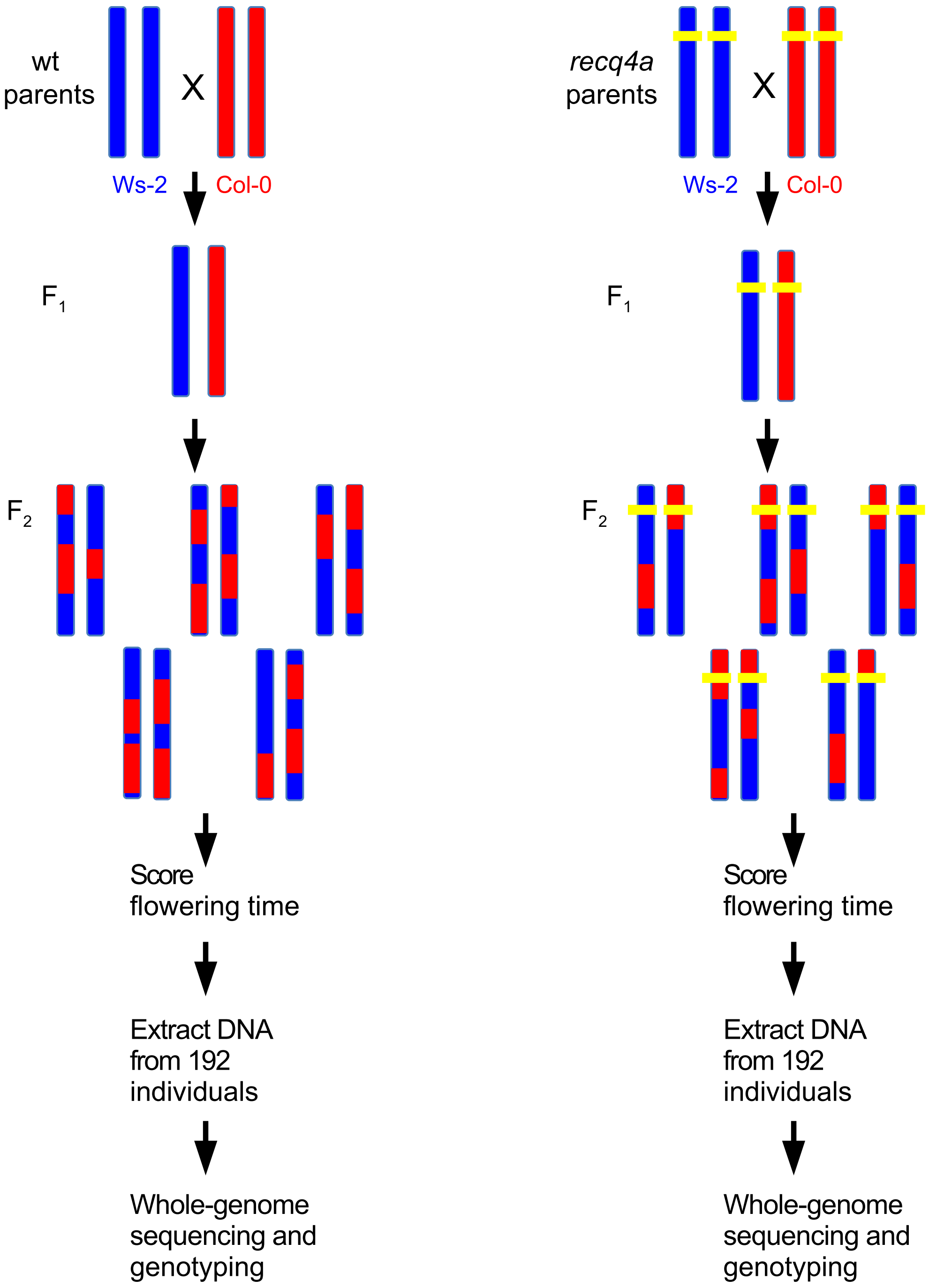

Figure S1. Crossing scheme and workflow for mapping flowering time QTL.

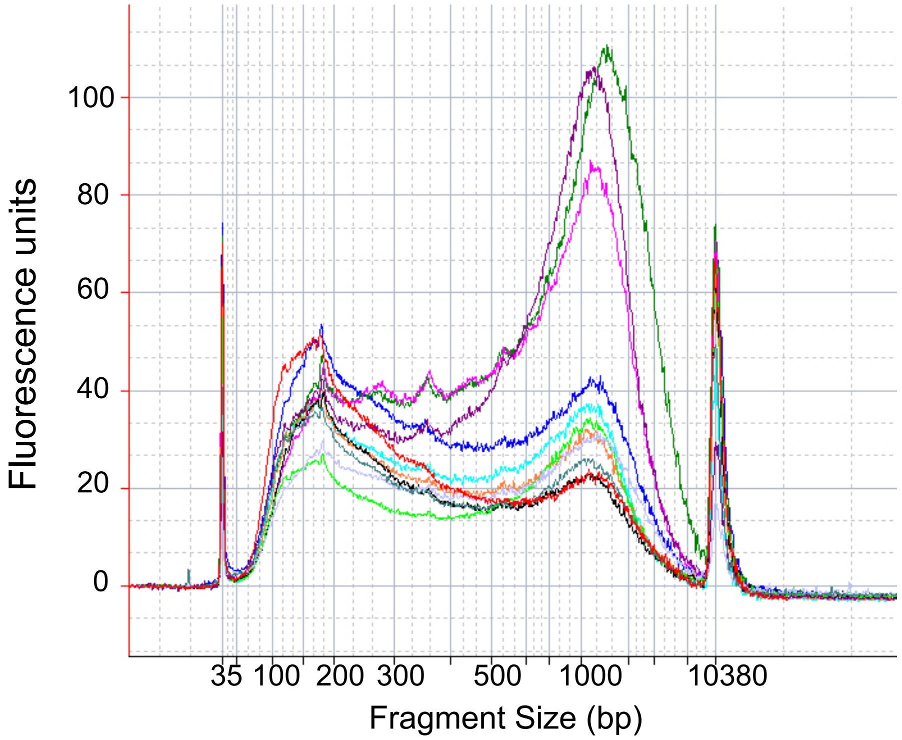

Figure S2 Fragment size distribution of Shearase™-digested Arabidopsis thaliana DNA. Bioanalyzer traces of 11 DNA samples from one 96-plex library prep after digestion with the Shearase™ enzyme are shown.

A

P1

INDEX1

5' ACACTCTTTCCCTACACGACGCTCTTCCGATCT***ACGTAGCT****T* (sense)

5'P-***AGCTACGT***AGATCGGAAGAGCGTCGTGTAGGGAAAGAGTG*T (antisense)

P2

5' P-GATCGGAAGAGCGGTTCAGCAGGAATGCCGA*G (sense)

5' CTCGGCATTCCTGCTGAACCGCTCTTCCGATC*T (antisense)

B

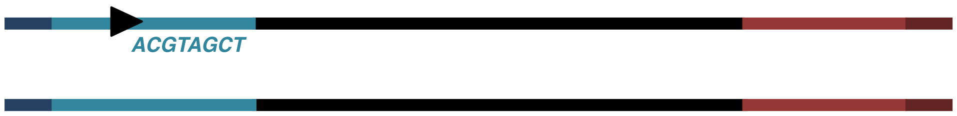

Figure S3 Design of custom adapters used for multiplexing. A) Oligo sequences for the indexing adapters. The index is added to the 3' end of the Illumina P1 adapter. An example index sequence (in italics) is shown. See Table S1 for all 96 index sequences. "P" is used to indicate a 5' phosphate addition. The * shows the position of a phosphorothioate bond. Oligo sequences for the universal Illumina P2 adapter are also shown. B) Schematic diagram showing adapters ligated to DNA fragments. The arrow indicates the direction of sequencing using the Illumina primer for the first read. The first 8 bases of sequence for read 1 are the index sequence.

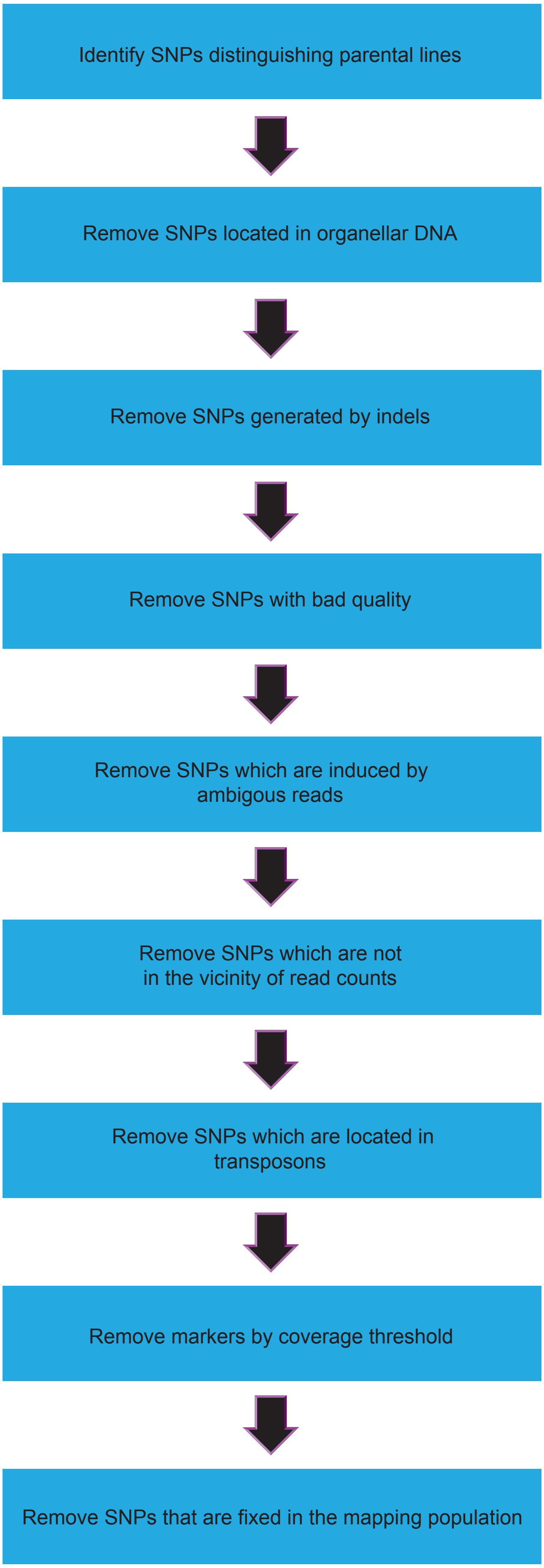

Figure S4 Ws-2 marker filtering workflow. Stepwise representation of the marker filtering pipeline used to select SNPs for downstream implementation in TIGER.

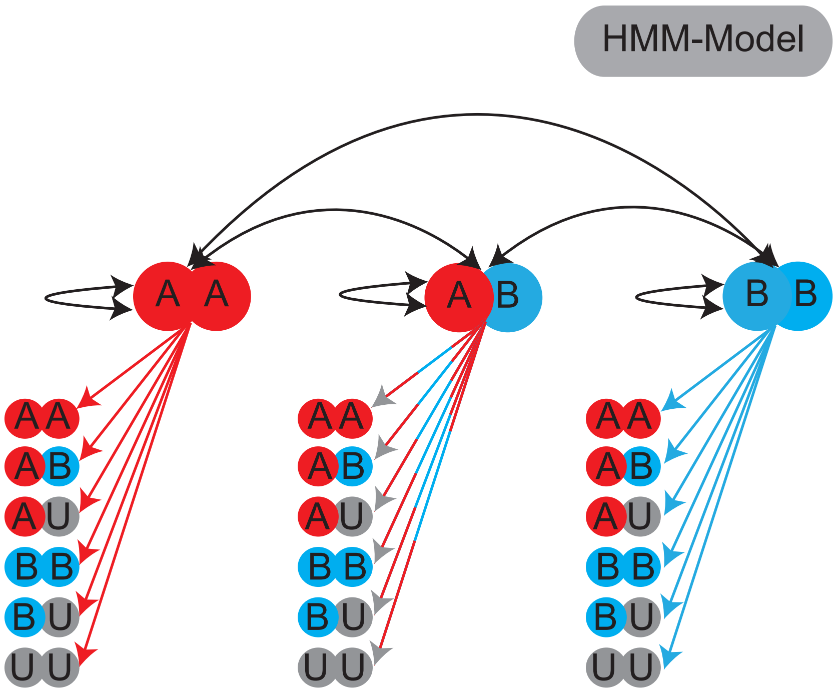

Figure S5 Schematic representation of the HMM used in TIGER. Red indicates parent one, blue indicates parent two and grey indicates no information (unknown).

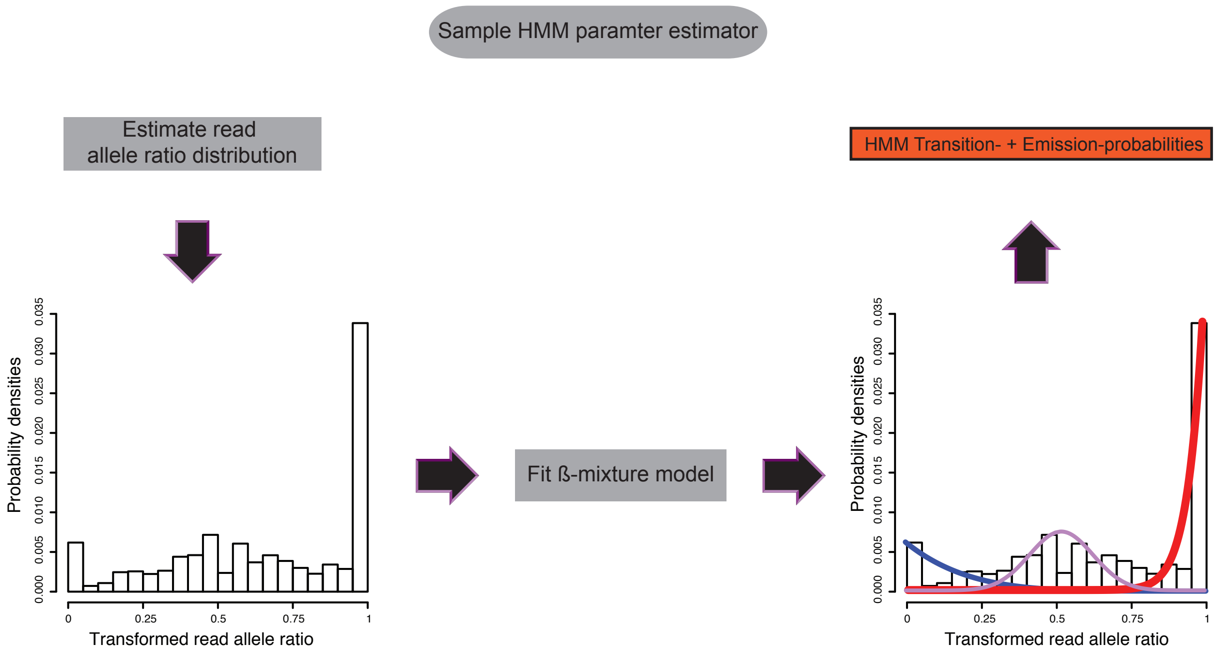

Figure S6 Schematic workflow of the parameter estimator for the HMM. For each sample the read allele ratio is estimated from the result of a sliding window approach. The resulting frequencies are then transformed to interval of 0 and 1 as the beta function is only defined at that region. Afterwards the beta-mixture model fit is applied. The probabilities for the sample-specific HMM are estimated from the resulting three curves and the output from the Basecaller.

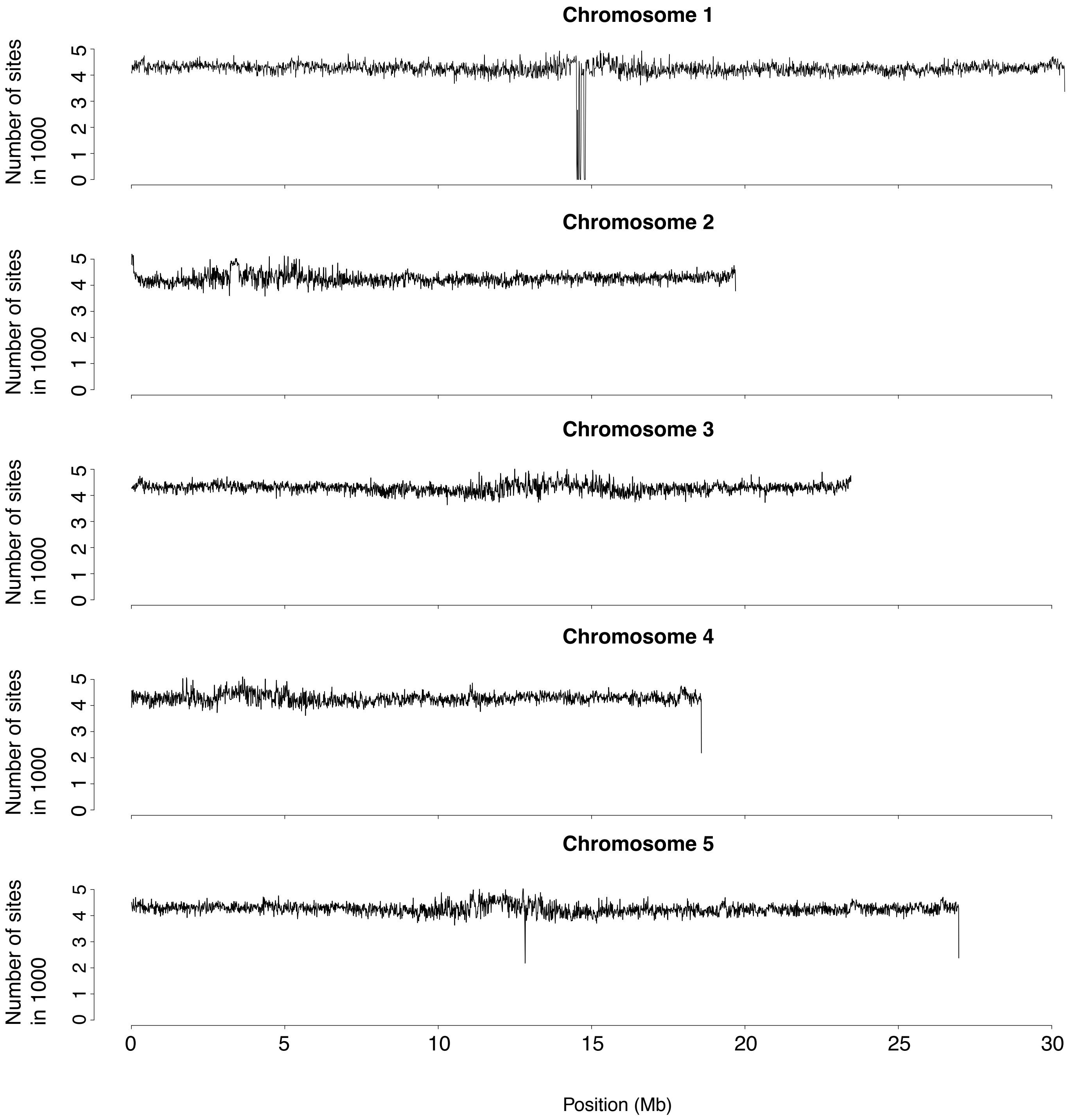

Figure S7 Distribution of dsDNA Shearase™ cutting sites across the genome. The enzyme recognizes a site with the degenerate sequence 5' NVBN 3', where N = any base, V = any base except for T, and B = any base except for A. The frequency of recognition sites across the *A. thaliana* genome over a 10 kb sliding window is shown.

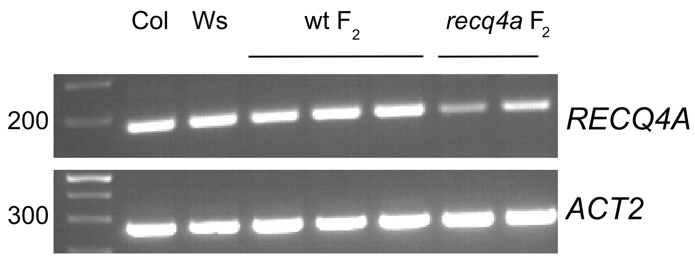

Figure S8 Expression of *RECQ4A* transcripts in wt parents, wt F_2 , and *recq4a* F_2 individuals. All samples are mixed-stage flowers from a single individual. Numbers on the left indicate the size of the fragments (in base pairs). The *ACT2* gene was used as a control.

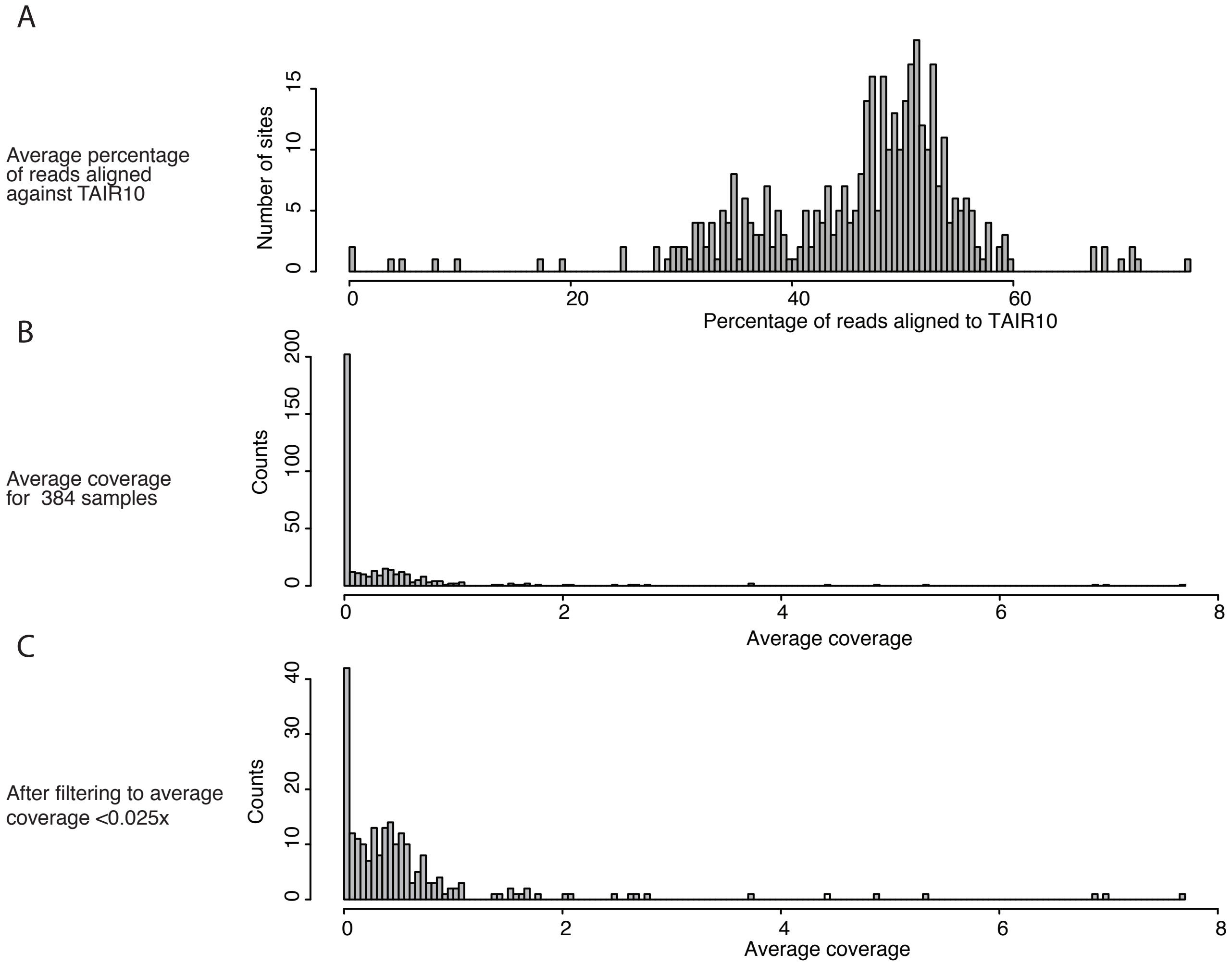

Figure S9 Coverage per sample and percentage of reads aligned. A) Average number of reads aligned to the used reference sequence TAIR10. The distribution of average read coverage (represented as the fold coverage of the *A. thaliana* reference genome) per sample is shown for all samples in B and for only samples where the average coverage was greater than 0.025x in C.

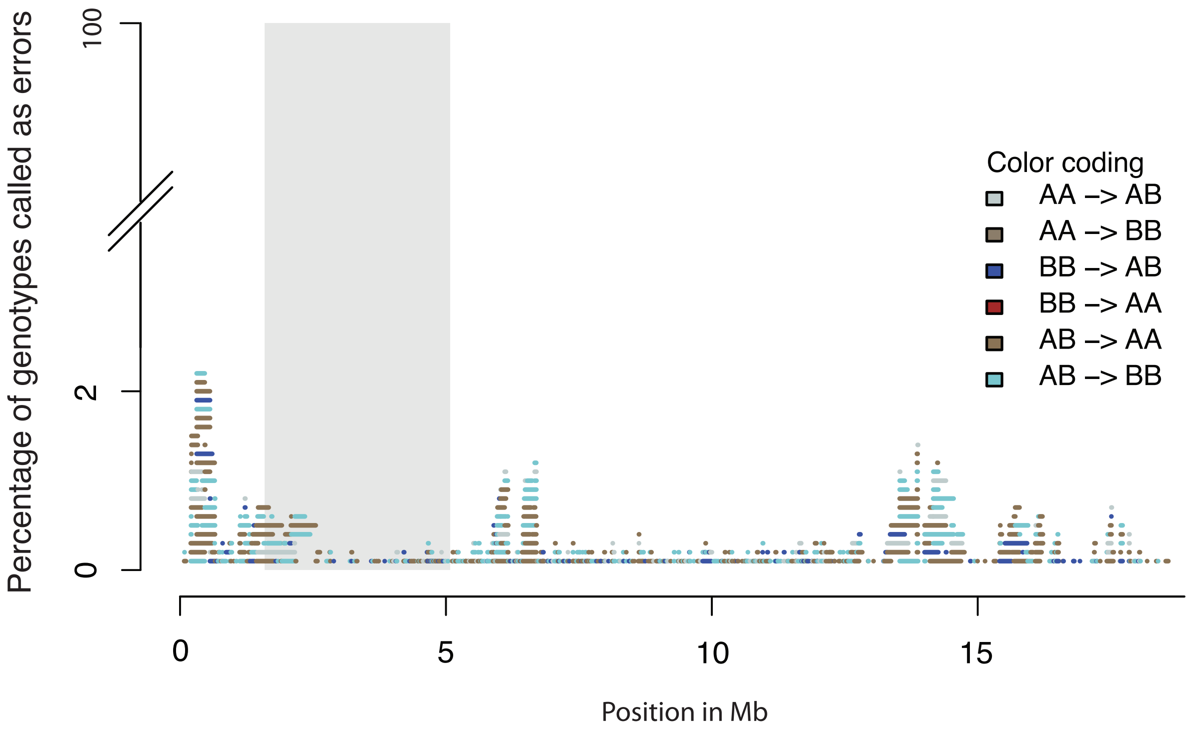

Figure S10 The frequency of different types of genotyping errors produced by TIGER using simulated data. An example of an error profile for chromsome 4 is shown (results were similar for the other four chromosomes). The grey box indicates the location of the centromere. The error frequencies are obtained from using TIGER to predict genotypes from random read data from 1000 simulated recombinant individuals.

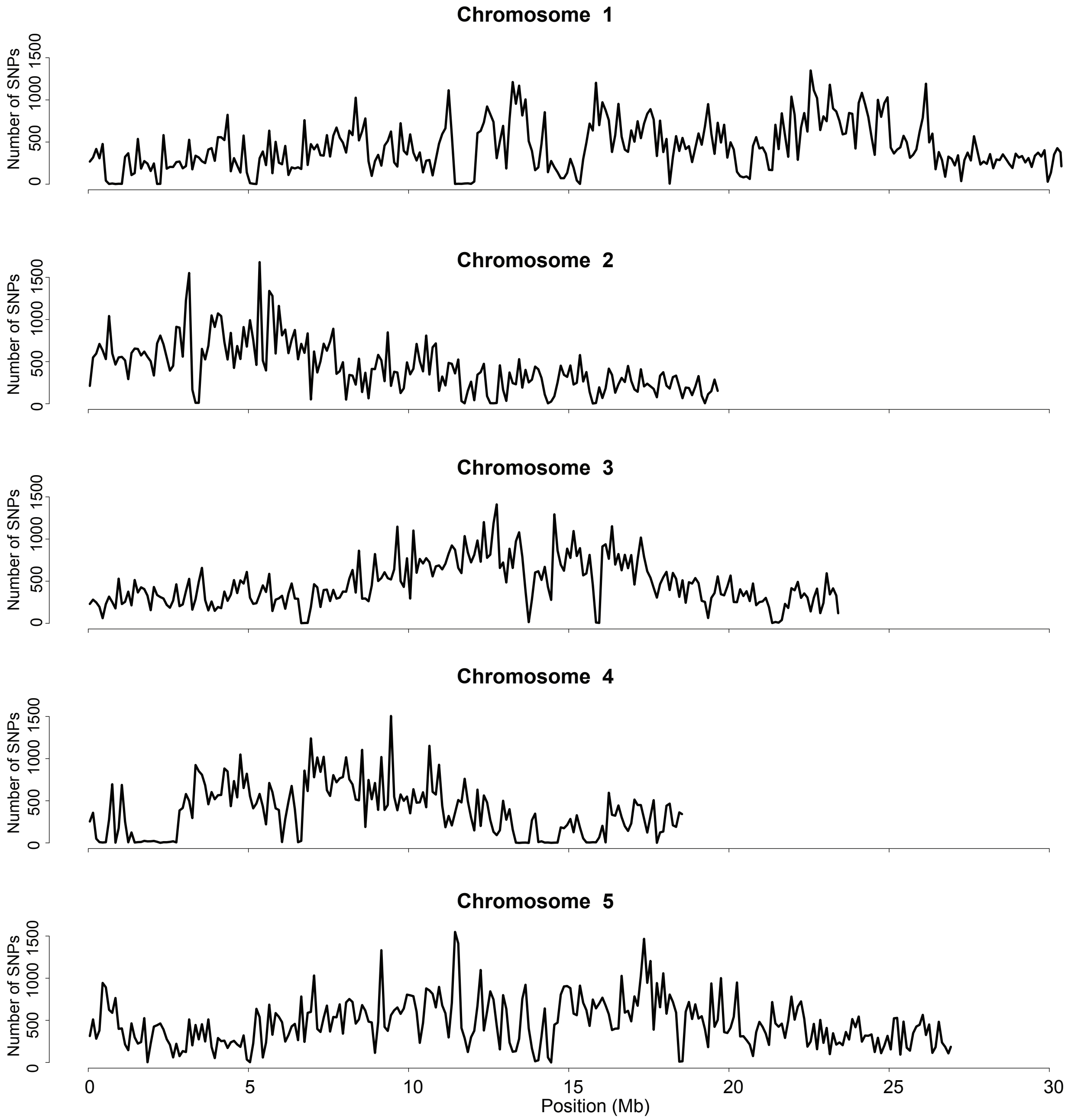

Figure S11 SNP density between Col-0 and Ws-2. The post-filtering SNP density (see Figure S4 for filtering parameters) using a sliding window of 100 kb is shown for each of the five *A. thaliana* chromosomes.

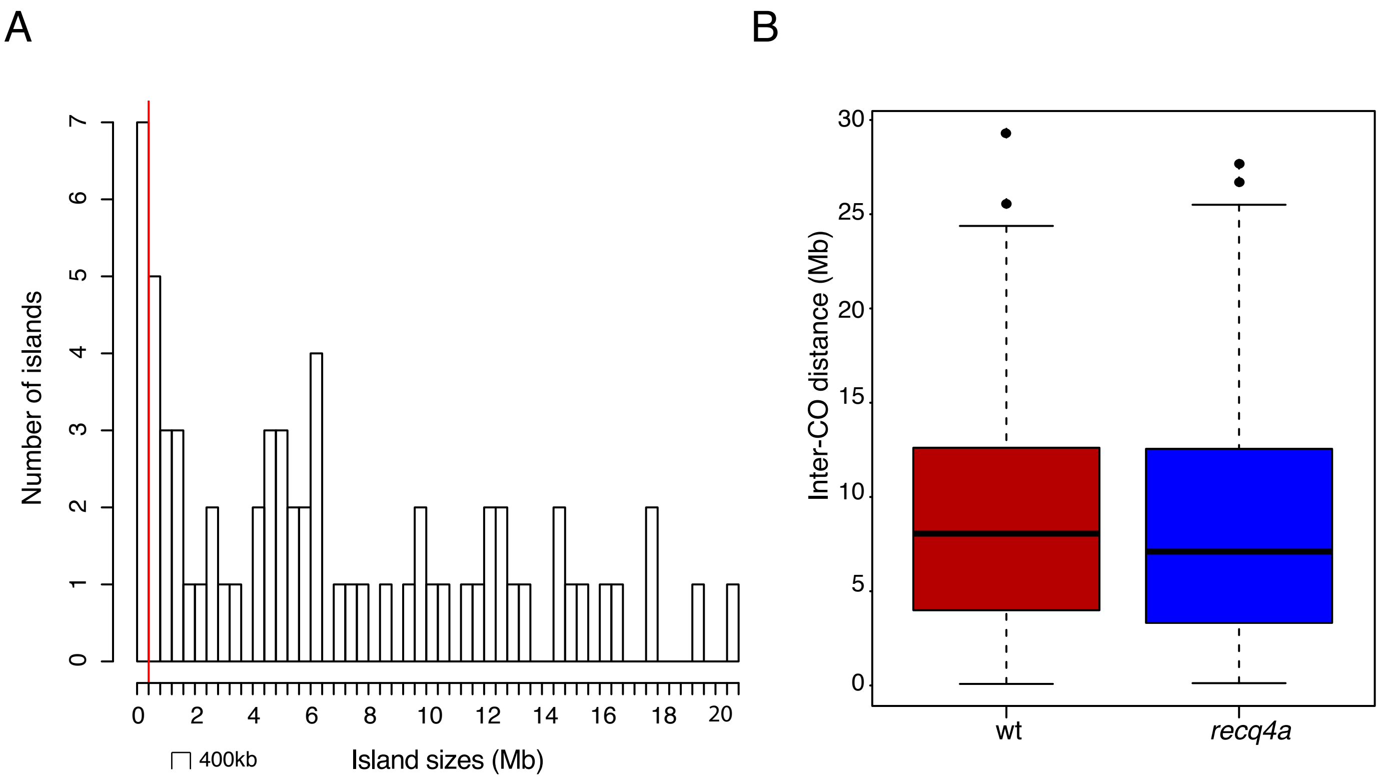

Figure S12 "Island" errors and double COs. TIGER-generated reconstructions of experimental recombinant individuals produced a type of error where a small genotype block was embedded in a larger block of a different genotype. A) Histogram depicting the frequency of the lengths of these small genotype "islands" is shown. Some of these islands are errors, others might represent real closely-spaced double COs. The red line indicates the chosen threshold for distinguishing between island errors and true double COs. B) Box plots showing the inter-CO distances for all true double COs in the wt compared with the mutant population.

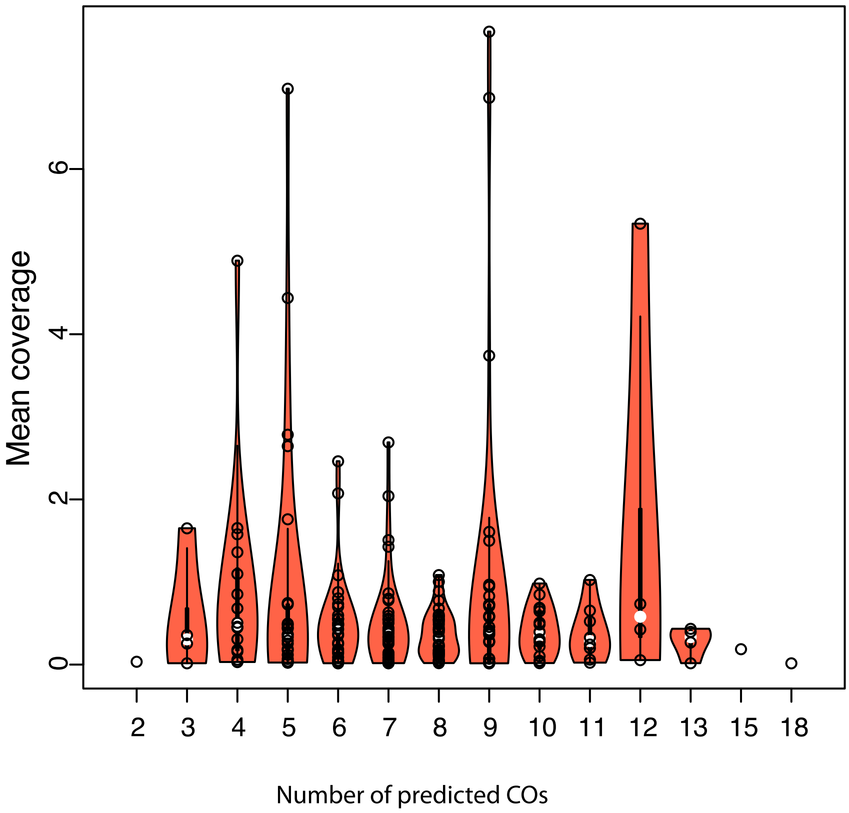

Figure S13 The effect of coverage on CO prediction using TIGER. The density curve for probabilities (orange) is indicated for each number of predicted COs compared to the coverage rate.

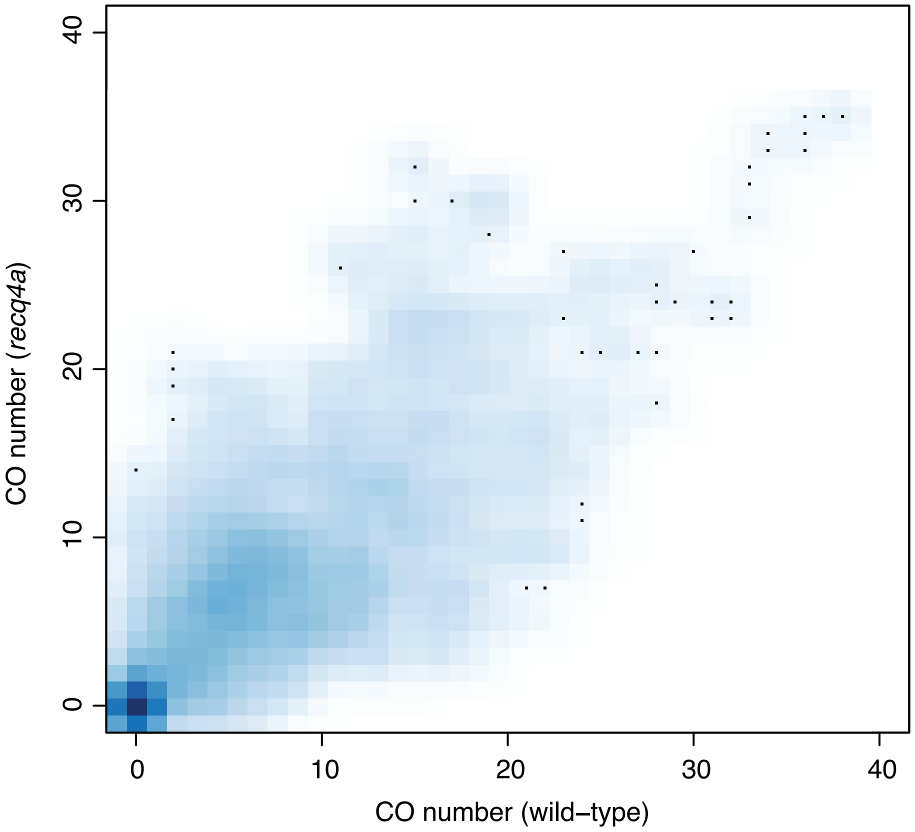

Figure S14 Correlation between CO distributions throughout the genome between wild-type and *recq4a* F_2 populations. Frequencies of 800-kb windows with the given numbers of COs are shown using a color scale from light (lowest) to dark (highest) blue.

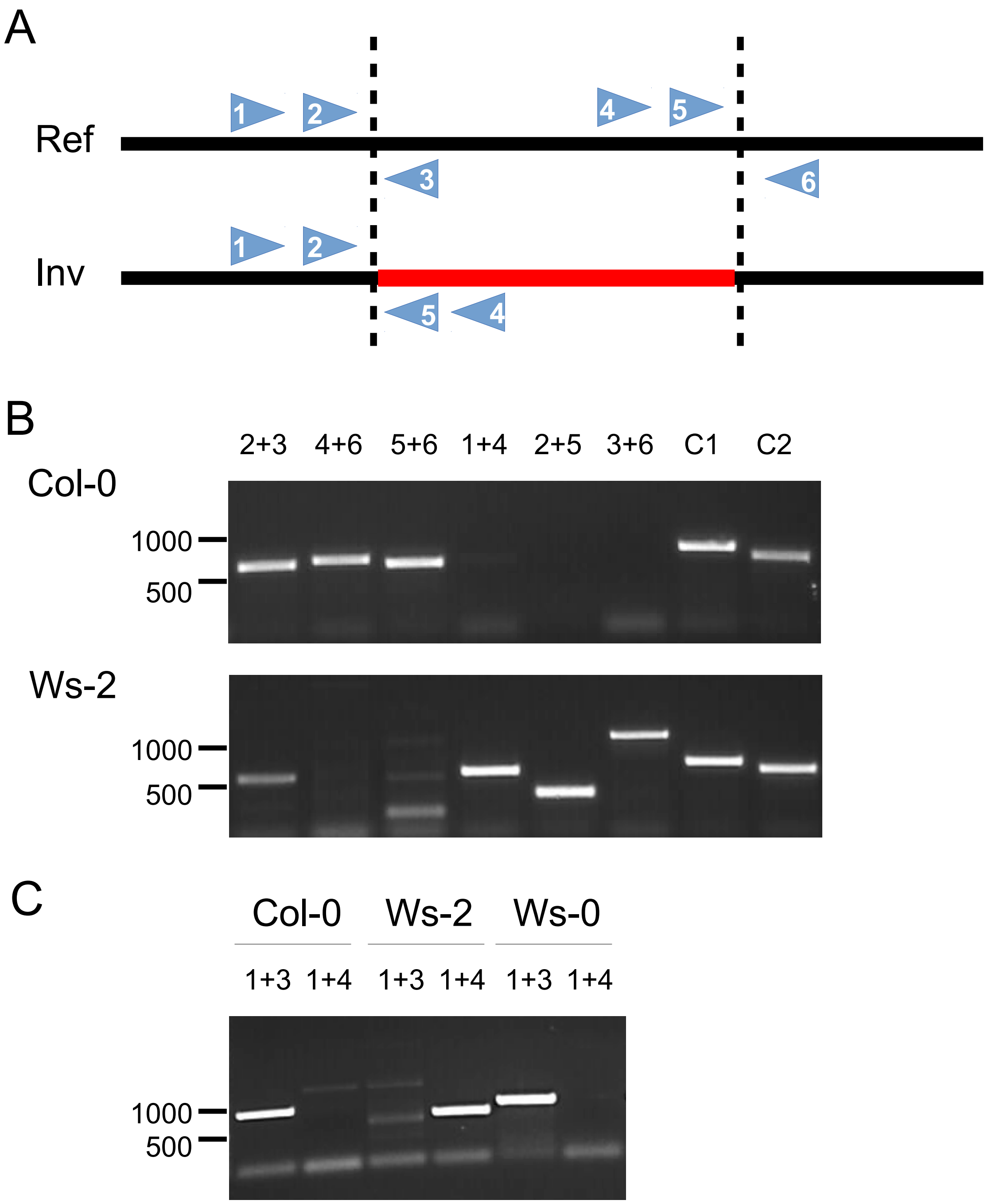

Figure S15 PCR amplification around predicted inversion breakpoints. (A) Schematic diagram showing the relative locations and orientations of primers around the predicted breakpoint positions (dashed vertical lines) at base positions 7139542 and 8914936 on Chr 4 (not drawn to scale). B) PCR bands amplified using the indicated combinations of the primers shown in A. "C1" is a control amplicon from Chr 5 (*MAF4*) and "C2" is a control amplicon from Chr 1. C) PCR bands amplified using the indicated combinations of the primers shown in A for Col-0, Ws-2 and Ws-0. The faint band visible for Ws-2 in the lane for primers 2+3 in B) is likely an off-target amplicon, as this primer set amplified bands of different sizes from Ws-2 DNA in replicate experiments at different annealing temperatures (data not shown).

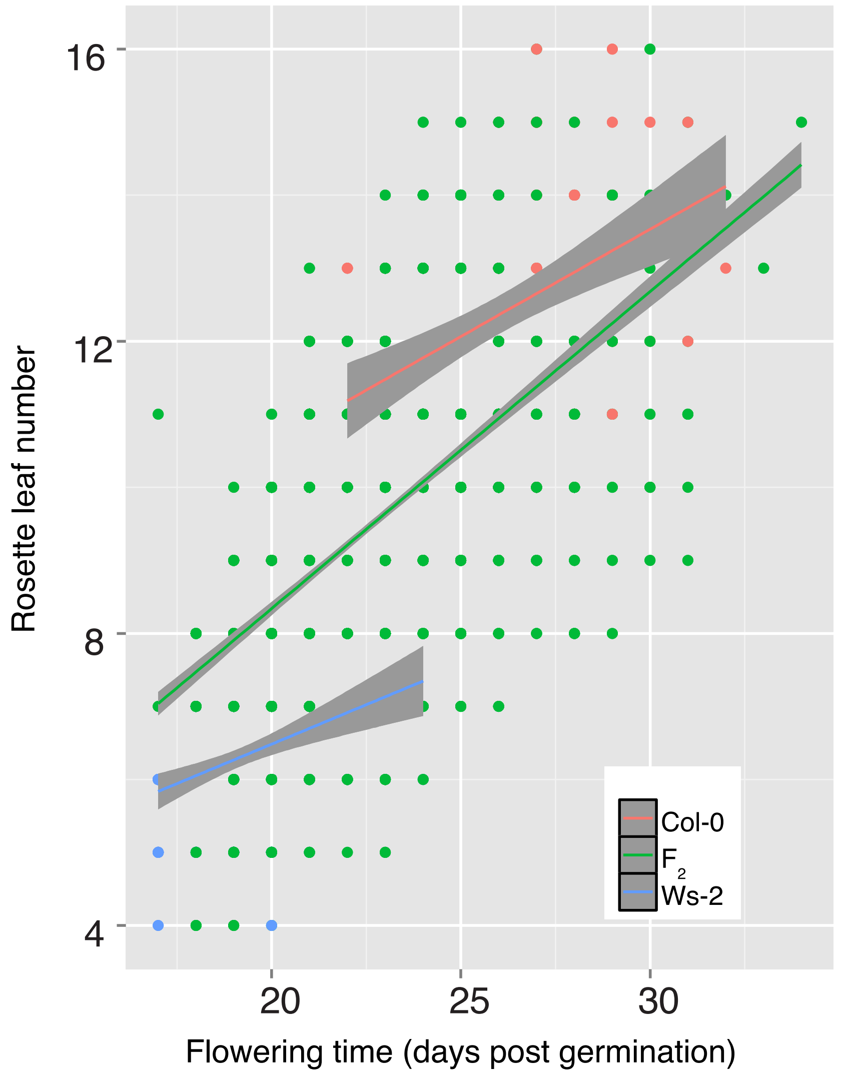

Figure S16 Correlation between the number of days to flower and the number of rosette leaves at flowering. R^2 = 0.18, 0.3, and 0.09 for Col-0, F, and Ws-2, respectively. N=129, 2185, and 202 for Col-0, F_2, and Ws-2, respectively. Equation of Col line: y = 0.29x + 4.7 R^2 = 0.18. Equation of F_2 line: y = 0.43x - 0.35 R^2 = 0.3. Equation of Ws-2 line: y = 0.22x + 2.3 R^2 = 0.09.

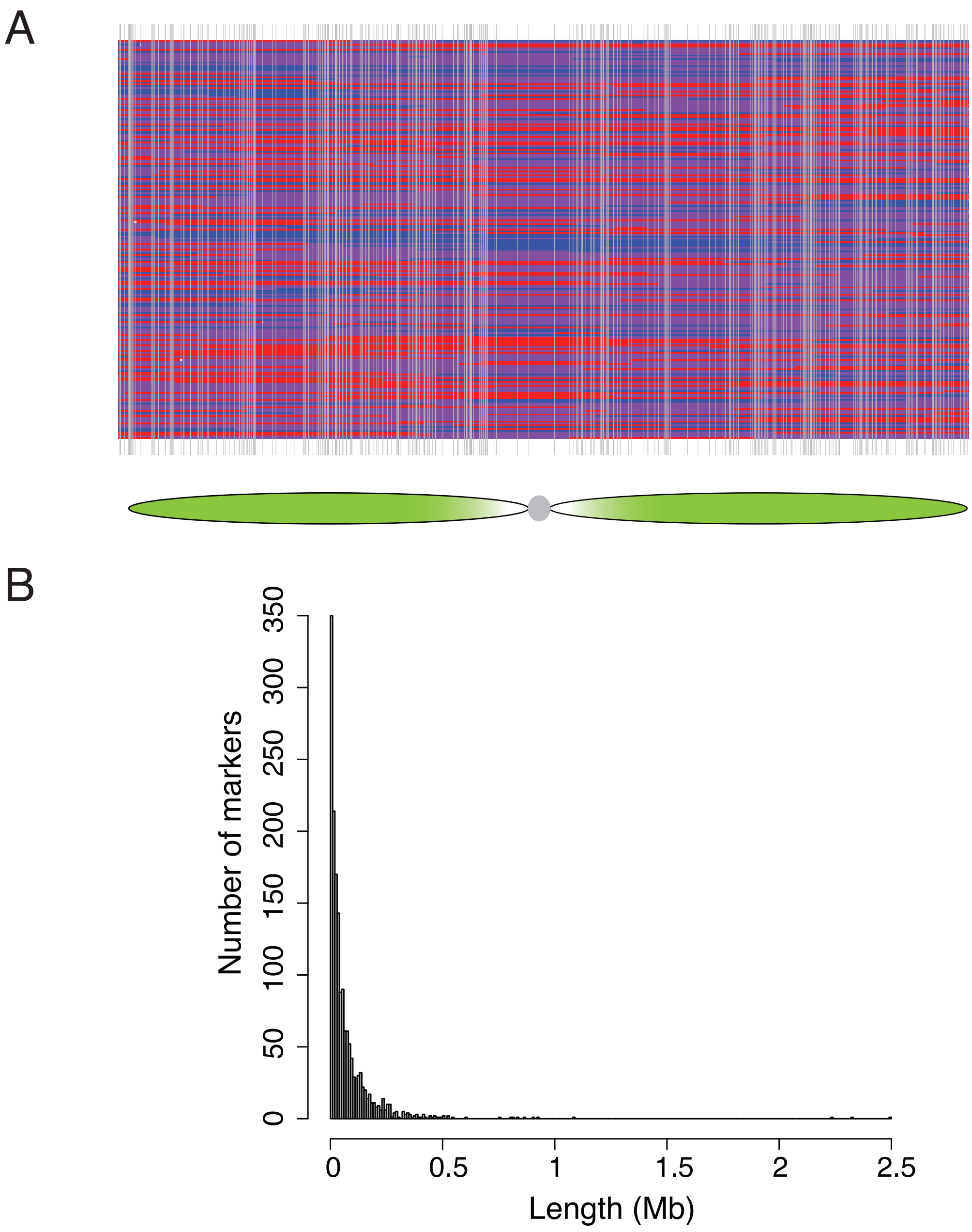

-
-

Figure S17 Recombination blocks used as markers for QTL analysis. A) A graphical representation of the recombination block that were usaed as markers for QTL mapping of flowering time in wt and *recq4a.* Graphical reconstructions of the genotypes along chromosome 1 are shown. Each horizontal line represents a single individual. Red indicates homozygous for Col-0, blue depicts homozygous for Ws-2 and heterozygous regions are in purple. The thin grey lines indicate the positions where at least one individual had a CO. SNPs in the regions between lines were used as markers for QTL analysis. B) Length distributions of the recombination

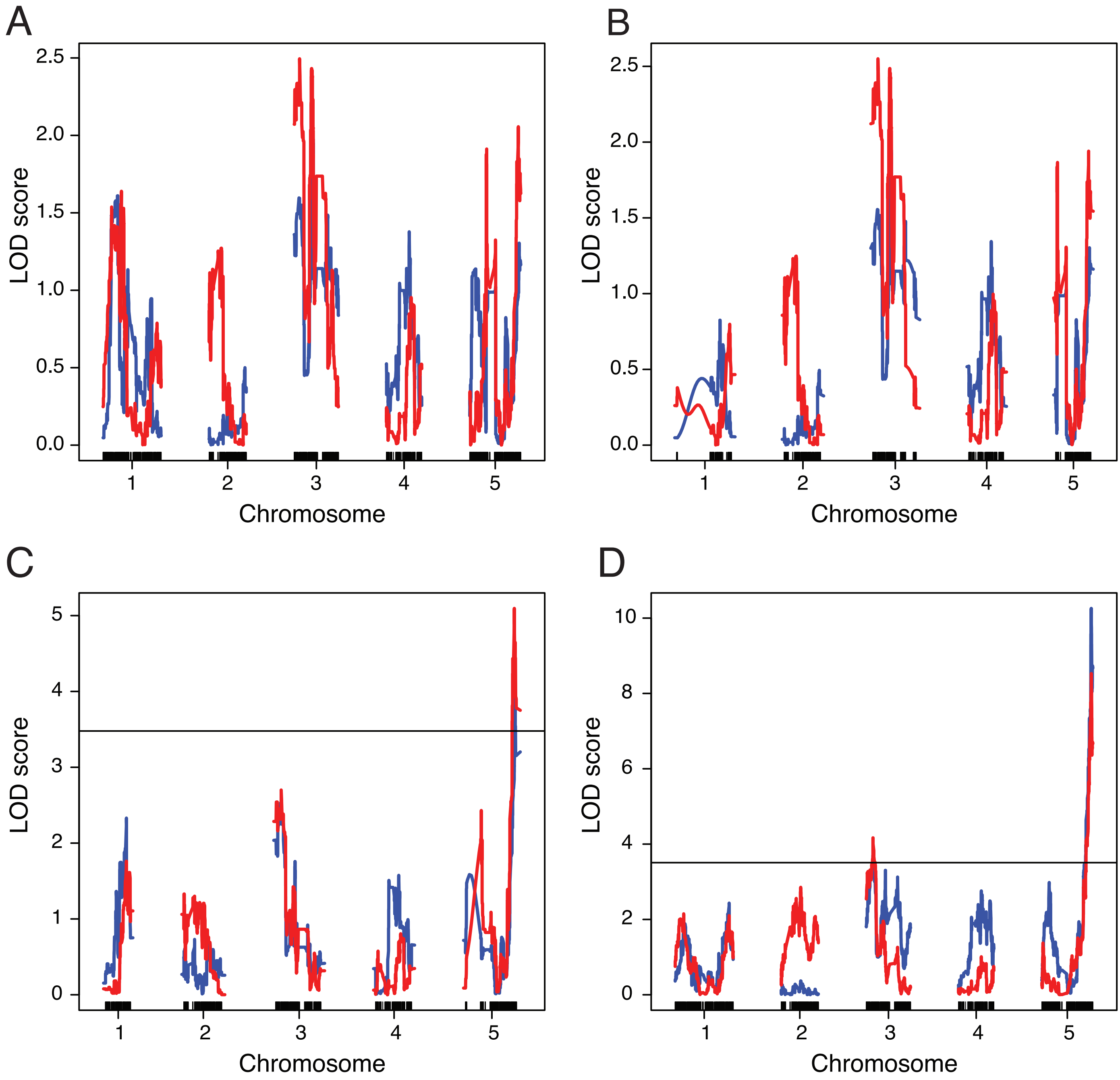

Figure S18 Additional plots showing the results of QTL analyses for flowering time. QTL analyses of the number of days to flowering (blue) and rosette leaf number (red) for all individuals of the wt population (A), all individuals of the wild-type population after dropping distorted markers (B), the wild-typet population with selected individuals and distorted markers dropped (C), and the combined wild-type and *recq4a* populations (D). Vertical ticks along the x-axis indicate the positions of the SNP markers genotyped. Horizontal lines indicate the position of the significance threshold for the population shown ($p < 0.05$ for 1000 permutations). Absence of a horizontal line indicates that no LOD scores passed the threshold.

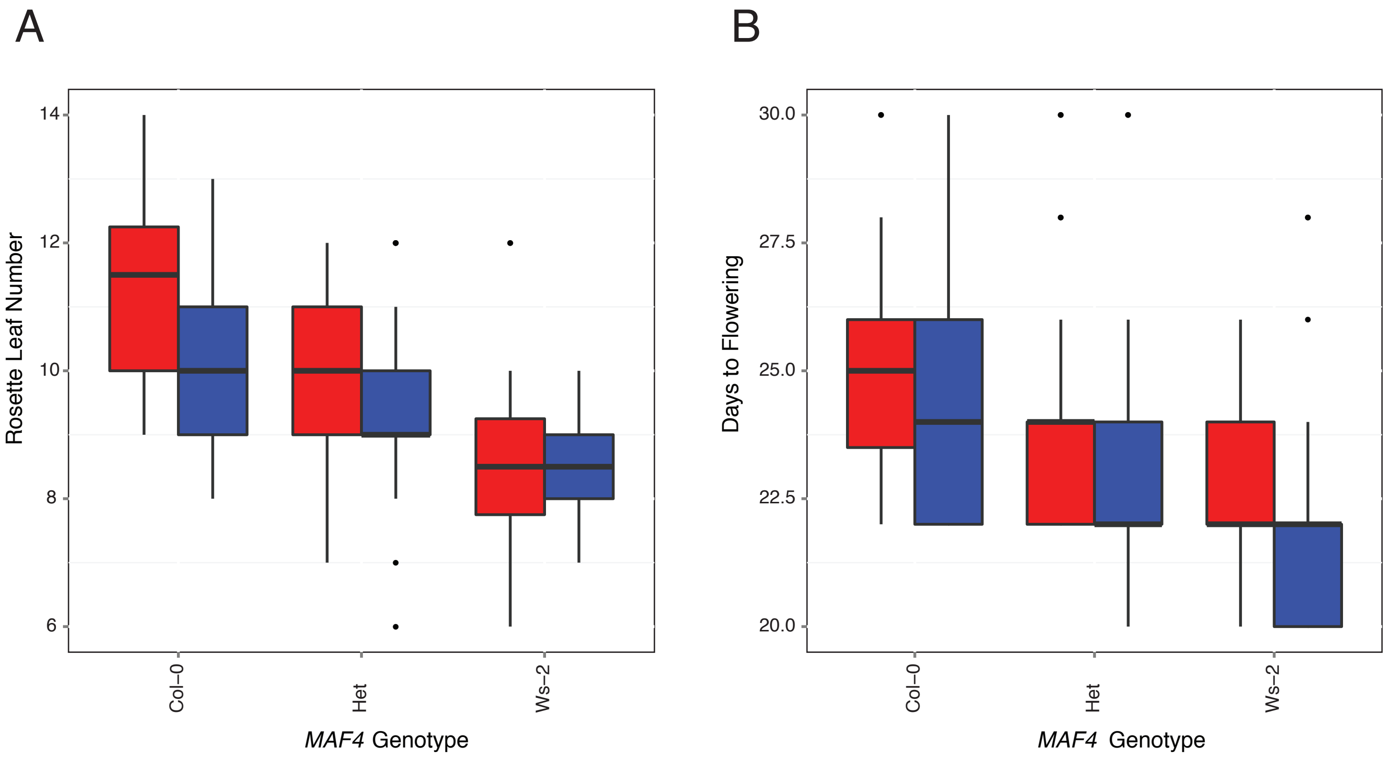

Figure S19 Association between flowering time and *MAF4* genotype. Box plots showing the rosette leaf number (A) and the number of days until flowering (B) for wt and *recq4a* F2 individuals categorized by *MAF4* genotype. Phenotypes were scored when the inflorescence shoot reached the height of 1 cm.
