# Supplementary material for: Rapid and Inexpensive Whole-Genome Genotyping-by-Sequencing for Crossover Localization and Fine-Scale Genetic Mapping
Source: G3 (Bethesda). 2015 Jan 13;5(3):385–98. doi: 10.1534/g3.114.016501 (PMC4349092; doi:10.1534/g3.114.016501)
Supplement: Supporting Information [file supp_g3.114.016501_TableS1.pdf]

Table S1 Adapter index sequences.

Index Number	Sequence	Index Number	Sequence	Index Number	Sequence	Index Number	Sequence
1	ACGTAGCT	25	AGATCGCA	49	GATAGACA	73	AATGTTGC
2	TACGTCAG	26	AGCAGGAA	50	GCCACATA	74	ACACGACC
3	CGTCGATA	27	AGTCACTA	51	GCGAGTAA	75	ACAGATTC
4	GTAACTGC	28	ATCCTGTA	52	GCTAACGA	76	AGATGTAC
5	AACGTGAT	29	ATTGAGGA	53	GCTCGGTA	77	AGCACCTC
6	CGCTGATC	30	CAACCACA	54	GGAGAACA	78	AGCCATGC
7	CAGATCTG	31	CAAGACTA	55	GGTGCGAA	79	AGGCTAAC
8	ATGCCTAA	32	CAATGGAA	56	GTACGCAA	80	ATAGCGAC
9	CTGTAGCC	33	CACTTCGA	57	GTCGTAGA	81	ATCATTCC
10	AGTACAAG	34	CAGCGTTA	58	GTCTGTCA	82	ATTGGCTC
11	CATCAAGT	35	CATACCAA	59	GTGTTCTA	83	CAAGGAGC
12	AGTGGTCA	36	CCAGTTCA	60	TAGGATGA	84	CACCTTAC
13	AACAACCA	37	CCGAAGTA	61	TATCAGCA	85	CCATCCTC
14	AACCGAGA	38	CCGTGAGA	62	TCCGTCTA	86	CCGACAAC
15	AACGCTTA	39	CCTCCTGA	63	TCTTCACA	87	CCTAATCC
16	AAGACGGA	40	CGAACTTA	64	TGAAGAGA	88	CCTCTATC
17	AAGGTACA	41	CGACTGGA	65	TGGAACAA	89	CGACACAC
18	ACACAGAA	42	CGCATACA	66	TGGCTTCA	90	CGGATTGC
19	ACAGCAGA	43	CTCAATGA	67	TGGTGGTA	91	CTAAGGTC
20	ACCTCCAA	44	CTGAGCCA	68	TTCACGCA	92	GAACAGGC
21	ACGCTCGA	45	CTGGCATA	69	AACTCACC	93	GACAGTGC
22	ACGTATCA	46	GAATCTGA	70	AAGAGATC	94	GAGTTAGC
23	ACTATGCA	47	GACTAGTA	71	AAGGACAC	95	GATGAATC
24	AGAGTCAA	48	GAGCTGAA	72	AATCCGTC	96	GCCAAGAC

Sequences 1-4 were designed in house. Sequences 5 – 96 were taken from Mamanova et al. 2010.
