# Supplementary material for: Rapid and Inexpensive Whole-Genome Genotyping-by-Sequencing for Crossover Localization and Fine-Scale Genetic Mapping
Source: G3 (Bethesda). 2015 Jan 13;5(3):385–98. doi: 10.1534/g3.114.016501 (PMC4349092; doi:10.1534/g3.114.016501)
Supplement: Supporting Information [file supp_g3.114.016501_TableS2.pdf]

Table S2 Primers used for PCR amplification and Sanger sequencing reactions

	Sequence (5' to 3')	CAPS enzyme	Allele cut
MAF Genotyping			
forward	ACTTCGTTCTTCACTACCGGT	StyI	Ws-2
reverse	GTTCTCCTCTCTCAGCAGCT	StyI	Ws-2
Ws-2 Inversion			
Primer 1	TGGCCTGCGTCTAAACAATG		
Primer 2	CCATCAATCTCAATTCACAAGGG		
Primer 3	ATGACCAGACCACTTTCCGG		
Primer 4	GACGTCGAGGCTGATAATTGA		
Primer 5	CGGGAAAGGACTGCTGAACT		
Primer 6	AACAGCAAAGCCACCATCAC		
C2 forward	TGGTCCGGTGGTGATTTACA		
C2 reverse	CGGCTTCTCGAGTTACTCTT		
Breakpoint resolution			
ID1 forward	GATGTTACCGTGGGTCGATT		
ID1 reverse	GTCTTCAACTTCCGGCGATA		
ID2 forward	CTTTGGCCGATAGACAGGAG		
ID2 reverse	TCAGCCACCACCACTACAAA		
ID3 forward	GCAGAAGATTCCGAGAGTGG		
ID3 reverse	TGGTCTTGGTCCGAAAATGT		
ID4 forward	TTCCCAATTTCGATCCAGAG		
ID4 reverse	CAGGCGGAGGAGTGTAAGAG		
ID5 forward	CCCGCCAAAATAAACAAAGA		
ID5 reverse	CACGAAGTTTCTCGGCTTTC		
ID6 forward	TGAACAGACAATTTGCTCCAA		
ID6 reverse	AGGCCAACGTTTAGGAGGAT		
ID7 forward	TTCCCTGCATCCTAGTCCTG		
ID7 reverse	AAAAGGAATGGCACACGTTC		
ID8 forward	CCGTCAATTTGAGCAGGAAT		
ID8 reverse	TTCTACGCACACCAGGGATT		
ID9 forward	GCGTAATCATCACTCGCTCA		
ID9 reverse	TGCAAACACGGAAGACAGTT		
ID10 forward	GCCATGCGTACCTGAAAAGT		
ID10 reverse	ATTGGCATCACGAGGAAAGT		
ID11 forward	CAATTAACAAGGCCGAGTAAAGA		
ID11 reverse	GGAATCTCCCTTGGTCCCTA		
