# Supplementary material for: Rapid and Inexpensive Whole-Genome Genotyping-by-Sequencing for Crossover Localization and Fine-Scale Genetic Mapping
Source: G3 (Bethesda). 2015 Jan 13;5(3):385–98. doi: 10.1534/g3.114.016501 (PMC4349092; doi:10.1534/g3.114.016501)
Supplement: Supporting Information [file supp_g3.114.016501_TableS3.pdf]

Table S3 Price comparison for individual components and reagents needed for paired-end library preparation

Our Method

	Manufacturer	Part number	Price per package	Samples per package	Price per sample
**DNA fragmentation**					
dsDNA Shearase™*	Zymo Research	E2018-200	396	400	0.99
**A-tailing**					
Klenow exo-	New England Biolabs	M0212L	236	400	0.59
dATP	New England Biolabs	N0440S	48	5000	0.01
**Adapter Ligation**					
Custom adapter oligos	Sigma-Aldrich	NA	3840	576000	0.01
P2 adapter oligos	Sigma-Aldrich	NA	40	6000	0.01
Quick Ligation Kit™	New England Biolabs	M2200L	388	300	1.29
NEB Buffer 2	New England Biolabs	B7002S	18	384	0.05
**PCR Enrichment**					
Phusion Mastermix	New England Biolabs	M0531S	170	9600	0.02
Primer oligos	Sigma-Aldrich	NA	47	28800000	0.000002
**TOTAL for Reagents**					**2.96**
**Clean-ups**					
AmpPure XP Magnetic Beads	Beckman-Coulter	A63881	1126	508	2.22
**TOTAL for Library prep**					**5.18**
**Validation**					
Qubit DNA quantification reagents	Life Technologies	Q32851	79	9600	0.01
Bioanlayzer reagents	Agilent Technologies	5067-1505	349	28800	0.01
**TOTAL for Preparation/Validation**					**5.20**
**Pre-Library Prep Quantification**					
Qubit DNA quantification reagents	Life Technologies	Q32851	79	100	0.79
**TOTAL for all**					**5.99**

Illumina Tru-Seq Nano

	Manufacturer	Part number	Price per package	Samples per package	Price per sample
**DNA Fragmentation**					
	Covaris	520045	125	25	5
**Library Prep**					
TruSeq Nano	Illumina	FC-121-4003	2880	96	30
**TOTAL for Library prep**					**35**
**Validation**					
Qubit DNA quantification reagents	Life Technologies	Q32851	79	9600	0.01
Bioanlayzer reagents	Agilent Technologies	5067-1505	349	28800	0.01
**TOTAL for Preparation/Validation**					**35.02**
**Pre-Library prep Quantification**					
Qubit DNA quantification reagents	Life Technologies	Q32851	79	100	0.79
**TOTAL for all**					**35.81**

Prices are based on the list price for the US market (in US dollars). The reagents needed for library quantification for normalization purposes, plastic consumables such as 96-well PCR plates, and general laboratory reagents are not included, as they not included in the Illumina TruSeq kit.
