# Supplementary material for: Rapid and Inexpensive Whole-Genome Genotyping-by-Sequencing for Crossover Localization and Fine-Scale Genetic Mapping
Source: G3 (Bethesda). 2015 Jan 13;5(3):385–98. doi: 10.1534/g3.114.016501 (PMC4349092; doi:10.1534/g3.114.016501)
Supplement: Supporting Information [file supp_g3.114.016501_TableS4.pdf]

Table S4. Genotype frequencies in wt and *recq4a* F_2 populations

	Col-0	Ws-2	Het
wt	28.0%	21.8%	50.2%
recq4a	23.8%	25.6%	50.7%
