# Supplementary material for: Rapid and Inexpensive Whole-Genome Genotyping-by-Sequencing for Crossover Localization and Fine-Scale Genetic Mapping
Source: G3 (Bethesda). 2015 Jan 13;5(3):385–98. doi: 10.1534/g3.114.016501 (PMC4349092; doi:10.1534/g3.114.016501)
Supplement: Supporting Information [file supp_g3.114.016501_TableS5.pdf]

Table S5 Summary of flowering time statistics for parental and F_2 populations

Background	Genotype	N	Mean Days to Flowering (± sem)	Mean Rosette Leaf Number (± sem)
Col-0	wt	64	27.19 (± 0.18)	13.34 (± 0.19)
Col-0	*recq4a*	65	24.92 (± 0.32)	11.4 (± 0.14)
F_2	wt	1133	23.07 (± 0.07)	9.66 (± 0.05)
F_2	*recq4a*	1052	21.95 (± 0.07)	9.23 (± 0.06)
Ws-2	wt	66	19.65(± 0.16)	6.72 (± 0.14)
Ws-2	*recq4a*	136	18.96 (± 0.11)	6.1 (± 0.07)

Phenotypes were scored when the inflorescence shoot reached the height of 1 cm. Abbreviations used: SD, standard deviation; sem, standard error of the mean.
