# Supplementary material for: Rapid and Inexpensive Whole-Genome Genotyping-by-Sequencing for Crossover Localization and Fine-Scale Genetic Mapping
Source: G3 (Bethesda). 2015 Jan 13;5(3):385–98. doi: 10.1534/g3.114.016501 (PMC4349092; doi:10.1534/g3.114.016501)
Supplement: Supporting Information [file supp_g3.114.016501_TableS6.pdf]

Table S6 Additional statistics for QTL analyses of flowering time

	Position (Mb)	LOD	Variance explained (%)
wt			
Rosette Leaf Number	25.55	5.09	20.7
Days to Flower	25.99	3.92	16.4
recq4a			
Rosette Leaf Number	26.02	8.73	24.6
Days to Flower	25.86	9.55	23.7
Combined			
Rosette Leaf Number	26.02	8.52	16.7
Days to Flower	25.99	10.25	19.5
