# Supplementary material for: Rapid and Inexpensive Whole-Genome Genotyping-by-Sequencing for Crossover Localization and Fine-Scale Genetic Mapping
Source: G3 (Bethesda). 2015 Jan 13;5(3):385–98. doi: 10.1534/g3.114.016501 (PMC4349092; doi:10.1534/g3.114.016501)
Supplement: Supporting Information [file supp_g3.114.016501_TableS7.pdf]

Table S7 Flowering time as a function of *MAF4* and *RECQ4A* genotypes

	DF	Sum of Squares	Mean Squares	F value	P-value
**Days to Flowering**					
MAF4 Genotype	2	78.7	39.4	8.6	2.8e-4
RECQ4A genotype	1	16.3	16.3	3.6	0.06
RECQ4A x *MAF4* Genotype	2	0.8	0.4	0.09	0.92
**Rosette Leaf Number**					
RECQ4A genotype	2	91.7	45.8	26.5	9e-11
MAF4 Genotype	1	19.5	19.5	11.3	1e-3
RECQ4A x *MAF4* Genotype	2	6.8	3.4	1.95	0.15

Flowering time phenotypes were scored among F_2 individuals from the wt and *recq4a* populations that had been genotyped at the *MAF4* locus. Shown are the results of an analysis of variance (ANOVA). See Table S5 for summary statistics of the phenotypic data.
