# Supplementary figures and images for: Rapid and Inexpensive Whole-Genome Genotyping-by-Sequencing for Crossover Localization and Fine-Scale Genetic Mapping
Source: G3 (Bethesda). 2015 Jan 13;5(3):385–98. doi: 10.1534/g3.114.016501 (PMC4349092; doi:10.1534/g3.114.016501)

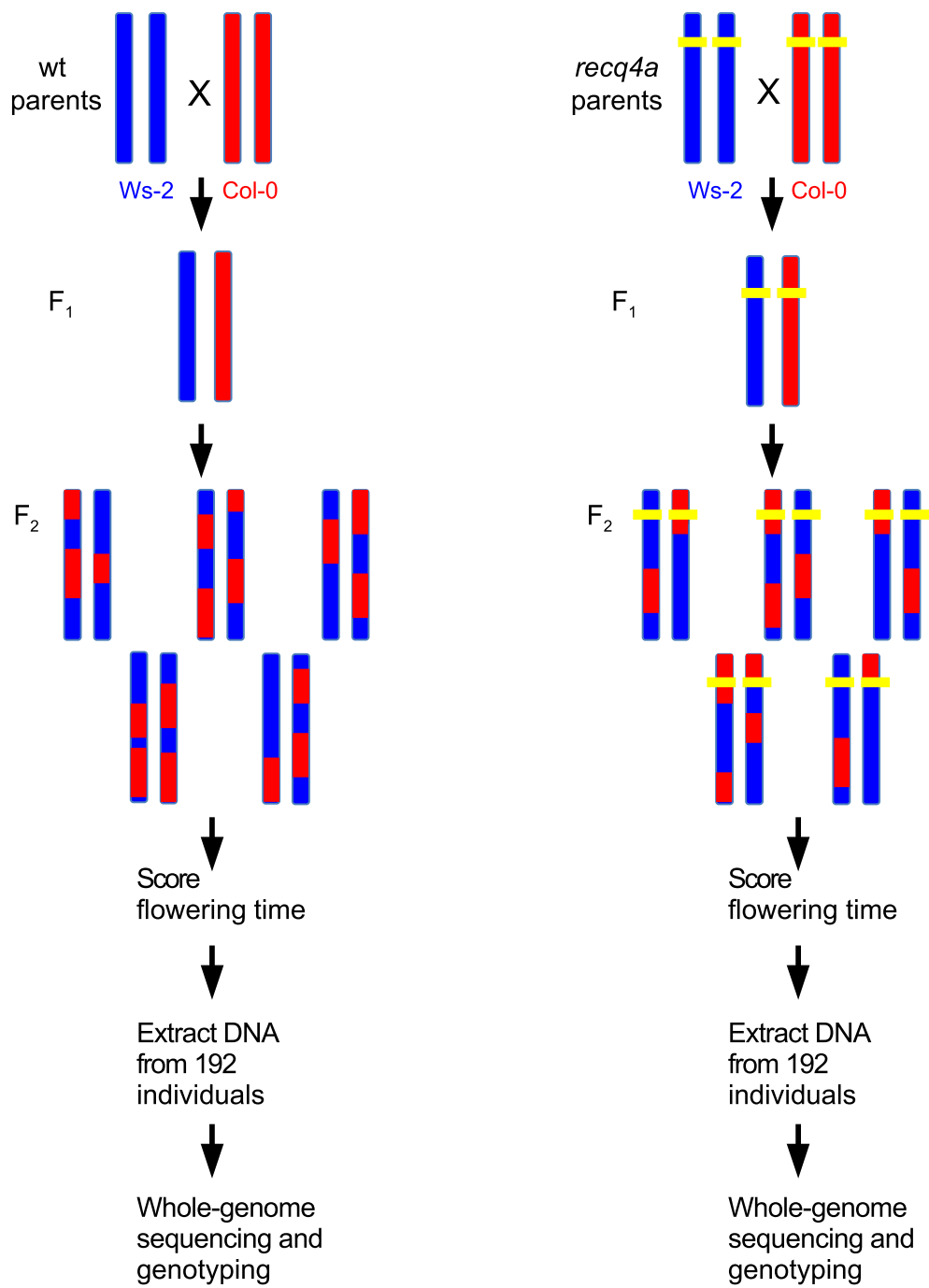

**Figure S1.** Crossing scheme and workflow for mapping flowering time QTL.

Supplement: Supporting Information [file supp_g3.114.016501_FigureS1.pdf]

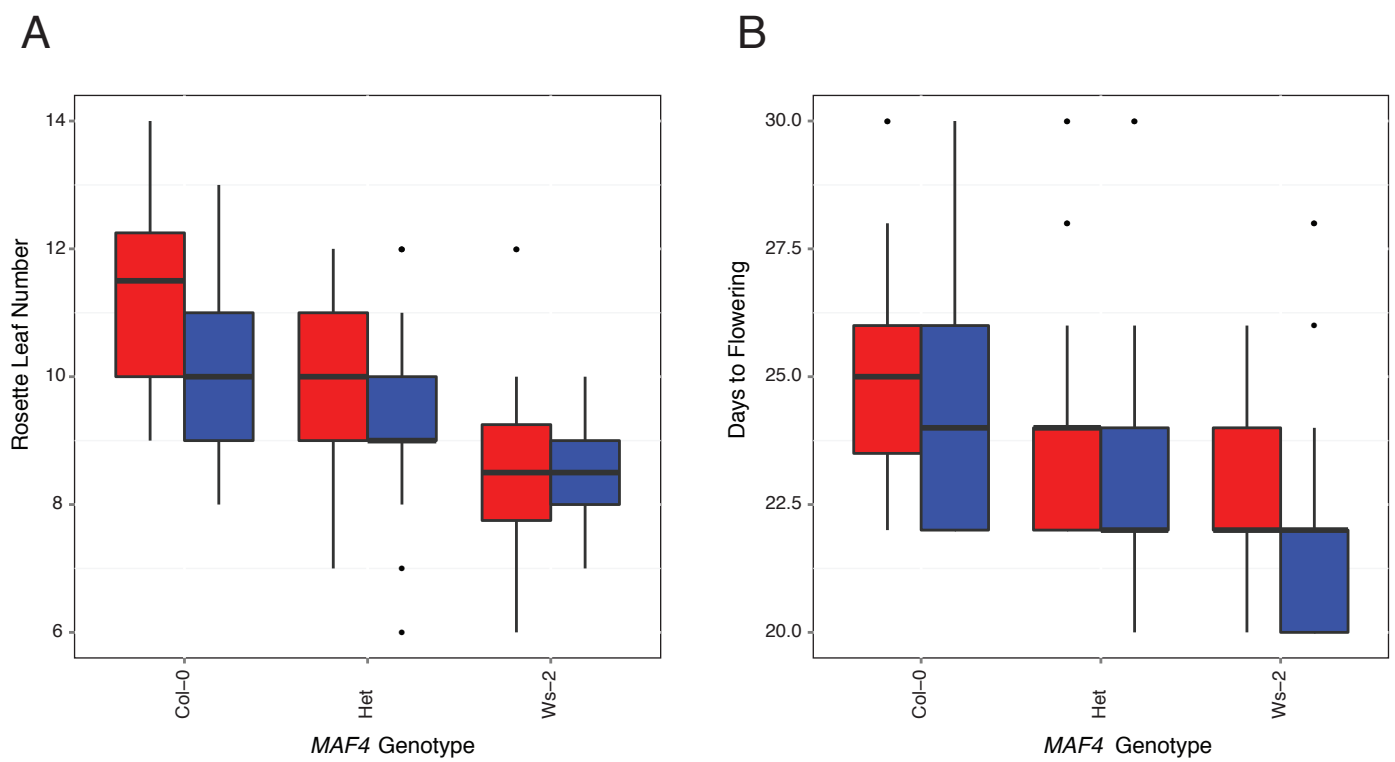

Supplement: Supporting Information [file supp_g3.114.016501_FigureS19.pdf]
