# Supplementary material for: Rapid and Inexpensive Whole-Genome Genotyping-by-Sequencing for Crossover Localization and Fine-Scale Genetic Mapping
Source: G3 (Bethesda). 2015 Jan 13;5(3):385–98. doi: 10.1534/g3.114.016501 (PMC4349092; doi:10.1534/g3.114.016501)
Supplement: Supporting Information [file supp_g3.114.016501_FigureS2.pdf]

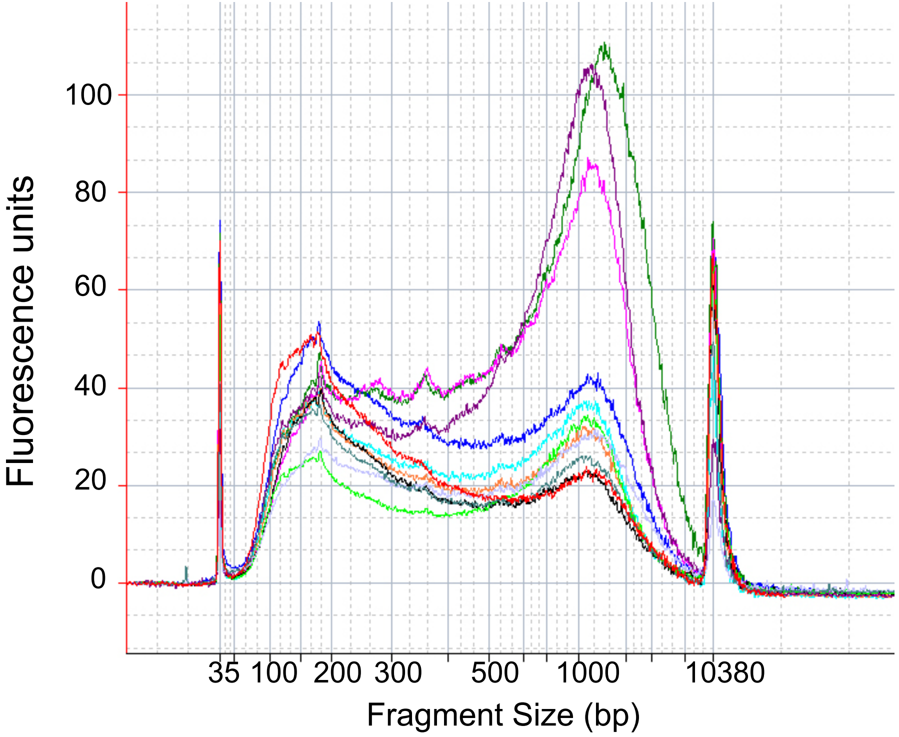

Figure S2 Fragment size distribution of Shearase™-digested Arabidopsis thaliana DNA. Bioanalyzer traces of 11 DNA samples from one 96-plex library prep after digestion with the Shearase™ enzyme are shown.
