# Supplementary material for: Rapid and Inexpensive Whole-Genome Genotyping-by-Sequencing for Crossover Localization and Fine-Scale Genetic Mapping
Source: G3 (Bethesda). 2015 Jan 13;5(3):385–98. doi: 10.1534/g3.114.016501 (PMC4349092; doi:10.1534/g3.114.016501)
Supplement: Supporting Information [file supp_g3.114.016501_FigureS3.pdf]

A

P1

INDEX1

5' ACACTCTTTCCCTACACGACGCTCTTCCGATCT***ACGTAGCT*****T* (sense)

5'P-***AGCTACGT***AGATCGGAAGAGCGTCGTGTAGGGAAAGAGTG*T (antisense)

P2

5' P-GATCGGAAGAGCGGTTCAGCAGGAATGCCGA*G (sense)

5' CTCGGCATTCCTGCTGAACCGCTCTTCCGATC*T (antisense)

B

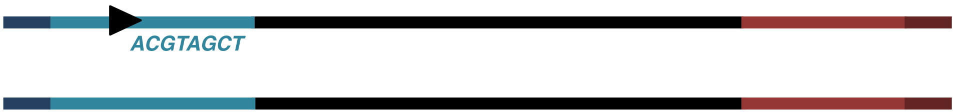

Figure S3 Design of custom adapters used for multiplexing. A) Oligo sequences for the indexing adapters. The index is added to the 3' end of the Illumina P1 adapter. An example index sequence (in italics) is shown. See Table S1 for all 96 index sequences. "P" is used to indicate a 5' phosphate addition. The * shows the position of a phosphorothioate bond. Oligo sequences for the universal Illumina P2 adapter are also shown. B) Schematic diagram showing adapters ligated to DNA fragments. The arrow indicates the direction of sequencing using the Illumina primer for the first read. The first 8 bases of sequence for read 1 are the index sequence.
