# Supplementary material for: Rapid and Inexpensive Whole-Genome Genotyping-by-Sequencing for Crossover Localization and Fine-Scale Genetic Mapping
Source: G3 (Bethesda). 2015 Jan 13;5(3):385–98. doi: 10.1534/g3.114.016501 (PMC4349092; doi:10.1534/g3.114.016501)
Supplement: Supporting Information [file supp_g3.114.016501_FigureS4.pdf]

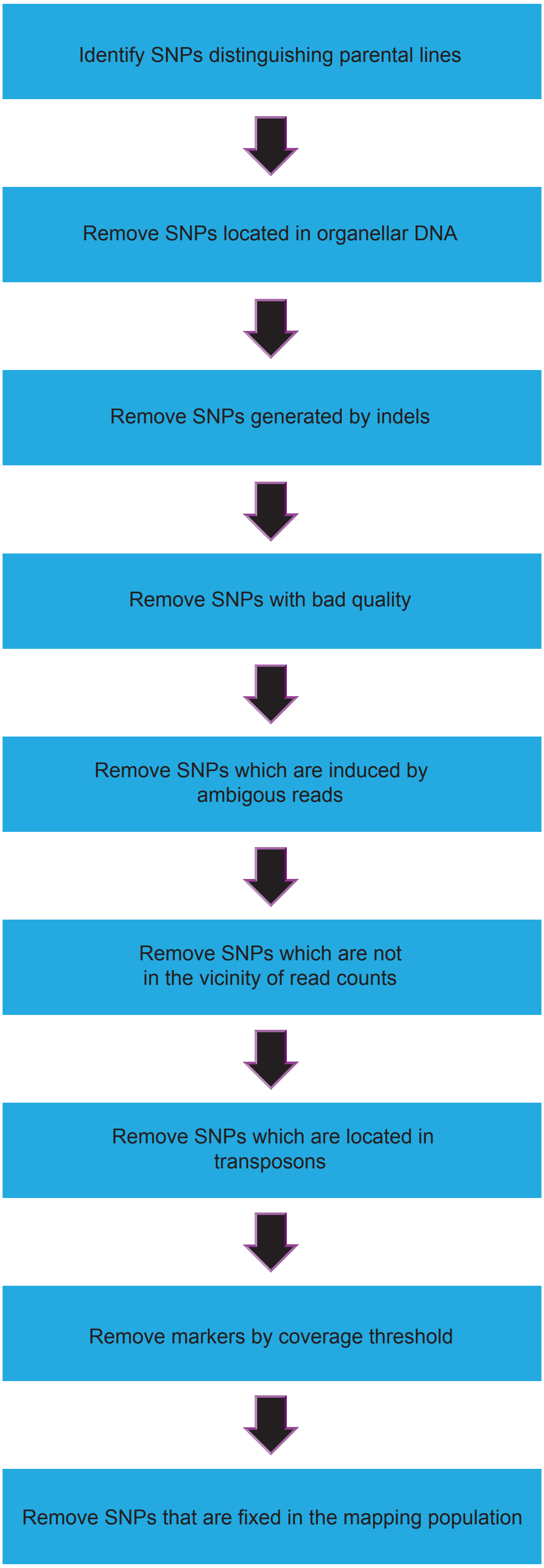

Figure S4 Ws-2 marker filtering workflow. Stepwise representation of the marker filtering pipeline used to select SNPs for downstream implementation in TIGER.
