# Supplementary material for: Rapid and Inexpensive Whole-Genome Genotyping-by-Sequencing for Crossover Localization and Fine-Scale Genetic Mapping
Source: G3 (Bethesda). 2015 Jan 13;5(3):385–98. doi: 10.1534/g3.114.016501 (PMC4349092; doi:10.1534/g3.114.016501)
Supplement: Supporting Information [file supp_g3.114.016501_FigureS6.pdf]

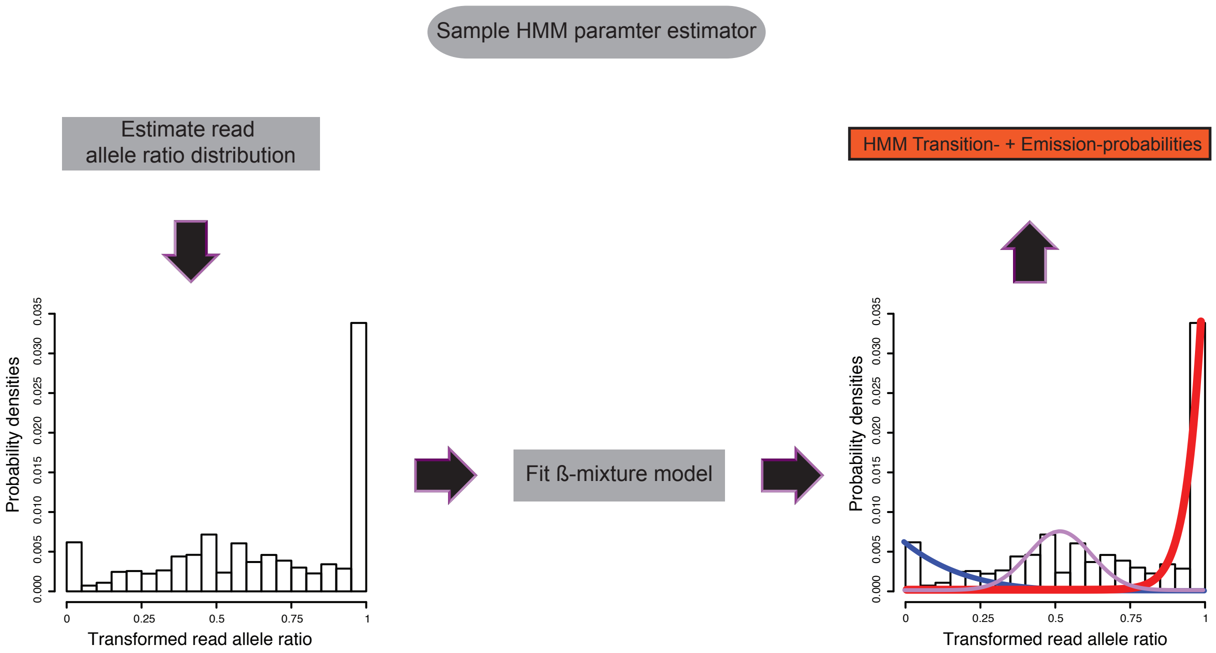

Figure S6 Schematic workflow of the parameter estimator for the HMM. For each sample the read allele ratio is estimated from the result of a sliding window approach. The resulting frequencies are then transformed to interval of 0 and 1 as the beta function is only defined at that region. Afterwards the beta-mixture model fit is applied. The probabilities for the sample-specific HMM are estimated from the resulting three curves and the output from the Basecaller.
