# Supplementary material for: Rapid and Inexpensive Whole-Genome Genotyping-by-Sequencing for Crossover Localization and Fine-Scale Genetic Mapping
Source: G3 (Bethesda). 2015 Jan 13;5(3):385–98. doi: 10.1534/g3.114.016501 (PMC4349092; doi:10.1534/g3.114.016501)
Supplement: Supporting Information [file supp_g3.114.016501_FigureS7.pdf]

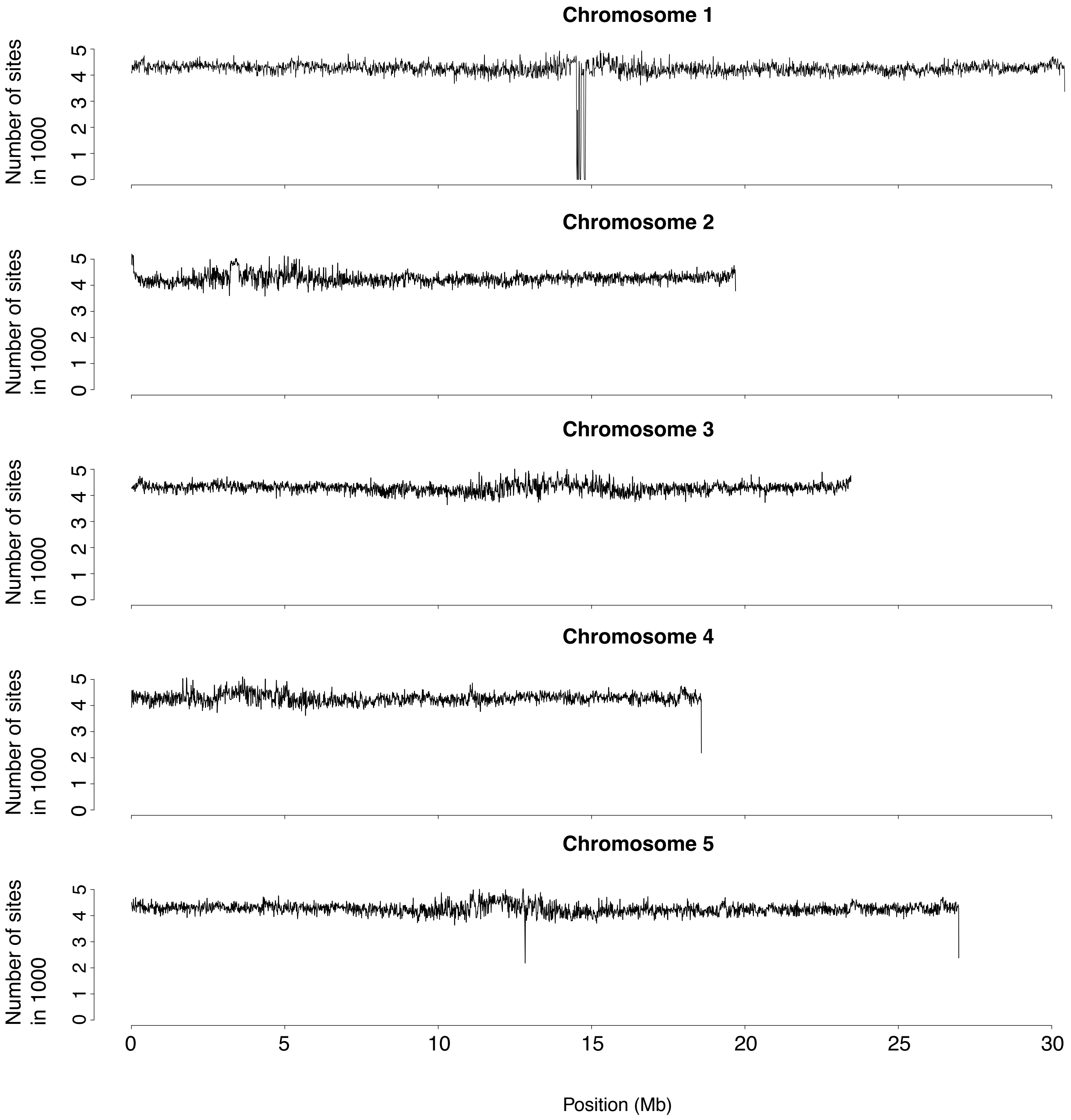

Figure S7 Distribution of dsDNA Shearase™ cutting sites across the genome. The enzyme recognizes a site with the degenerate sequence 5' NVBN 3', where N = any base, V = any base except for T, and B = any base except for A. The frequency of recognition sites across the *A. thaliana* genome over a 10 kb sliding window is shown.
