# Supplementary material for: Rapid and Inexpensive Whole-Genome Genotyping-by-Sequencing for Crossover Localization and Fine-Scale Genetic Mapping
Source: G3 (Bethesda). 2015 Jan 13;5(3):385–98. doi: 10.1534/g3.114.016501 (PMC4349092; doi:10.1534/g3.114.016501)
Supplement: Supporting Information [file supp_g3.114.016501_FigureS8.pdf]

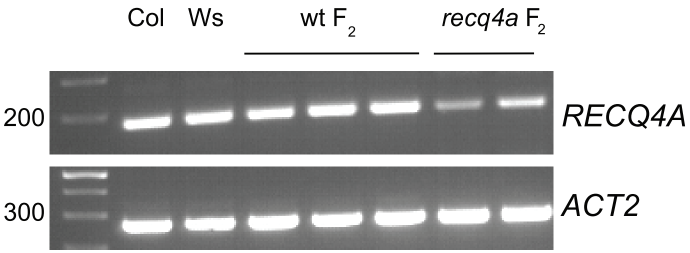


**Figure S8** Expression of *RECQ4A* transcripts in wt parents, wt $F_2$ , and *recq4a* $F_2$ individuals. All samples are mixed-stage flowers from a single individual. Numbers on the left indicate the size of the fragments (in base pairs). The *ACT2* gene was used as a control.
