# Supplementary material for: Rapid and Inexpensive Whole-Genome Genotyping-by-Sequencing for Crossover Localization and Fine-Scale Genetic Mapping
Source: G3 (Bethesda). 2015 Jan 13;5(3):385–98. doi: 10.1534/g3.114.016501 (PMC4349092; doi:10.1534/g3.114.016501)
Supplement: Supporting Information [file supp_g3.114.016501_FigureS9.pdf]

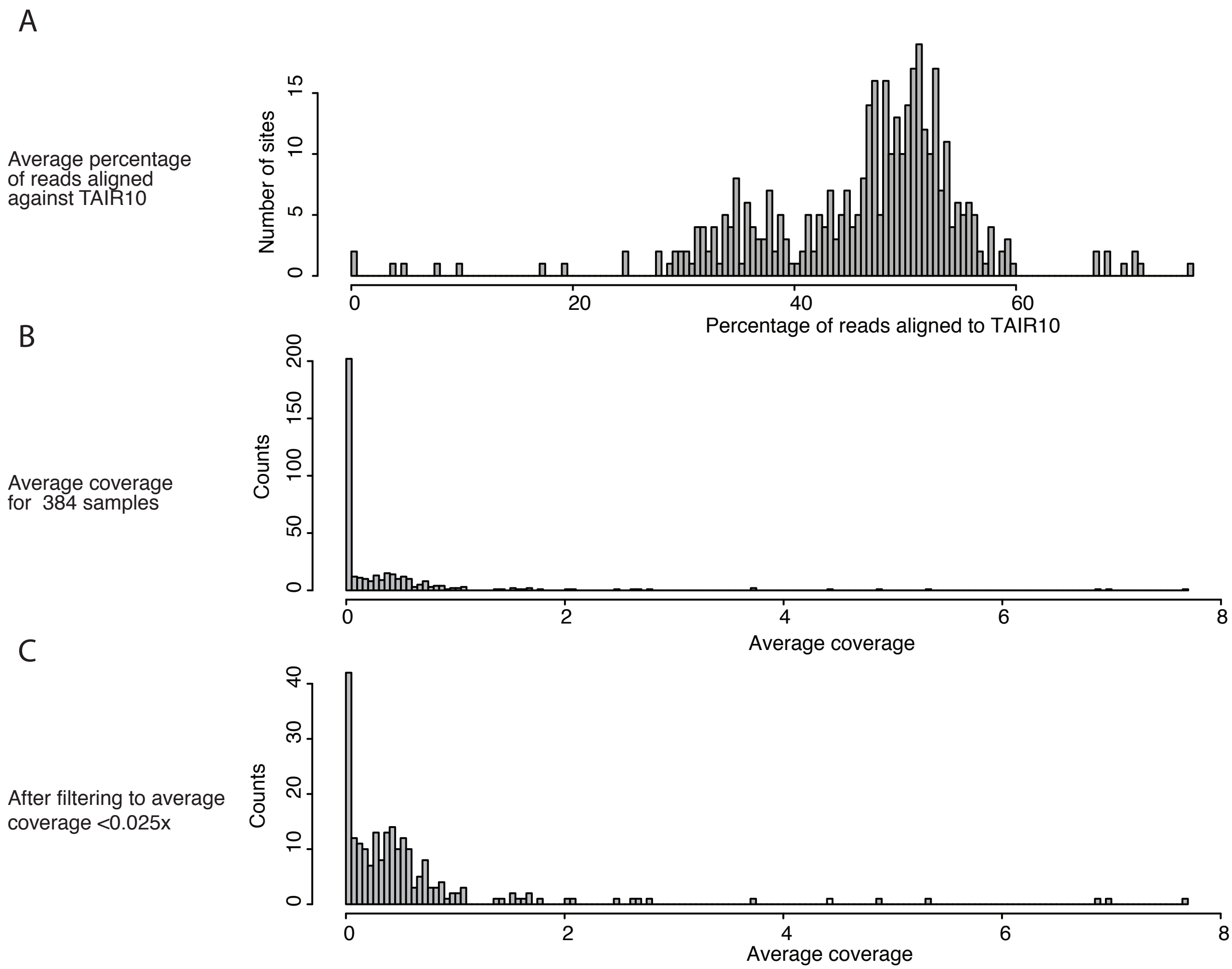

Figure S9 Coverage per sample and percentage of reads aligned. A) Average number of reads aligned to the used reference sequence TAIR10. The distribution of average read coverage (represented as the fold coverage of the *A. thaliana* reference genome) per sample is shown for all samples in B and for only samples where the average coverage was greater than 0.025x in C.
