# Supplementary material for: Rapid and Inexpensive Whole-Genome Genotyping-by-Sequencing for Crossover Localization and Fine-Scale Genetic Mapping
Source: G3 (Bethesda). 2015 Jan 13;5(3):385–98. doi: 10.1534/g3.114.016501 (PMC4349092; doi:10.1534/g3.114.016501)
Supplement: Supporting Information [file supp_g3.114.016501_FigureS10.pdf]

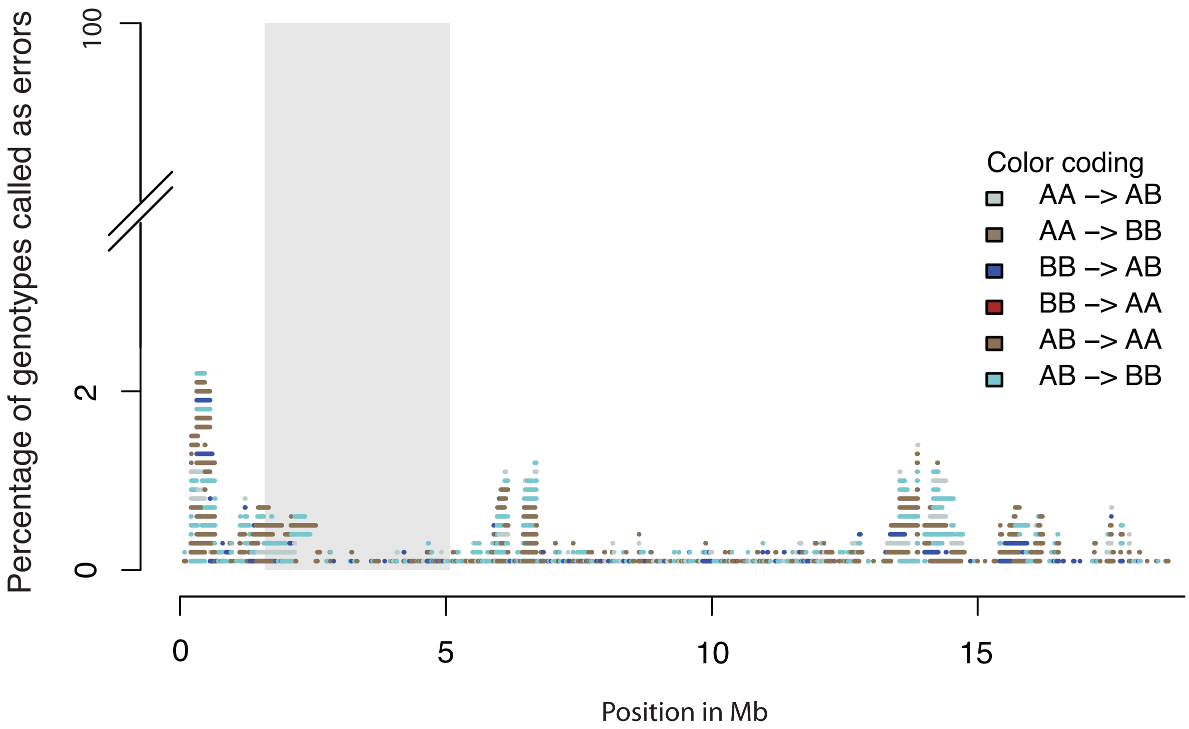

Figure S10 The frequency of different types of genotyping errors produced by TIGER using simulated data. An example of an error profile for chromsome 4 is shown (results were similar for the other four chromosomes). The grey box indicates the location of the centromere. The error frequencies are obtained from using TIGER to predict genotypes from random read data from 1000 simulated recombinant individuals.
