# Supplementary material for: Rapid and Inexpensive Whole-Genome Genotyping-by-Sequencing for Crossover Localization and Fine-Scale Genetic Mapping
Source: G3 (Bethesda). 2015 Jan 13;5(3):385–98. doi: 10.1534/g3.114.016501 (PMC4349092; doi:10.1534/g3.114.016501)
Supplement: Supporting Information [file supp_g3.114.016501_FigureS11.pdf]

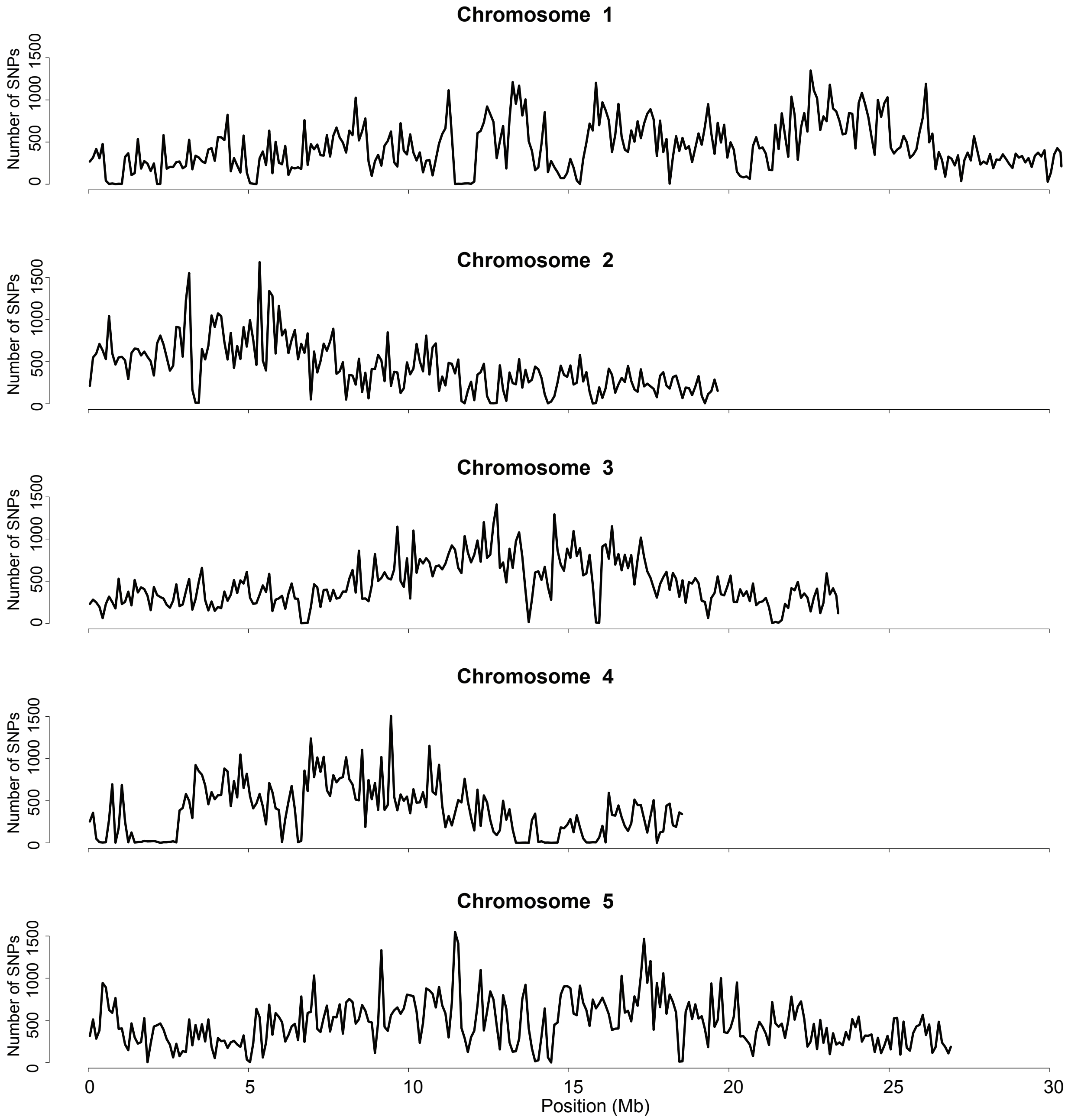

Figure S11 SNP density between Col-0 and Ws-2. The post-filtering SNP density (see Figure S4 for filtering parameters) using a sliding window of 100 kb is shown for each of the five *A. thaliana* chromosomes.
