# Supplementary material for: Rapid and Inexpensive Whole-Genome Genotyping-by-Sequencing for Crossover Localization and Fine-Scale Genetic Mapping
Source: G3 (Bethesda). 2015 Jan 13;5(3):385–98. doi: 10.1534/g3.114.016501 (PMC4349092; doi:10.1534/g3.114.016501)
Supplement: Supporting Information [file supp_g3.114.016501_FigureS12.pdf]

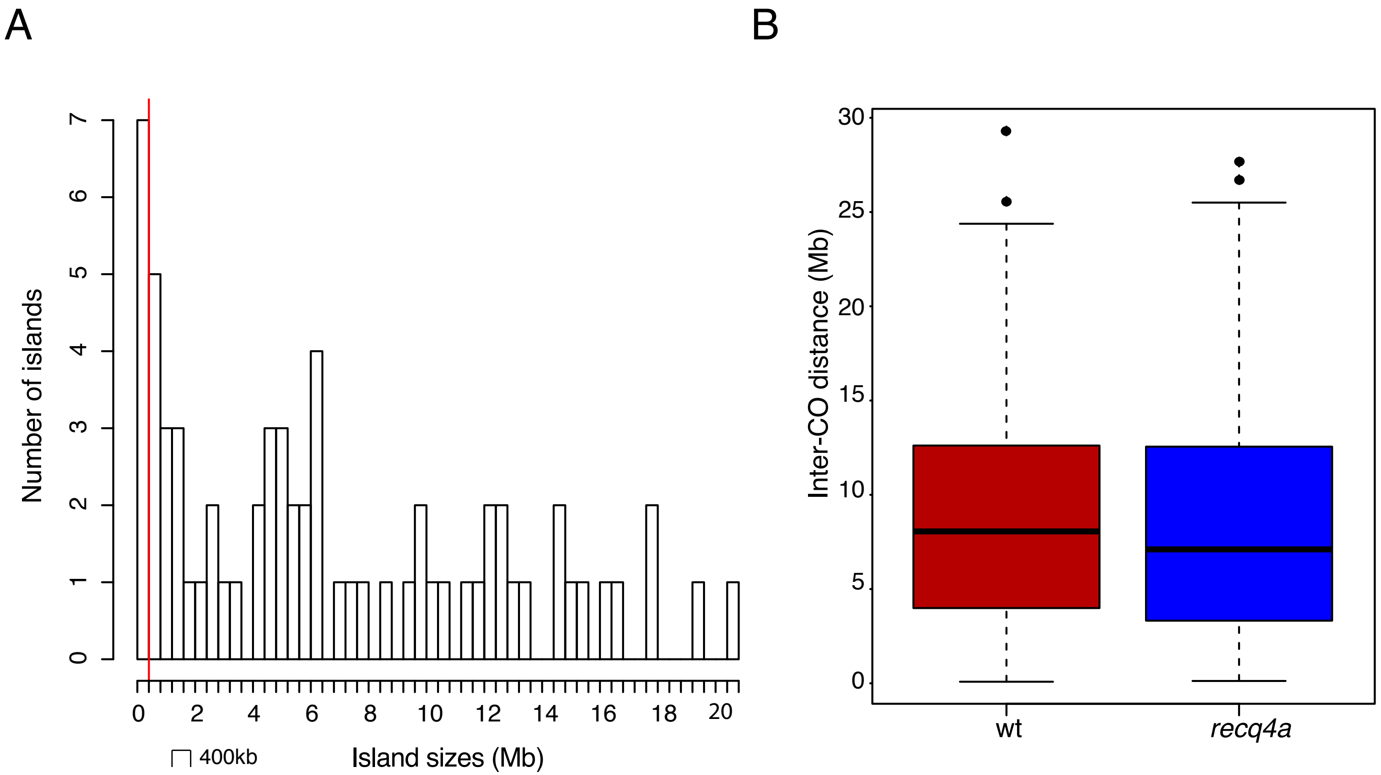

Figure S12 “Island” errors and double COs. TIGER-generated reconstructions of experimental recombinant individuals produced a type of error where a small genotype block was embedded in a larger block of a different genotype. A) Histogram depicting the frequency of the lengths of these small genotype “islands” is shown. Some of these islands are errors, others might represent real closely-spaced double COs. The red line indicates the chosen threshold for distinguishing between island errors and true double COs. B) Box plots showing the inter-CO distances for all true double COs in the wt compared with the mutant population.
