# Supplementary material for: Rapid and Inexpensive Whole-Genome Genotyping-by-Sequencing for Crossover Localization and Fine-Scale Genetic Mapping
Source: G3 (Bethesda). 2015 Jan 13;5(3):385–98. doi: 10.1534/g3.114.016501 (PMC4349092; doi:10.1534/g3.114.016501)
Supplement: Supporting Information [file supp_g3.114.016501_FigureS14.pdf]

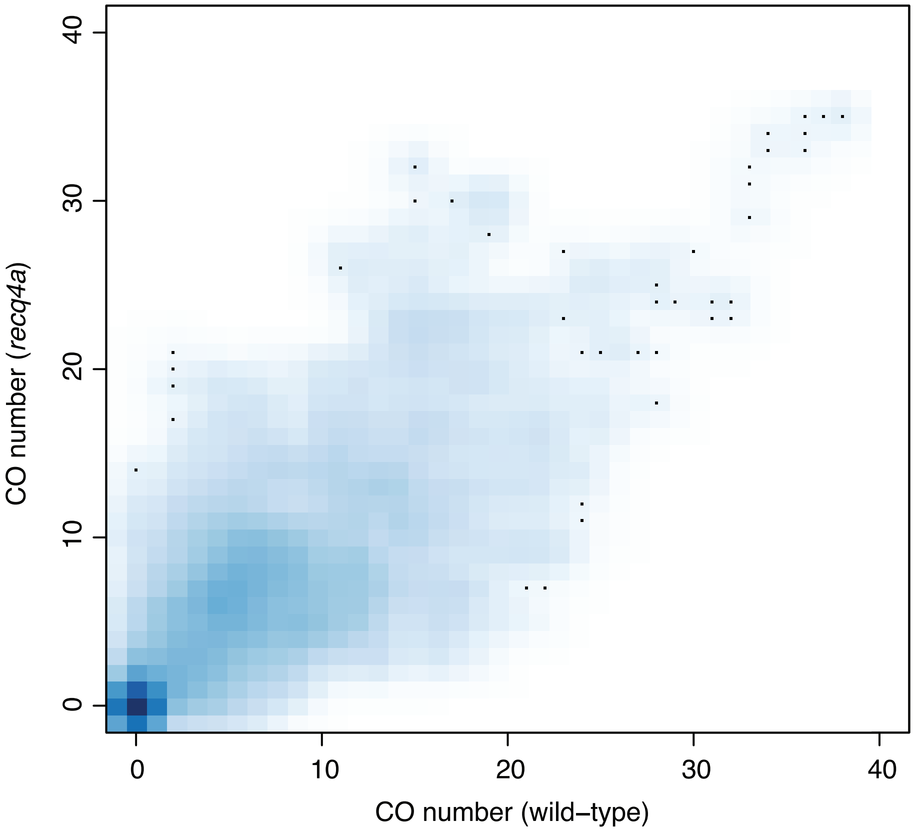

Figure S14 Correlation between CO distributions throughout the genome between wild-type and *recq4a* F_2 populations. Frequencies of 800-kb windows with the given numbers of COs are shown using a color scale from light (lowest) to dark (highest) blue.
