# Supplementary material for: Rapid and Inexpensive Whole-Genome Genotyping-by-Sequencing for Crossover Localization and Fine-Scale Genetic Mapping
Source: G3 (Bethesda). 2015 Jan 13;5(3):385–98. doi: 10.1534/g3.114.016501 (PMC4349092; doi:10.1534/g3.114.016501)
Supplement: Supporting Information [file supp_g3.114.016501_FigureS15.pdf]

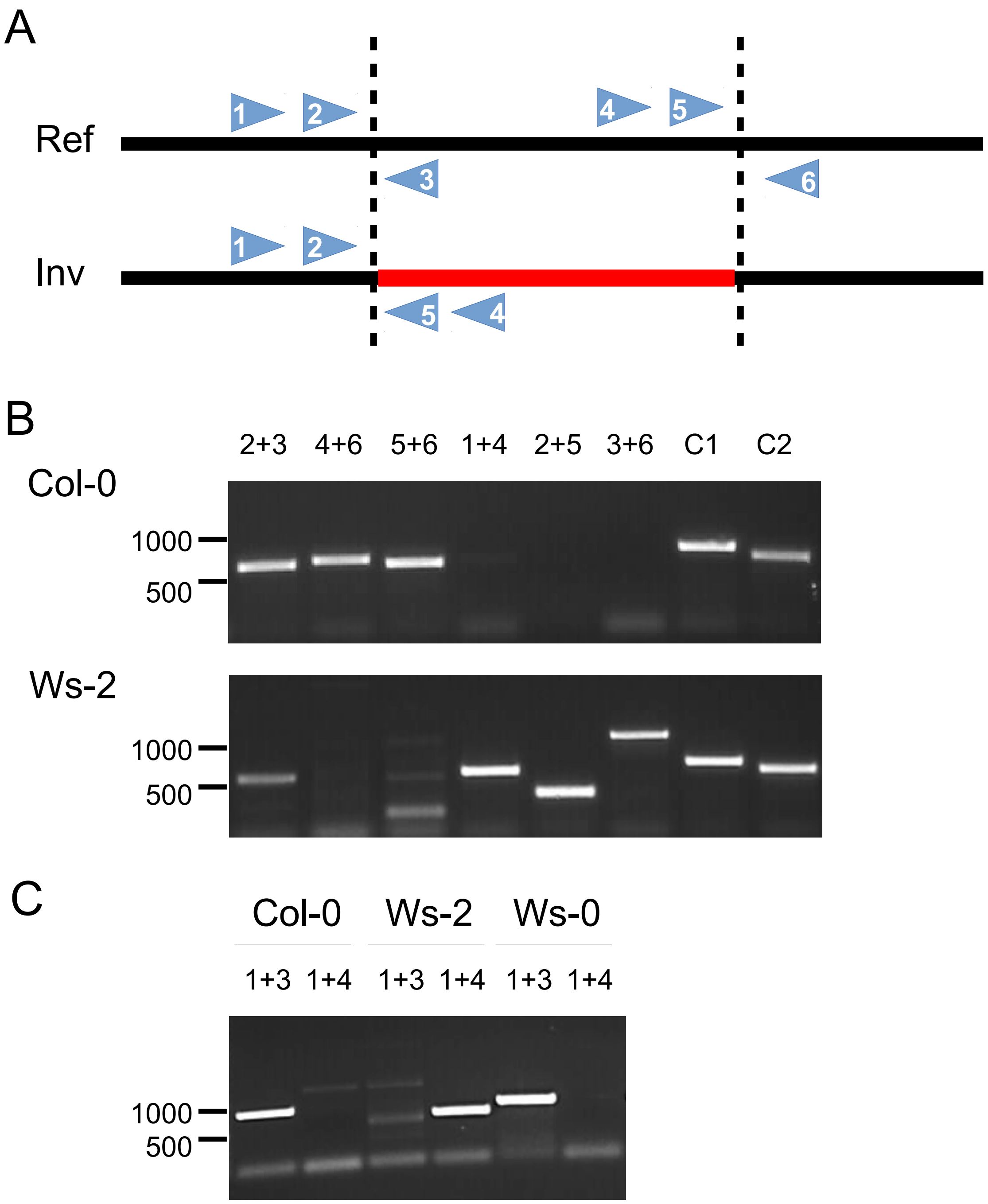

Figure S15 PCR amplification around predicted inversion breakpoints. (A) Schematic diagram showing the relative locations and orientations of primers around the predicted breakpoint positions (dashed vertical lines) at base positions 7139542 and 8914936 on Chr 4 (not drawn to scale). B) PCR bands amplified using the indicated combinations of the primers shown in A. "C1" is a control amplicon from Chr 5 (*MAF4*) and "C2" is a control amplicon from Chr 1. C) PCR bands amplified using the indicated combinations of the primers shown in A for Col-0, Ws-2 and Ws-0. The faint band visible for Ws-2 in the lane for primers 2+3 in B) is likely an off-target amplicon, as this primer set amplified bands of different sizes from Ws-2 DNA in replicate experiments at different annealing temperatures (data not shown).
