# Supplementary material for: Rapid and Inexpensive Whole-Genome Genotyping-by-Sequencing for Crossover Localization and Fine-Scale Genetic Mapping
Source: G3 (Bethesda). 2015 Jan 13;5(3):385–98. doi: 10.1534/g3.114.016501 (PMC4349092; doi:10.1534/g3.114.016501)
Supplement: Supporting Information [file supp_g3.114.016501_FigureS16.pdf]

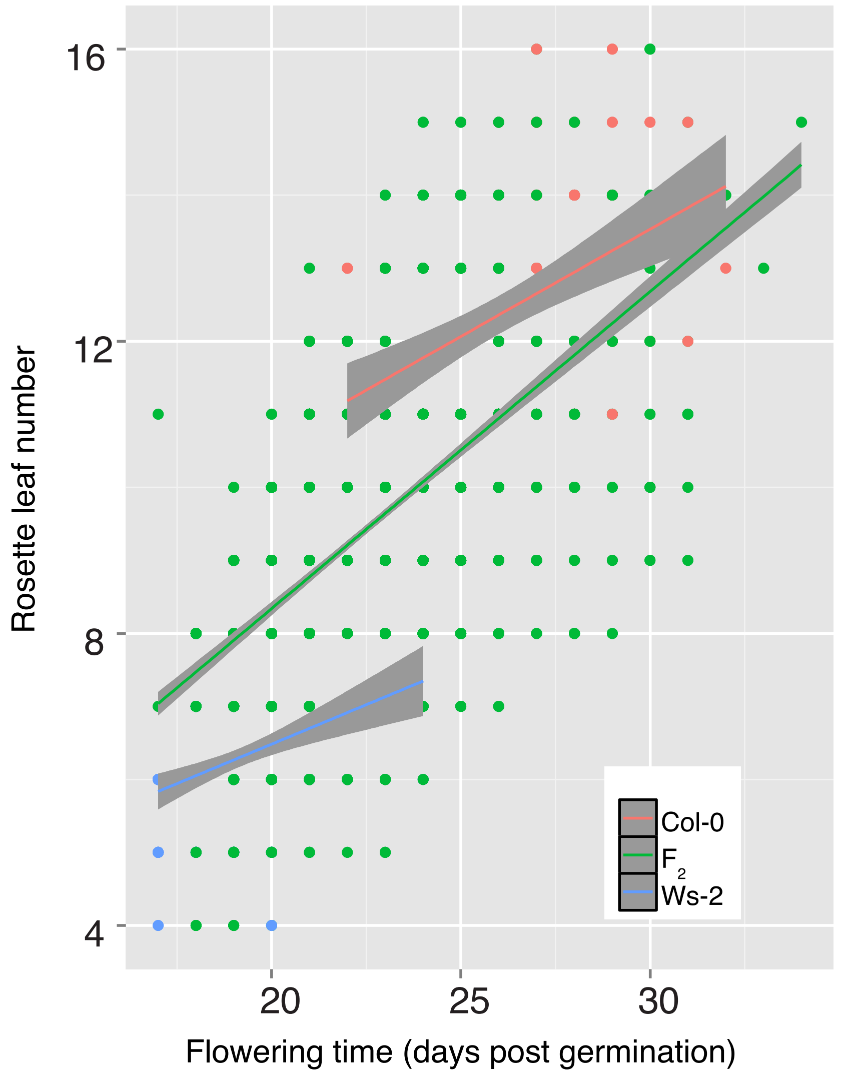

Figure S16 Correlation between the number of days to flower and the number of rosette leaves at flowering. R^2 = 0.18, 0.3, and 0.09 for Col-0, F, and Ws-2, respectively. N=129, 2185, and 202 for Col-0, F_2, and Ws-2, respectively. Equation of Col line: y = 0.29x + 4.7 R^2 = 0.18. Equation of F_2 line: y = 0.43x - 0.35 R^2 = 0.3. Equation of Ws-2 line: y = 0.22x + 2.3 R^2 = 0.09.
