# Supplementary material for: Rapid and Inexpensive Whole-Genome Genotyping-by-Sequencing for Crossover Localization and Fine-Scale Genetic Mapping
Source: G3 (Bethesda). 2015 Jan 13;5(3):385–98. doi: 10.1534/g3.114.016501 (PMC4349092; doi:10.1534/g3.114.016501)
Supplement: Supporting Information [file supp_g3.114.016501_FigureS17.pdf]

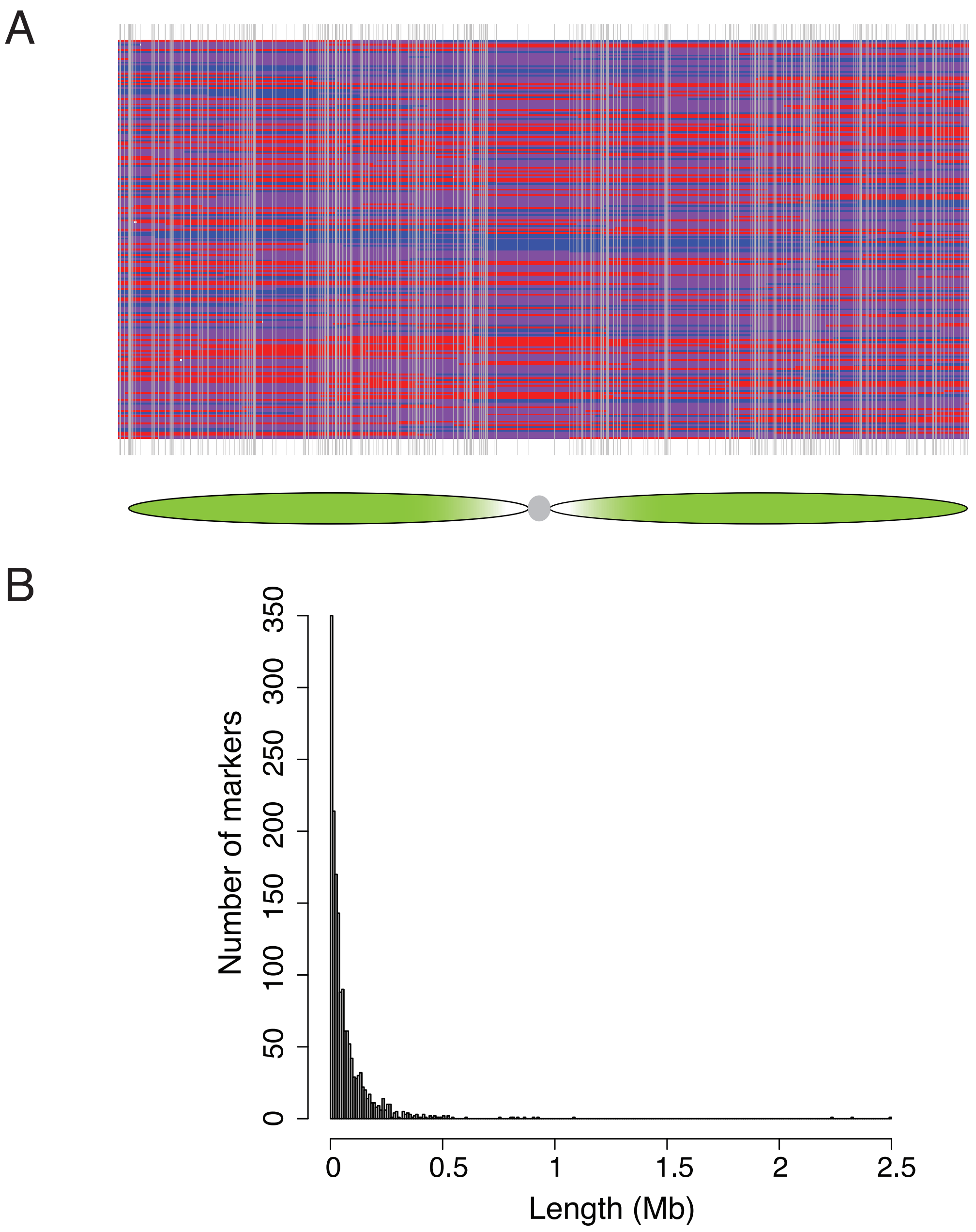

Figure S17 Recombination blocks used as markers for QTL analysis. A) A graphical representation of the recombination block that were usaed as markers for QTL mapping of flowering time in wt and *recq4a.* Graphical reconstructions of the genotypes along chromosome 1 are shown. Each horizontal line represents a single individual. Red indicates homozygous for Col-0, blue depicts homozygous for Ws-2 and heterozygous regions are in purple. The thin grey lines indicate the positions where at least one individual had a CO. SNPs in the regions between lines were used as markers for QTL analysis. B) Length distributions of the recombination
