# Supplementary material for: Rapid and Inexpensive Whole-Genome Genotyping-by-Sequencing for Crossover Localization and Fine-Scale Genetic Mapping
Source: G3 (Bethesda). 2015 Jan 13;5(3):385–98. doi: 10.1534/g3.114.016501 (PMC4349092; doi:10.1534/g3.114.016501)
Supplement: Supporting Information [file supp_g3.114.016501_FigureS18.pdf]

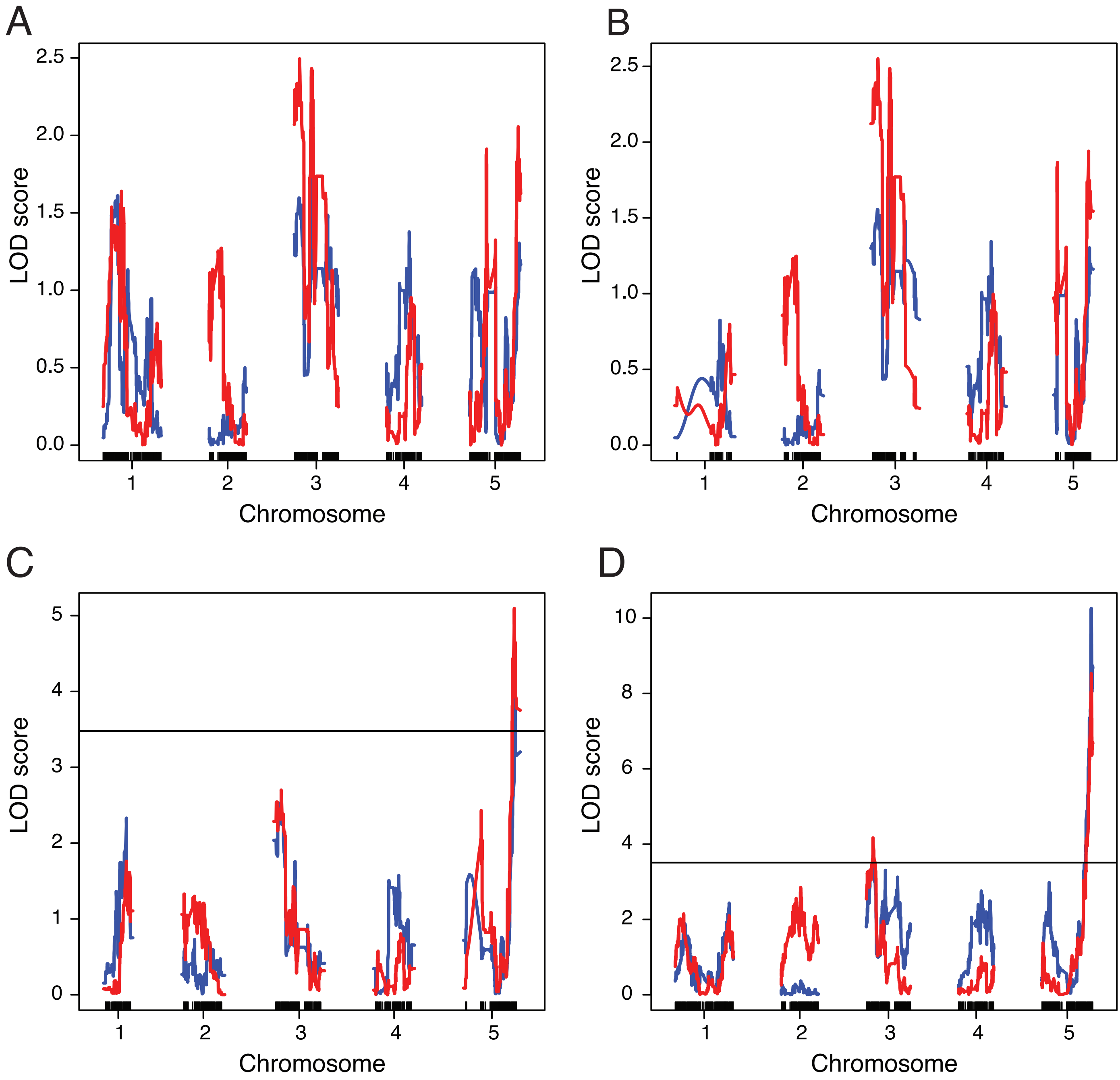

Figure S18 Additional plots showing the results of QTL analyses for flowering time. QTL analyses of the number of days to flowering (blue) and rosette leaf number (red) for all individuals of the wt population (A), all individuals of the wild-type population after dropping distorted markers (B), the wild-typet population with selected individuals and distorted markers dropped (C), and the combined wild-type and *recq4a* populations (D). Vertical ticks along the x-axis indicate the positions of the SNP markers genotyped. Horizontal lines indicate the position of the significance threshold for the population shown ($p < 0.05$ for 1000 permutations). Absence of a horizontal line indicates that no LOD scores passed the threshold.
